# EPA guidance on assessment of negative symptoms in schizophrenia

**DOI:** 10.1192/j.eurpsy.2021.11

**Published:** 2021-02-08

**Authors:** S. Galderisi, A. Mucci, S. Dollfus, M. Nordentoft, P. Falkai, S. Kaiser, G. M. Giordano, A. Vandevelde, M. Ø. Nielsen, L. B. Glenthøj, M. Sabé, P. Pezzella, I. Bitter, W. Gaebel

**Affiliations:** 1 Department of Psychiatry, Campania University Luigi Vanvitelli, Naples, Italy; 2 CHU de Caen, Service de Psychiatrie, 14000 Caen, France; 3 Normandie Univ, UNICAEN, ISTS EA 7466, GIP Cyceron, 14000 Caen, France; 4 Normandie Univ, UNICAEN, UFR de Médecine, 14000 Caen, France; 5 Copenhagen Research Centre for Mental Health (CORE), Copenhagen University Hospital, Copenhagen, Denmark; 6 Department of Clinical Medicine, Faculty of Health and Medical Science, University of Copenhagen, Copenhagen, Denmark; 7 Centre for Clinical Intervention and Neuropsychiatric Schizophrenia Research, CINS, Glostrup, Denmark; 8 Department of Psychiatry, University of Munich, Munich, Germany; 9 Division of Adult Psychiatry, Department of Psychiatry, Geneva University Hospitals, Geneva, Switzerland; 10 Center for Neuropsychiatric Schizophrenia Research, CNSR, Glostrup, Denmark; 11 Department of Psychiatry and Psychotherapy, Semmelweis University, Budapest, Hungary; 12 Department of Psychiatry and Psychotherapy, Medical Faculty, Heinrich-Heine University, Düsseldorf, Germany

**Keywords:** Assessment instruments, conceptualization, persistent negative symptoms, primary negative symptoms, secondary negative symptoms

## Abstract

**Background:**

During the last decades, a renewed interest for negative symptoms (NS) was brought about by the increased awareness that they interfere severely with real-life functioning, particularly when they are primary and persistent.

**Methods:**

In this guidance paper, we provide a systematic review of the evidence and elaborate several recommendations for the conceptualization and assessment of NS in clinical trials and practice.

**Results:**

Expert consensus and systematic reviews have provided guidance for the optimal assessment of primary and persistent negative symptoms; second-generation rating scales, which provide a better assessment of the experiential domains, are available; however, NS are still poorly assessed both in research and clinical settings.

This European Psychiatric Association (EPA) guidance recommends the use of persistent negative symptoms (PNS) construct in the context of clinical trials and highlights the need for further efforts to make the definition of PNS consistent across studies in order to exclude as much as possible secondary negative symptoms. We also encourage clinicians to use second-generation scales, at least to complement first-generation ones.

The EPA guidance further recommends the evidence-based exclusion of several items included in first-generation scales from any NS summary or factor score to improve NS measurement in research and clinical settings. Self-rated instruments are suggested to further complement observer-rated scales in NS assessment.

Several recommendations are provided for the identification of secondary negative symptoms in clinical settings.

**Conclusions:**

The dissemination of this guidance paper may promote the development of national guidelines on negative symptom assessment and ultimately improve the care of people with schizophrenia.

## Introduction

Negative symptoms have been recognized as a key component of schizophrenia since its first descriptions [1–3].

The conceptualization and descriptions of negative symptoms proposed by the 20th-century classic scholars [1–3] included two aspects: loss of motivation and reduction of emotional expression. The introduction of classification systems and operational criteria for diagnosis in psychiatry contributed to de-emphasizing the role of negative symptoms as a core aspect of schizophrenia, most likely due to a poorer inter-rater reliability in their assessment, as compared to positive symptoms. In spite of the predominant trend, the focus on negative symptoms kept alive by few research groups enabled further progress in the field [4–6]. The last decades witnessed a huge increase in the attention on negative symptom conceptualization. Main driver of the growing interest for negative symptoms in subjects with schizophrenia has been the evidence of their frequent occurrence and strong relationship with low remission rates, poor real-life functioning, and quality of life [4,5]. Large cross-sectional studies demonstrated that 50–60% of patients with schizophrenia have at least one negative symptom of moderate severity and approximately 10–30% of them experienced two or more, often enduring negative symptoms [4,7–11]. Furthermore, 50–90% of subjects with schizophrenia-spectrum disorders show negative symptoms during their first episode of the illness [12,13].

In the light of the strong impact on functional outcome and of the burden on patients, relatives, and health care systems, negative symptoms have become a key target of the search for new therapeutic tools. However, so far, progress in the development of innovative treatments has been slow and negative symptoms often represent an unmet need in the care of subjects with schizophrenia [4,6,14,15].

In 2005, the National Institute of Mental Health (NIMH) developed the Measurement and Treatment Research to Improve Cognition in Schizophrenia (MATRICS) initiative, which promoted a consensus conference aimed to review data on the existence of separate domains within negative symptoms and initiated a process for the development of evidence-based measures to improve their assessment. After 15 years from the consensus statement, negative symptoms are still poorly assessed and even when they are caused by known and treatable factors, such as extrapyramidal side effects, they are rarely recognized and properly treated.

To fill in this gap, the Schizophrenia Section of the European Psychiatric Association (EPA) proposed the development of a guidance paper aimed to provide recommendations for the assessment of negative symptoms in clinical trials and practice. The proposal was approved by the EPA Guidance Committee.

## Methodology

### Systematic literature search

The development of EPA guidance on the assessment of negative symptoms followed the standardized methods, according to the European Guidance Project of the EPA and to the Preferred Reporting Items for Systematic reviews and Meta-Analyses (PRISMA), as described in previous publications [16–20].

In brief, we performed a comprehensive literature search on the assessment of negative symptoms in subjects with schizophrenia. The search has been run in three electronic databases: Medline (PubMed), Scopus, and PsycINFO with no time limit, in order to ensure that it was as comprehensive as possible (Table 1).

**Table 1.** Systematic search strategies.

Database	Search syntax	Number of retrieved documents	Date of search
Medline (PubMed)	(Schizophrenia AND "negative symptoms") O R (Schizophrenia AND avolition) OR (Schizophrenia AND apathy) OR (Schizophrenia AND anhedonia) OR (Schizophrenia AND alogia) OR (Schizophrenia AND asociality) OR (Schizophrenia AND amotivation) OR (Schizophrenia AND "social withdrawal") OR (Schizophrenia AND "blunted affect") OR (Schizophrenia AND "affective flattening") OR (Schizophrenia AND “persistent negative symptoms”) OR (Schizophrenia AND “predominant negative symptoms”) OR (Schizophrenia AND “prominent negative symptoms”) OR (Schizophrenia AND "primary negative symptoms") OR (Schizophrenia AND “deficit schizophrenia”) OR (Schizophrenia AND “lack of motivation”)	6438	December 9, 2019
	Filters: Languages, English; Species, Human		
	Search in [Title/Abstract]		
	No time limit		
Scopus	(Schizophrenia AND "negative symptoms") OR (Schizophrenia AND avolition) OR (Schizophrenia AND apathy) OR (Schizophrenia AND anhedonia) OR (Schizophrenia AND alogia) OR (Schizophrenia AND asociality) OR (Schizophrenia AND amotivation) OR (Schizophrenia AND "social withdrawal") OR (Schizophrenia AND "blunted affect") OR (Schizophrenia AND "affective flattening") OR (Schizophrenia AND “persistent negative symptoms”) OR (Schizophrenia AND “predominant negative symptoms”) OR (Schizophrenia AND “prominent negative symptoms”) OR (Schizophrenia AND "primary negative symptoms") OR (Schizophrenia AND “deficit schizophrenia”) OR (Schizophrenia AND “lack of motivation”)	9863	December 9, 2019
	Filters: Languages, English; Species, Human		
	Search in [Title/Abstract/Keywords]		
	No time limit		
PsychINFO	(Schizophrenia AND "negative symptoms") OR (Schizophrenia AND avolition) OR (Schizophrenia AND apathy) OR (Schizophrenia AND anhedonia) OR (Schizophrenia AND alogia) OR (Schizophrenia AND asociality) OR (Schizophrenia AND amotivation) OR (Schizophrenia AND "social withdrawal") OR (Schizophrenia AND "blunted affect") OR (Schizophrenia AND "affective flattening") OR (Schizophrenia AND “persistent negative symptoms”) OR (Schizophrenia AND “predominant negative symptoms”) OR (Schizophrenia AND “prominent negative symptoms”) OR (Schizophrenia AND "primary negative symptoms") OR (Schizophrenia AND “deficit schizophrenia”) OR (Schizophrenia AND “lack of motivation”)	10481	December 9, 2019
	Filters: Languages, English; Species, Human		
	Search in [Title/Abstract/Keywords]		
	No time limit		

Studies were selected according to predefined inclusion and exclusion criteria as follows:

### Inclusion criteria

1. meta-analysis, randomized controlled trial, review, cohort study, open study, descriptive study, expert opinion, concerning conceptualization, and assessment of negative symptoms in subjects with schizophrenia according to the search terms cited in Table 1;
2. studies published in English;
3. studies carried out in humans;
4. studies published in journals indexed in Embase or Medline.

### Exclusion criteria

1. duplicates, comments, editorials, case reports/ case series, theses, proceedings, letters, short surveys, and notes;
2. studies irrelevant for the topic, including studies relevant to the treatment of negative symptoms;
3. studies concerning exclusively pathophysiological mechanisms of negative symptoms (those reporting imaging or electrophysiological or other biomarker correlates of negative symptoms);
4. unavailable full-text;
5. studies that do not meet inclusion criteria.

Discrepancies in the selection and any change in methodology have been discussed in advance with the whole group. In particular, a deviation from the methodology has been taken for the following sections: “*Assessment of negative symptoms in first episode psychosis (FEP) patients” and “Assessment of negative symptoms in clinical high risk (CHR) individuals*”.

With regard to FEP studies, an additional search on Medline was performed on December 18, 2019 following the search strategy described in Table 1 and the inclusion and exclusion criteria listed above, replacing the term “schizophrenia” with the term “first episode schizophrenia”. The literature was then screened focusing on the topic “assessment” in FEP. Due to the enormous amount of literature using the original summed scores of the Positive and Negative Syndrome Scale (PANSS) and of the Scale for the Assessment of Negative Symptoms (SANS), these studies have been excluded and have been represented by meta-analyses only. Studies described individually in paragraph 4.2 used factor models or sub-scores from PANSS or SANS, or other assessment instruments, or focused on primary negative symptoms, persistent negative symptoms, or deficit syndrome (DS). Of the relevant references for this topic, 23 studies had been already included in the original search.

With regard to CHR studies, an additional search on Medline was performed on December 16 and 17, 2019 following the search strategy described in Table 1 and the inclusion and exclusion criteria listed above, replacing the term “schizophrenia” with the terms “ultra-high risk psychosis”; “clinical high risk psychosis”; “prodromal psychosis”. To narrow the search, only intervention studies using a negative symptom outcome were included. Of the relevant references for this topic, 17 studies had been already included in the original search.

**Figure 1.**
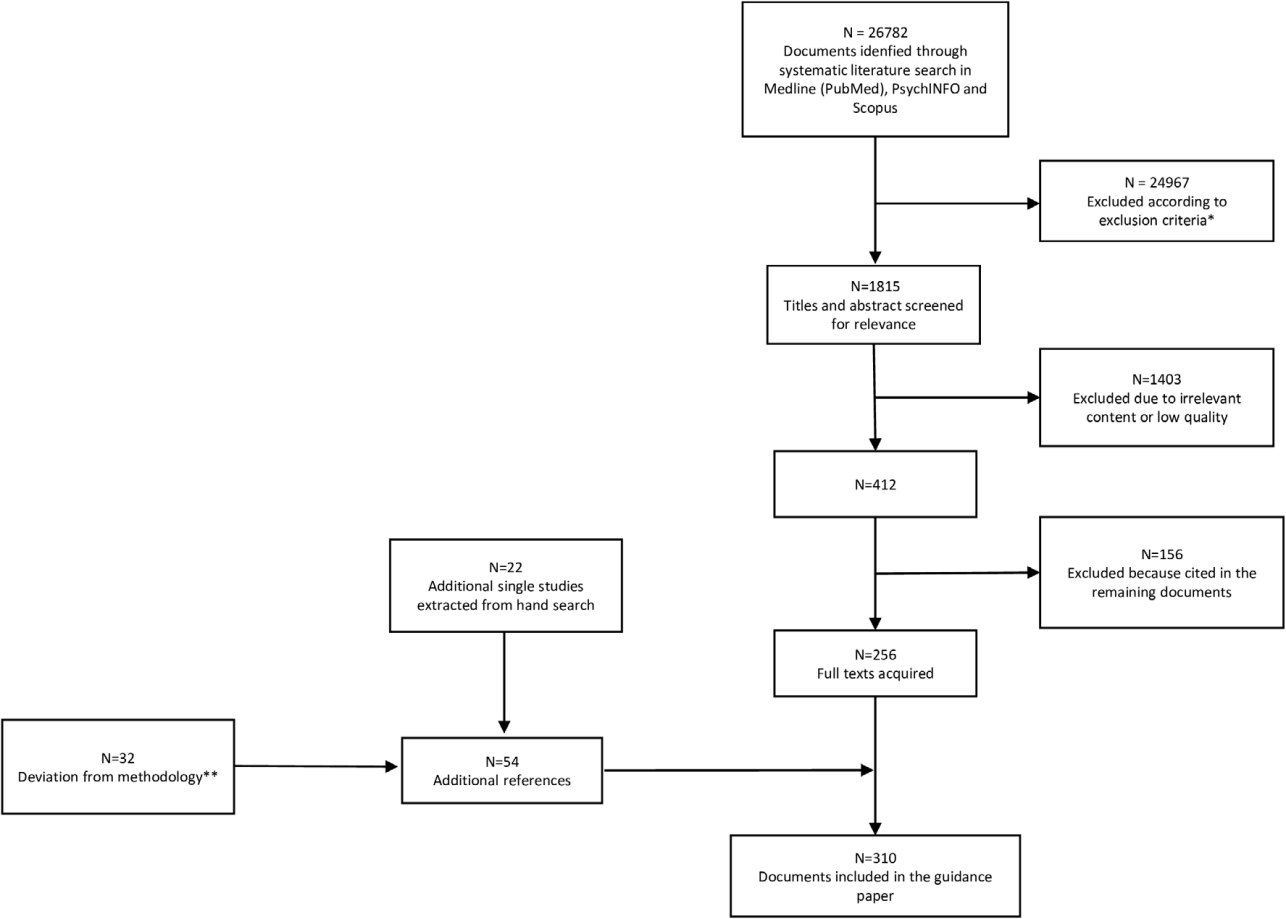
PRISMA flowchart of studies retrieved in the systematic literature search. *11905 duplicates; 1826 studies other than meta-analysis, randomized controlled trial, review, cohort study, open study, descriptive study, expert opinion; 843 studies published in journal not indexed in Embase or Medline; 2895 studies on pathophysiological mechanisms of negative symptoms; 5813 articles not related to any topic; 1527 articles related to the treatment of negative symptoms; 158 studies conducted in animals. **The deviation from the original search regarded the Sections: “Assessment of negative symptoms in First Episode Psychosis patients” (*N* = 8; the other 23 had been already included in the 256 documents of the original search) and “Assessment of negative symptoms in clinical high risk individuals” (*N* = 24; the other 17 had been already included in the 256 documents of the original search).

Details of the selection process are shown in Figure 1.

Included studies have been graded for the level of evidence, according to the previous literature [20].

For all documents, evidence grades were assigned according to Gaebel et al., 2017 [21] (Table 2). Based on the evidence level of the included studies, recommendations were developed by three authors (SG, AM, and SD) and reviewed by all coauthors. Discrepancies in the ratings were resolved by discussion among all coauthors. Each recommendation level was then graded following Gaebel et al., 2017 [21] (Table 3).

**Table 2.** Grading of evidence.

Grade	Features of quantitative studies	Features of reviews
I-Generalizable studies	Randomized controlled trials. Surveys sampling a large and representative group of persons from the general population or from a large range of service settings. Analytic procedures comprehensive and clear usually including multivariate analyses or statistical modeling. Results can be generalized to settings or stakeholder groups other than those reported in the study	Systematic reviews or meta-analyses
II-Conceptual studies	Uncontrolled, blinded clinical trials. Surveys sampling a restricted group of persons or a limited number of service providers or settings. May be limited to one group about which little is known or a number of important subgroups. Analytic procedures comprehensive and clear. Results have limited generalizability	Unsystematic reviews with a low degree of selection bias employing clearly defined search strategies
III-Descriptive studies	Open, uncontrolled clinical trials. Description of treatment as usual. Survey sampling not representative since it was selected from a single specialized setting or a small group of persons. Mainly records experiences and uses only a limited range of analytical procedures, like descriptive statistics. Results have limited generalizability	Unsystematic reviews with a high degree of selection bias due to undefined or poorly defined search strategies
IV-Single case study	Case studies. Provides survey data on the views or experiences of a few individuals in a single setting. Can provide insight in unexplored contexts. Results cannot be generalized	Editorials

Note. Modified from Gaebel et al., 2017 [21] .

**Table 3.** Grading of recommendations.

Grade	Description
A	At least on study or review rated as I and directly applicable to the target population OR a body of evidence consisting principally of studies and/or reviews rated as I, directly applicable to the target population, and demonstrating overall consistency of results
B	A body of evidence including studies and/or reviews rated as II, directly applicable to the target population, and demonstrating overall consistency of results OR extrapolated evidence from studies and/or reviews rated as I or II
C	A body of evidence including studies and/or reviews rated as II–III, directly applicable to the target population, and demonstrating overall consistency of results OR extrapolated evidence from studies and/or reviews rated as II or III
D	Level of evidence rated as III or IV OR extrapolated evidence from studies and/or reviews rated as III or IV OR expert consensus

Note. Modified from Gaebel et al., 2017 [21].

## Conceptualization

Based on the review of data relevant to the construct validity of negative symptoms [22], the NIMH-MATRICS consensus statement on negative symptoms [23,24] identified five main domains of negative symptoms: anhedonia, avolition, blunted affect, alogia, and asociality [4,5,22,23]. A brief description of each symptom domain according to the consensus statement is provided in Box 1.

Box 1. Definition of negative symptoms based on the NIMH-MATRICS consensus statement [23].

- **Avolition:** a reduction in the initiation and persistence of goal-directed activities due to a lack of motivation.
- **Anhedonia:** a reduction in the experience of pleasure during the activity (consummatory anhedonia) and for future anticipated activities (anticipatory anhedonia).
- **Asociality:** a reduction in social interactions due to a reduced drive to form and maintain relationships with others.
- **Blunted affect:** a reduction in the expression of emotion in terms of facial and vocal expression, as well as body gestures.
- **Alogia:** a reduction in quantity of words spoken and amount of spontaneous elaboration.

Understanding the possible associations between these domains has important implications in the design of clinical trials. For instance, if we assume that these domains represent a single construct with the same neurobiological underpinnings, they should respond to the same treatment, and a separate assessment of each of them would be redundant. On the contrary, if these domains are independent from each other or cluster into a limited number of factors, they might respond differently to treatment, and therefore a separate assessment of each of the domains or factors would be necessary [23]. The consensus statement suggested that, although the five negative symptom domains were interrelated, there was an important degree of independence between them. In the light of the definitions of the five domains, the development of new instruments that could properly assess them was recommended. In fact, the two most used scales, the SANS [25] and the PANSS [26], include aspects that are not part of negative symptom domains, do not allow the differentiation between anticipatory and consummatory anhedonia, and only focus on patient’s behavior, failing to assess subject’s internal experience, that is crucial for the evaluation of experiential deficits, such as anhedonia, avolition, and asociality [4,5,23,27–30]. Based on these recommendations, two new instruments were developed, the Brief Negative Symptom Scale (BNSS) and the Clinical Assessment Interview for Negative Symptoms (CAINS) [28–30]. For a more detailed description of these instruments, please refer to the section on assessment.

### Classification of negative symptoms

Negative symptoms represent a heterogeneous dimension, including symptoms with different causes and course, and, therefore, possibly requiring different treatment management [4,5,14,22,31–41]. Different approaches to the negative symptom classification have been pursued in order to reduce their heterogeneity, not only in the research context, but also in the context of clinical trials.

#### Primary and secondary negative symptoms

The distinction between primary and secondary negative symptoms has important research and clinical implications [4,33,35,39,41]. Primary negative symptoms are thought to stem from the pathophysiological substrate underlying schizophrenia, while secondary negative symptoms might be caused by positive symptoms, depression, medication side effects, social deprivation, and substance abuse [4,33,35,39,41]. Secondary negative symptoms might be responsive to the treatment of the underpinning causes. For instance, negative symptoms secondary to depression or to positive symptoms might be responsive to antidepressant and antipsychotic treatments, respectively. In addition, the failure to differentiate primary from secondary negative symptoms is likely to hinder progress in innovative treatment discoveries [4]. For a detailed description of differential diagnosis between primary negative symptoms and secondary ones, please consult the dedicated section.

#### The Deficit Syndrome

In 1988, Carpenter and colleagues introduced the concept of DS to characterize schizophrenia with primary and enduring negative symptoms [31]. The diagnostic criteria for the DS are reported in Box 2.

Box 2. Diagnostic criteria for the Deficit Syndrome [31, 42].

- Presence of at least two out of the following six negative symptoms:
  - Restricted affect: expressionless face, reduced expressive gestures, and diminished modulation of the voice.
  - Diminished emotional range: the intensity and range of a person’s (subjective) emotional experience.
  - Poverty of speech: reduced number of words used and the amount of information conveyed.
  - Curbing of interests: the degree to which the person is interested in the world around him or her, both ideas and events.
  - Diminished sense of purpose: the degree to which the person posits goals for his/her life; the extent to which the person fails to initiate or sustain goal-directed activity due to inadequate drive; the amount of time passed in aimless inactivity.
  - Diminished social drive: degree to which the person seeks or wishes for social interaction.
- Presence of the above symptoms for at least 12 months including periods of clinical stability.
- The above symptoms are primary and not secondary to factors such as anxiety, drug effect, positive symptoms, mental retardation, and depression.
- The patient meets DSM (third or later edition) criteria for schizophrenia.

To date, the validity of this construct is supported by data collected in nine reviews [4,14,32,34,36,38,39,43,44] (Table e1). The first review [32] supported the construct validity of the diagnosis, based on the cohesiveness of the symptoms used for its definition. Evidence was also provided that DS may represent a separate disease entity with respect to non-deficit schizophrenia (NDS), as the two entities differ in terms of signs and symptoms, course of illness, risk factors, biological correlates, and treatment response. These differences are not confounded by demographic features, antipsychotic treatment, severity of psychotic symptoms, or drug abuse. The review also supports the view that DS is not just a more severe form of the disease, as its characteristics and correlates are not just more of the same observed in NDS. The construct validity of the DS and the distinction between DS and NDS were also supported by subsequent reviews [4,14,34,36,38,39,43,44]. Notwithstanding the large consensus on the validity of this construct, some studies reported discrepant findings regarding differences between DS and NDS in terms of clinical and neurobiological features [14,34,36,38,43]. Three reviews [36,38,43] suggested that heterogeneity within the DS might complicate the diagnosis of DS.

The gold standard instrument to assess DS is the Schedule of Deficit Syndrome (SDS) [42]. The correspondence between negative symptoms included in the SDS with the MATRICS domains, as well as the assessment procedures are reported in Box 3.

Box 3. Negative symptoms included in the Schedule for the Deficit Syndrome (SDS): correspondence with the MATRICS domains and assessment procedures [42].

**Table:** 

SDS item	Comparative NIMH-MATRICS domain	Procedures
Restricted affect	Blunted affect	This SDS item evaluates the reduced expressive gestures, modulation of voice, and changes in facial expression. These aspects are rated on the basis of what is observed during the interview and eventually confirmed by other sources of information (i.e., caregiver).
Diminished emotional range	--	This SDS item evaluates the reduced ability to experience pleasure as well as the lack of dysphoria of any kind (in terms of range and intensity). The reduced pleasure due to abnormal perceptions would not be considered as diminished emotional range.
Poverty of speech	Alogia	This SDS item is rated on the basis of behavior during the interview. The poverty of content of speech is not rated here.
Curbing of interests	Avolition	The rating for this SDS item is based on both patient’s behavior and thoughts. The patient may display a diminished range of interests or a diminished depth of interests; either impairment may be considered pathological. The reduced interest due to a pathological preoccupation with psychotic features would not be considered as curbing of interests.
Diminished sense of purpose	Avolition	This SDS item evaluates: (a) the degree to which the patient posits goals for his/her life; (b) the extent to which the patient fails to initiate or sustain goal-directed activities due to an inadequate drive; and (c) the amount of time spent in aimless inactivity. Whether or not the goal is realistic is not relevant.
Diminished social drive	Asociality	The rating considers patient’s internal experience, statements, and behaviors. This SDS item is not equivalent to social withdrawal, and social success is not rated here. The avoidant patient, who longs for social contacts and occasionally seeks it but is made uncomfortable by it, is not regarded as having diminished social drive.

SDS has a good inter-rater reliability within research groups, but requires extensive training, the use of different sources of information, and a careful longitudinal clinical evaluation to judge whether the observed negative symptoms are primary or secondary [14,32,34,36,38, 44]. The last information is not always available, especially in first episode patients [14,34,36,44].

To increase the practicability of the DS diagnosis, a proxy [45–47] was developed based on the Brief Psychiatric Rating Scale (BPRS) [48], PANSS [26], or SANS [25]. The proxy allows the categorization of a large number of patients included in existing datasets in which the SDS was not used. However, in spite of its good sensitivity and specificity, several concerns on face validity of these measures have been raised [36,49]. Another concern is relevant to the lack of temporal stability of the DS categorization made with the proxy, since a longitudinal study did not confirm the stability of the categorization (DS vs NDS) at 1-year follow-up [50]. Given the above-mentioned limits, further studies are needed before the use of proxy measures can be recommended. These studies should assess negative symptoms with second-generation rating scales (BNSS and CAINS) and validate the specific cutoff for the DS/NDS categorization in different samples. The available evidence does not allow recommending the use of a proxy for the DS/NDS categorization.

#### Persistent, predominant, and prominent negative symptoms

In the light of the above observations, the consensus statement on negative symptoms suggested a focus on persistent negative symptoms, that is, negative symptoms that persist over time, including periods of clinical stability, despite an adequate antipsychotic drug treatment [23,44]. Criteria for persistent negative symptoms are reported in box 4.

Box 4. Criteria for “persistent negative symptoms” [44].

- Presence of at least moderate* for at least three negative symptoms, or at least moderately severe** for at least two negative symptoms.
- Defined threshold levels of positive symptoms, depression, and extrapyramidal symptoms on accepted and validated rating scales.
- Persistence of negative symptoms for at least 6 months.

*e.g., a score of 4 on the Positive and Negative Syndrome Scale (PANSS) or a score of 3 on the Brief Negative Symptom Scale (BNSS); **e.g., a score of 5 on the PANSS or a score of 4 on the BNSS.

To date, the validity of this construct is supported by data collected in four reviews [4,14,36,44] (Table e1), which suggest that the persistent negative symptom construct identifies a patient population larger than the one with DS and allows the control of potential sources of indirect changes of negative symptoms during the course of clinical trials. However, concerns on the persistent negative symptom construct have also been raised: the construct allows the use of any validated psychopathological rating scale, including those scales, such as SANS and PANSS, that include items not relevant to the negative symptom dimension; threshold for confounding factors (positive, depressive, and extrapyramidal symptoms) are not uniquely defined across studies [4,14,36].

In clinical trials, as requested by regulatory agencies, in order to evaluate the efficacy of drugs for negative symptoms, other two concepts have been used: “predominant negative symptoms” and “prominent negative symptoms” (Boxes 5 and 6 for criteria). Neither construct included the evaluation of persistence over time of negative symptoms.

Box 5. Criteria for “predominant negative symptoms”

(A)

1. Presence of at least moderate* for at least three symptoms or at least moderately severe** for at least two symptoms [51] or
2. Any score on PANSS negative subscale but at least 6 points greater than the PANSS positive subscale score [52] or
3. PANSS Negative subscale score of at least 21 and at least 1 point greater than the PANSS positive subscale score [53] or
4. PANSS negative subscale score greater than the PANSS positive subscale score [54].

(B)

1. Positive PANSS subscale score less than 19, depressive and extrapyramidal symptoms lower than a defined threshold on a validated rating scale [51] or
2. Severity of positive, depressive, and extrapyramidal symptoms not specified [52–54].

*e.g., a score of 4 on the Positive and Negative Syndrome Scale (PANSS); **e.g., a score of 5 on the PANSS.

Box 6. Criteria for “prominent negative symptoms” [51, 54]

Presence of at least moderate* for at least three symptoms or at least moderately severe** for at least two symptoms on the PANSS negative subscale.

*e.g., a score of 4 on the Positive and Negative Syndrome Scale (PANSS); **e.g., a score of 5 on the PANSS.

Three reviews [4,14,36] analyzed data on “predominant negative symptoms” and only one of these reviews focused on “prominent negative symptoms” too [36] (Table e1). These two concepts were also discussed during an international meeting, involving experts in the field, who did not reach an agreement on whether predominant or prominent negative symptoms should be considered in clinical trials [55] (Table e1). Available evidence and expert opinions suggest the following: (a) both these concepts include a mixture of primary and secondary negative symptoms likely to fluctuate over time and possibly confounding the results of clinical trials; (b) no construct validity was supported; (c) no consensus was achieved on strategies to reduce the heterogeneity in the definition of predominant negative symptoms.

To conclude, available evidence shows that DS and persistent negative symptoms have construct validity and have several advantages over negative symptoms broadly defined for isolating those negative symptoms that still represent an unmet therapeutic need. Compared to the DS, the persistent negative symptom construct has the advantage to be more easily applicable in the context of clinical trials: (a) potential sources of secondary negative symptoms are not excluded as much as in DS, but the persistent negative symptom construct enables the control of the main confounding factors; (b) the construct includes secondary negative symptoms that have not responded to previous treatments; (c) persistent negative symptoms identify a patient population larger than the one with DS; (d) the identification of these symptoms requires less longitudinal observation than the DS categorization, is feasible in early intervention studies, and can be achieved by using assessment instruments such as the PANSS, SANS, BNSS, or CAINS, which are largely available and do not require an ad hoc training, as the SDS does. Therefore, the persistent negative symptom construct, compared to the DS one, represents a clear improvement in the definition of the target population for clinical trials focusing on negative symptoms. However, efforts are needed to make the definition of persistent negative symptoms consistent across studies. In particular, the definition seems to lack the standardization of thresholds of possible confounding factors (i.e., positive symptoms, depression, and extrapyramidal symptoms). Furthermore, the persistence may vary and is sometimes assessed prospectively, some others retrospectively. According to expert recommendation, clinical trials for negative symptoms should include clinically stable patients in the residual phase of their illness, with negative symptoms that persist despite an adequate antipsychotic treatment for a period of 4–6 months, as ascertained retrospectively and also confirmed prospectively for at least 4 weeks. The prospective evaluation of clinical stability is strongly recommended for negative symptoms, since they are difficult to assess retrospectively for many patients [55].

***Recommendation 1*** (based on studies included in Table e1)

The EPA Guidance Group on Negative Symptoms considers the persistent negative symptom construct suitable for clinical trials based on available evidence. However, the construct has been heterogeneously applied as to the thresholds for depression, positive, and extrapyramidal symptoms. Therefore, the Group suggests the use of thresholds for clinically significant depression (e.g., 6 for Calgary Depression Scale; 17 for Hamilton Depression scale-17 items), for moderate severity of the positive symptoms (e.g., PANSS score ≤ 4) as well as absence of parkinsonism as assessed on validated scales.

**Table:** 

Grade	Recommendation
B	The persistent negative symptom construct should be used in the context of clinical trials. EPA recommends the use of established cutoff scores on validated rating scales for clinically significant depression, moderate positive symptoms, and absence of parkinsonism.

### Factor structures of negative symptom domains

Factor analytic studies on general psychopathological rating scales, such as the PANSS or SANS and the Scale for the Assessment of Positive Symptoms or BPRS, identified items clustering in one or more negative symptom factor/s (Table e2). These studies identified items that do not cluster in the negative symptom factor/s, and provided evidence for excluding attentional impairment (SANS global rating of attention), inappropriate affect (SANS item 6), poverty of content of speech (SANS item 10), difficulty in abstract thinking (PANSS item N5), stereotyped thinking (PANSS item N7), mannerism and posturing (PANSS item G5; BPRS item 24), poor attention (PANSS item G11), and conceptual disorganization (PANSS item P2; BPRS item 15) from the negative symptom dimension (Table e2). Loadings of the items motor retardation (PANSS item G7; BPRS item 18), avolition (PANSS item G13), and active social avoidance (PANSS item G16) have been inconsistent (Table e2).

Based on the consensus initiative and on different factor analytic studies (Table e2) showing the inconsistent loadings of the items N5, N7, P2, G5, G7, G11, G13, and G16 (PANSS), items 6, 10, and the global rating of attention from SANS, as well as items 15, 18, and 24 (BPRS), these symptoms should not be included as negative symptoms in any summary score or subscale score of the negative dimension.

***Recommendation 2*** (based on studies included in Table e2)

**Table:** 

Grade	Recommendation
B	Based on the available evidence, any summary score or subscale score of the negative dimension should use only core negative symptoms, consistently loading on the negative symptom factor: i.e., for the PANSS, the items “Blunted affect” (N1), “Emotional withdrawal” (N2), “Poor rapport” (N3), “Passive/apathetic social withdrawal” (N4), and “Lack of spontaneity and flow of conversation” (N6); for the SANS the subscales “Affective Flattening or Blunting” (items 1–5, and 7), “Alogia” (items 9, 11–12), “Avolition-Apathy” (items 14–16), “Anhedonia-Asociality” (items 18–21); for the BPRS items “Blunted affect” (item 16) and “Emotional withdrawal” (item 17).

Results of studies comparing different negative symptom models (two-factor, three-factor, four-factor, and five-factor models) are described in the NIMH-MATRICS consensus statement [23], in four reviews [4,14,22, 37], in a commentary [24], and in an expert opinion [5] (Table e3). The two-factor model, including the Experiential factor (avolition, asociality, and anhedonia) and the Expressive factor (blunted affect and alogia), has gained large consensus over the past decade [4,5,14,22–24]. Following the consensus statement on negative symptoms [23], the two-factor model was replicated by two studies using the SANS (excluding the Attention subscale) [56,57] and by three studies using the PANSS [58–60]. However, SANS [56,57] and PANSS [58–60] only consider behavior even for the assessment of the experiential deficits (i.e., anhedonia). In addition, studies using the SANS included items that are not considered negative symptoms, such as inappropriate affect and poverty of content of speech [56,57]. Likewise, studies using the PANSS [58–60] included motor retardation, active social avoidance [58–60], avolition, and mannerism and posturing [58,59], which are not regarded as negative symptoms. Results of studies employing rating scales that assess negative symptoms in line with the consensus statement (SDS, CAINS, and BNSS) supported the two-factor model of negative symptoms [56,61–64,29,30,65,66,27,28,67,68]. Thus, the two-factor model seems to be more robust when items unrelated to negative symptoms are excluded. In addition, replications of the two factors were provided independently of treatment and were cross-culturally validated [4]. The two-factor model has influenced the researchers in studying neurobiological underpinnings that could be targeted by different therapeutic options, with important implications in terms of prognosis and treatment [4]. Although the two-factor model has been widely validated and is more robust when negative symptoms are assessed using second-generation rating scales, such as the BNSS and the CAINS, a three-factor model using the BNSS [69] and a four-factor model using the CAINS [70] were also reported (Table e3).

Recently, a review by Strauss and colleagues (2019) [37], which includes three more recent studies conducted by the same research group, has questioned the validity of the two-factor model [71–73]. The strengths of these studies are the followings: (a) they are multicenter studies with large sample size; (b) two studies [71,72] used the confirmatory factor analysis (CFA); (c) one study [73] performed the network analysis to overcome the CFA limitations, in particular, the underestimation of the number of factors in the presence of high correlations between factors and small sample size; (d) these studies for the first time used CFA or network analyses of negative symptoms assessed with new-generation rating scales such as the BNSS and the CAINS [37]. On the whole, the results of these studies showed that a five-factor model, with five factors reflecting the five domains identified by the NIMH-MATRICS Consensus statement, provided the best fit independently of cultures and languages, while a hierarchical model (five negative symptom domains as first-order factors, and the two factors, Experiential and Expressive factors, as second-order factors) showed a slightly worse fit. The results of these studies [71,72] were also replicated by an independent multicenter study [74]. The two studies [71,73] identified a potential sixth factor, “lack of normal distress” of the BNSS (a reduction in the intensity or frequency of negative emotional experience), that corresponds to the “diminished emotional range” item of the SDS, which also assesses the consummatory anhedonia. However, the results of previous factor analytic studies are controversial. Five SDS studies reported that the item “diminished emotional range” loaded on the Expressive factor [56,61–64]. The BNSS studies found that the item “lack of normal distress” loaded on the Expressive factor, with a low saturation [67] and presented low communalities [27]. Further studies are needed to clarify whether the lack of normal distress belongs to the current negative symptom construct or whether it is part of other psychopathological constructs.

Actually, the above-mentioned studies were conducted by the same investigators [37,71–73], thus requiring independent validation; in addition, the psychometric properties of the rating scales considered in these studies (BNSS and CAINS) do not allow an adequate testing of the model, since a factor with less than three items (avolition and asociality include only two items) is generally considered weak and unstable [75]. Notwithstanding the importance of findings provided by CFA and network analyses for future investigations on negative symptom structure and pathophysiological underpinnings, as well as for treatment trials, so far, the available evidence is not strong enough for recommending the use of the five-factor model in clinical trials.

No recommendation is deemed appropriate by the EPA Guidance Group on Negative Symptoms on the factor model to be used in clinical trials. However, as most CFA equally supported the five-factor and hierarchical models of negative symptoms, in which second-order factors were the Experiential and Expressive ones, EPA considers potentially useful to report treatment effects separately for these two factors, which include more than three items and are psychometrically stronger than the five individual domains for all second-generation rating scales as well as SANS, but not PANSS-Negative, BPRS, and the Negative Symptom Assessment (NSA) Scale.

### The burden of negative symptoms in schizophrenia

Negative symptoms pose a substantial burden on patients with schizophrenia, their families, and society. In fact, negative symptoms are related to poor functional outcome, increased unemployment, greater severity of the illness, and usually higher antipsychotic dosages [7,76–78]. A substantial literature, nicely summarized in Awad and Voruganti, highlighted the burden of care [79]. The burden of care is a complex construct encompassing the impact and consequences of the illness on caregivers. Usually, it is subdivided into a so-called “objective burden of care”, which indicates the effect of the disease on taking care of daily tasks (e.g., the household tasks), whereas the so-called “subjective burden of care” indicates the extent to which the caregivers perceive the burden of care [79]. If symptoms persist over a longer period, as could be shown in 25–30% of the patients [80], this patient group will show impaired personal and social functioning, unsuitability for work, and reduced quality of life, which include problems with mobility, washing, and dressing. In parallel, this study looked at the carer burden and found that carers of this specific group of patients do devote an average of 20.5 h per week with a notable negative impact on the quality of life measures to support ill relatives [80].

In general, increased symptomatology is connected to an increased family burden [81]. Looking at the objective caregiver burden more specifically, the perceived severity of negative symptoms seems to have a direct impact, which is not true for positive symptoms [82]. In families of subjects with schizophrenia the “objective burden” was related to the severity of psychopathology and cognitive deficits, with negative symptoms accounting for the largest percentage of explained variance, while the “subjective burden” was related to psychotic symptoms and age of disease onset, with the latter variable explaining most of the variance [83].

A large-scale study found that the severity of psychopathology in patients, the ability of relatives to cope, and the extent of contacts between patients and relatives were predictive of family burden [84]. Family burden was closely related to patient’s needs and particularly to negative symptoms causing greater disability. A regression model indicated that needs around daytime activities, alcohol and drug consumption, severity of psychotic symptoms, negative symptoms, and degree of disability are all related to higher levels of family burden [85].

While these results indicated a central role of negative symptoms in determining caregiver burden, the majority of studies investigating family burden in schizophrenia did not evaluate them or used only a limited assessment of these symptoms. Thus, further studies are needed to draw conclusions.

## Assessment of Negative Symptoms

### Assessment instruments

Standardized assessments for negative symptoms are necessary in both clinical practice and research. In clinical practice, they allow us to quantify the intensity of the symptoms but especially to appreciate their evolution with a more objective approach. In research, they are essential in therapeutic trials because they provide a standard framework for the definition and quantification of symptoms and allow different clinicians from different cultures to evaluate symptoms of interest in a similar way.

There are two types of scales, on one hand those that have been developed in order to assess symptoms in patients with schizophrenia and on the other hand, those developed for the assessment in other disorders and focused on one domain of the negative symptoms such as apathy/avolition or anhedonia. We can also distinguish scales in which the assessment is carried out by professionals via an interview (hetero-evaluations) and those based on self-evaluations by the patients themselves.

#### Scales developed for assessing symptoms in subjects with schizophrenia

The NIMH-Negative Symptom Consensus Development Conference [23] has been a milestone for the development of second-generation scales covering five negative symptom dimensions (alogia, social withdrawal, anhedonia, blunted affect, and avolition). Consequently, this paper will present the scales developed before (first generation) and after (second generation) this conference.

Seventeen instruments have been identified (Table e4) but only the second-generation scales are detailed in Table e5. Most of these scales are based on observer ratings and aim to quantify the severity of negative symptoms. Recently, self-report scales have been developed, allowing patient self-assessment of their feelings and experience related to negative symptoms.

##### First-generation scales

###### Brief Psychiatric Rating Scale and Positive and Negative Syndrome Scale

Even if BPRS and PANSS are scales covering all the symptoms of schizophrenia, they deserve to be reported for their widespread use in past and present trials. The BPRS is a general psychopathology scale, which originally included 16 items and was later extended to include 18 or 24 items, with ratings ranging from 0 to 6 (or from 1 to 7 depending on the version). Four BPRS negative symptom subscales have been proposed [86], based on factor analyses, but the most widely used is the “anergy” factor including three items, emotional withdrawal, motor slowing, and emotional blunting [87,88]. The sensitivity of this factor to change is lesser than the SANS [89]. Moreover, the negative subscale compared to other subscales presents the lowest inter-rater agreement [90] and insufficient internal consistency [91]. Widely used in therapeutic trials, BPRS as a whole has been supplanted by PANSS since the 1990s.

The PANSS [26] includes 30 items rated from 1 (no symptom) to 7 (severe symptom) with three subscales: positive (7 items), negative (7 items), and general psychopathology (16 items). Each item is scored on a seven-point scale, ranging from 1 to 7. The absence of a zero score implies that computations of ratios (e.g., percent changes) are not mathematically appropriate and might result in an underestimation of a response. A suggested correction is to subtract the minimum score (e.g., 30) from the total score [92]. The negative symptoms subscale (PANSS negative) includes N1 blunted affect, N2 emotional withdrawal, N3 poor rapport, N4 passive/apathetic social withdrawal, N5 difficulty in abstract thinking, N6 lack of spontaneity and flow of conversation, and N7 stereotyped thinking [93]. PANSS has good psychometric validity [94–100] and is still widely used in therapeutic trials, including those that target negative symptomatology (see related paragraph). The existence of a semi-structured interview (Structured Clinical Interview for the Positive and Negative Syndrome Scale for schizophrenia [SCI-PANSS]) and a precise definition of the items and their quantification allow obtaining a very good inter-rater reliability. Internal consistency and test–retest reliability can be considered moderate for the negative subscale. However, compared to other scales (e.g., SANS), PANSS negative subscale had the greatest internal consistency [101] and the use of the SCI-PANSS increases its inter-rater reliability [102,103]. Some limitations must also be underlined. Among the seven negative items, N7 is related to disorganization of thought and N5 to cognitive symptoms. Other limitations of the PANSS are the poor assessment of avolition–apathy, the lack of assessment of anhedonia, and the assessments only based on behavioral observation [4,104–107].

A five-factor model of the PANSS has been developed [108] and among these factors, a negative symptom factor score (NSFS) containing five items from the PANSS negative (N1, N2, N3, N4, and N6) and two items from the general subscale (G7 motor retardation and G16 active social avoidance) has been identified [109]. Evidence for reliability and validity and sensitivity to change of the NSFS in schizophrenia patients with prominent negative symptoms has been demonstrated in one study [110] in which, however, subjects were included if they had either prominent negative symptoms or thought disorganization. Besides the limitations previously suggested, motor retardation and active social avoidance should not be considered as negative symptoms since they might be more related to extrapyramidal symptoms, depression, suspiciousness, or social anxiety. Finally, no single negative symptom factor from PANSS has achieved broad consensus, neither NSFS, even if it has been widely used in many trials, nor the most replicated negative factor including N2, N3, N4, N6, and G7 [111–113].

###### Scale for the Assessment of Negative Symptoms

SANS [25] is an extension of the emotional blunting scale [114] and includes 25 items grouped into the five dimensions: alogia, emotional blunting, avolition–apathy, anhedonia–asociality, and deficit of attention. Each item is defined in a glossary and is scored from 0 to 5. Each of the five dimensions has a global score and a composite score that is the sum of the dimension item scores. The reliability and validity of SANS have been widely proved [98,101,115–118]. However, obtaining corroborative history from a family member may substantially improve the validity of the assessment of negative symptoms [119]. SANS has been translated into several languages. A short SANS version with 11 items and 3 response options has been suggested with similar reliability as the original version [120].

Although SANS is probably the reference in the evaluation of negative symptoms, some weakness has been pointed out [4,72,104–107,121]. Indeed, several factor analyses have supported that the item “deficit of attention” loads on a cognitive factor and other items (“speech content poverty”, “response latency”, and “inappropriate affect”) load more on a disorganization component than on negative factors [122,123]. These results are in accordance with previous data that inappropriate affect, inattention, and blocking should not be considered as negative symptoms [124–126]. In the same vein, the items “poor eye contact” and “grooming and hygiene” did not load on negative dimensions [127]. Moreover, anhedonia and social withdrawal are also criticized for evaluating the observed behavior without taking into account the environment and the desire to establish social relations and the ability to experience pleasure during activities. Furthermore, the fact that both these latter aspects are assessed within the same domain constitutes a further limitation as SANS does not separately assess the five negative domains required by the NIMH-Negative Symptom Consensus Development Conference.

As for the PANSS, the SANS is based on behavior manifested by the patient, leading to substantial overlap with functioning, and poor discrimination of secondary negative symptoms [4]. Moreover, both scales include items, such as “abstract thinking” for PANSS and “attention” for SANS, which rate cognitive deficits, accounting for the association between negative symptoms and cognition [128].

***Recommendation 3*** (based on studies included in Tables e2 and e5)

The EPA Guidance Group on Negative Symptoms considers appropriate the use of a second-generation rating scale to assess negative symptoms in clinical practice and trials. However, due to the present regulatory agency requirements and to the need of further evidence concerning the sensitivity to change of second-generation rating scales for negative symptoms, EPA recommends using a second-generation scale to complement the PANSS and SANS for the assessment of negative symptoms in clinical trials.

**Table:** 

Grade	Recommendation
B	Due to the limits of PANSS negative subscale and SANS according to the present conceptualization of negative symptoms, these scales should be complemented with a second-generation scale in clinical trials.

###### Schedule for Deficit Syndrome

The SDS [42] is the only scale that categorizes patients into deficit and non-deficit subtypes. Six negative symptoms are assessed from 0 (normal) to 4 (severely impaired) in a semi-structured interview: restricted affect, diminished emotional range, poverty of speech, curbing of interests, diminished sense of purpose, and diminished social drive. Deficit schizophrenia is defined by the presence of two or more negative symptoms with a score ≥2 (moderate) and judged both primary (i.e., not caused by neuroleptic akinesia, depression, anxiety, delirium, disorganization, environmental deprivation, and other factors) and enduring for 12 months, including periods of clinical stability and remission of psychotic symptoms. This scale has strong inter-rater reliability and convergent validity [129], and has the greatest stability compared to other scales [130]. However, this scale is difficult to use in clinical practice, and the assessment of persistent negative symptoms is more convenient for clinical trials [44].

While the limitations of the SDS are relevant to the use of the scale to assess negative symptom domains, they should not put into question the validity of the scale to diagnose the deficit syndrome, which remains a validated categorical approach to identify subjects with primary enduring negative symptoms [38].

###### The Negative Symptom Assessment Scale

The NSA [131], largely used in therapeutic trials, is a 16-item scale with a semi-structured interview filled in 30 min, each item is rated on a six-point scale (1–6; or rated as 9 = not ratable). A total score and a global rating are provided. NSA includes five factors, communication, emotion/affect, social involvement, motivation, and retardation. Negative symptoms assessed with NSA-16 drove the changes in the Social and Occupational Functioning Scale rather than the reverse, suggesting that improving negative symptoms may lead to improvements in functional outcomes [132]. However, the ratings for some of the items are based on behavior and thus a substantial overlap with functioning cannot be excluded. The agreement among raters after training was good [133] or among raters coming from different countries was at least as high using the NSA-16 as using the PANSS negative subscale or Marder negative factor [134]. NSA-16 has good psychometric properties and a cutoff point of 31 provided excellent sensitivity and good specificity for separating patients with and without negative symptoms [135].

A short version, which allows rapid evaluation of negative symptoms, exists in the form of a four-item scale (NSA-4; 1. Restricted speech quantity, 2. Emotion: Reduced range, 3. Reduced social drive, and 4. Reduced interests). It was tested by more than 400 medical professionals [136] and presented good psychometric properties [137]. However, the validation of the short version scale has been carried out only by the group developing NSA and should be independently replicated.

The originality of NSA-16 is to evaluate on the one hand the emotional feeling and on the other hand the emotional expression by asking the patient to mimic emotions. However, similar limitations as those evoked with SANS and PANSS can be pointed out [104–107]. Anhedonia is not evaluated as a separate domain since the capacity to feel pleasure during activity is included in the item “emotion: reduced range” also encompassing the capacity to feel anxious or depressed. Consequently, NSA-16 does not cover the five negative domains required. Some items as impoverished speech content, inarticulate speech, and slowed movements are not considered as negative symptoms. Several items (poor grooming and hygiene, reduced hobbies and interest, and reduced daily activity) are based on functioning or behaviors and their severity is measured by considering the type and the frequency of behavior. Scores on NSA, SANS, and SDS may be reliably converted between them [138].

***Recommendation 4*** (based on studies included in Table e5)

The EPA Guidance Group on Negative Symptoms considers appropriate the use of a second-generation scale to assess negative symptoms in clinical practice and trials. As reported for the other first-generation scales, the group recommends using a second-generation scale to complement the NSA-16 for the assessment of negative symptoms in clinical trials.

**Table:** 

Grade	Recommendation
B	Due to the limits of NSA-16 according to the present conceptualization of negative symptoms, this scale should be complemented with a second-generation scale.

##### Second Generation Scales

###### The Brief Negative Aymptom Scale

The BNSS [28] includes a semi-structured interview to evaluate 13 items that measure the five negative dimensions and the lack of distress. According to the authors of the scale, the interview requires 10–15 min; however, in practice, it generally takes longer (20–25 min). The scale presents good psychometric properties (Table e5). Several studies reported that negative symptoms measured with the BNSS are not significantly affected by the presence of depressive or positive symptoms in stable schizophrenia patients [27,139,140].

BNSS originality is to take into account the expression of internal experiences and the observed behavior for the social withdrawal and avolition dimensions. Anhedonia is also evaluated by differentiating the consummatory and anticipatory pleasures. An item evaluates the ability to feel distress, and the lack of “distress” is considered as pathological. This item is the subject of controversy, some authors considering that it is not consistent with the definition of negative symptoms [105], others supporting that might help to differentiate primary and enduring symptoms from secondary negative symptoms [140]. BNSS was designed for easy application in the context of clinical trials or clinical routines and has excellent psychometric properties in schizophrenia [28,113] and in bipolar disorders (76). It has been translated and validated into 29 languages [141], notably Danish [142], Polish [143], German [144], Brazilian [68,145], and Spanish [146]. Nine translations were used in a European validation study [74]. BNSS has substantial advantages with respect to PANSS for the identification of the experiential domain (including avolition, asociality, and anhedonia) and in subjects with predominant negative symptoms [74]. Preliminary evidence indicates that BNSS is also sensitive to change [147].

###### The Clinical Assessment Interview for Negative Symptoms

The CAINS came from the Collaboration to Advance Negative Symptom Assessment in Schizophrenia [104]. The development of CAINS was based on data-driven iterative process leading to several successive versions [29,30,148]. In its final version, the scale includes 13 items and is administered in 15–30 min, each item being scored on a five-point Likert scale. As BNSS, CAINS contains a comprehensive manual and workbook that provides a semi-structured interview. CAINS addresses the notions of anticipated and consumed pleasures, motivation through the social, professional, and leisure domains. Goal-oriented behaviors are evaluated through the patient’s effort to engage in an activity. The scale has good psychometric qualities and several factor analyses displayed two factors, MAP and EXP (Table e5). These two subscales have good psychometric properties and have been validated in a large sample from nonacademic clinical settings by raters not affiliated with the scale’s developers [149]. A proxy scores of >25 on the CAINS total or a proxy score of >17 on the MAP has been proposed to identify subjects with persistent negative symptoms [150]. These data need to be replicated by an independent group.

CAINS is available in several languages such as Czech, French, Spanish, Mandarin, Cantonese, Korean, Polish, Greek, Swedish, Lithuanian, and German [105]. Validation studies of CAINS translated into Chinese [151,152], Korean [153,154], Spanish [155], and German [65] have been published.

As BNSS, CAINS is based on observer rating and does not need informant to be completed. Both scales assess behavior for the five negative dimensions and internal experiences for avolition and social withdrawal. However, if BNSS contains distinct items for assessing internal experiences, CAINS combines internal experiences and observed behaviors in the same ratings. As BNSS, CAINS yields scores reflecting MAP and EXP. A direct psychometric comparison of BNSS and CAINS showed high correspondence for blunted affect and alogia items but moderate convergence for avolition and asociality items, and low convergence among anhedonia items [156]. This finding on anhedonia may be related with the different definitions of items and how these items on anhedonia are assessed. Indeed, CAINS examines the frequency of pleasure and has distinct items assessing social, work, and recreational pleasures while BNSS assesses frequency and intensity of pleasure and has one item assessing social, work, and recreational pleasures, and physical pleasure.

***Recommendation 5*** (based on studies included in Tables e3 and e5)

EPA considers the use of the BNSS or CAINS appropriate to assess negative symptoms in clinical practice and trials as these scales provide an adequate assessment of all negative symptoms domains (Evidence level I–II). As the evidence concerning their sensitivity to change is limited for BNSS and not present for CAINS, EPA recommends using these scales to complement first-generation scales (such as PANSS, SANS, or NSA-16) in clinical trials.

**Table:** 

Grade	Recommendation
B	Due to their good psychometric properties and coverage of the five domains of negative symptoms, BNSS or CAINS should be used for the assessment of negative symptoms. In clinical trials, they should be used to complement first-generation scales.

##### Scales based on self-assessments

Self-assessments should be considered as complementary measures of scales based on observer-ratings. Compared to these last evaluations, self-evaluation provides clinical information not necessarily detected by caregivers or medical staff in a standard interview and can provide information on the symptoms as recognized by the patients themselves [157].

Two recent scales, the Motivation and Pleasure Scale Self-Report (MAP-SR) [158] and the Self-evaluation of Negative Symptoms (SNS) [159] have been developed specifically for the negative symptoms and supplanted previous tools that do not have good psychometric properties or do not cover the five negative dimensions required [160–163].

###### The Motivation and Pleasure Scale-Self-Report

The MAP-SR [158] is a self-assessment scale derived from the CAINS motivation/pleasure subscale. The Expression items were removed due to poor reliability and validity, yielding a 18-item version of the MAP-SR [164]. This point might be considered as a weakness since emotional expression or emotional feeling might allow to differentiate between negative and depressive symptoms [159,165]. Although the 18-item version demonstrated adequate internal consistency, three items were excluded due to low item-total correlations yielding a 15-item version. Anhedonia is assessed with six items focusing on experienced and expected pleasure in social, physical, and recreational/vocational domains. Asociality and avolition are evaluated with three and six items, respectively, each item scoring from 0 to 4. This scale presents good psychometric properties [158] and has been translated and validated into German [166] and Korean [167]. However, it only focuses on the motivation/pleasure dimension and if it is adequate to assess anhedonia it might be less suitable when assessing motivation [168]. Moreover, the evaluation contains many questions like “how often” and “how much”, which require that patients remember and quantify what feelings or events happened in the past week, potentially difficult for patients with memory impairment.

###### The Self-evaluation of Negative Symptoms

The SNS [159,169] is a concise and easy-to-understand self-assessment scale consisting of 20 items, most of which coming from verbatim reports of patients with schizophrenia. The patient has three choices of answers “completely agree”, “slightly agree”, “strongly disagree” corresponding to 2, 1, and 0, respectively. Thus, a total score (from 0 to 40 for severe negative symptoms) and five sub-scores can be obtained. The advantage of this scale is also to take into account the consummatory and anticipatory pleasure. A pathological threshold at 7 was determined with a very good sensitivity and specificity in patients with schizophrenia and schizoaffective disorders compared to healthy subjects [170]. SNS was also used in a general adolescent population demonstrating its possible use for the screening of negative symptoms [171]. This scale was translated into more than 17 languages [172].

***Recommendation 6*** (based on studies included in Table e5)

**Table:** 

Grade	Recommendation
C	Self-assessments can be used to complement observer-ratings. SNS (exploring five domains) and MAP-SR (exploring three domains) can be used for self-assessment of negative symptoms.

#### Scales focused on one dimension of negative symptoms

Even if negative symptoms are considered as core features in patients with psychotic disorders, they are not specific to schizophrenia and can be found in other mental or neurological disorders such as depression, parkinsonism, dementia, and even in the general population. Consequently, some scales assessing, in particular, anhedonia and avolition/apathy were initially developed in disorders other than schizophrenia. Only scales that were validated in patients with schizophrenia and that presented good psychometric properties are displayed in Table e6.

The scales assessing anhedonia need more validation studies in schizophrenia to be recommended for the assessment of this domain of negative symptoms.

Three kinds of measures have been used in assessing motivation deficit or apathy in schizophrenia, self-reported, clinician-rated, and performance-based measures.

The Apathy Evaluation Scale (AES), commonly used in neurological disorders [173], has been also validated in schizophrenia [174]. The scale comprises 18 core items that assess and quantify the affective, behavioral, and cognitive domains of apathy but with phrasing varying by rater [self, informant, or clinician] and that rates on a four-point response scale (0 = not at all true/characteristic to 3 = very much true/characteristic). The clinician version of the AES was also validated in first psychotic episode [175]. The scores of AES, SANS, and Quality of Life Scale (QLS) were highly intercorrelated, supporting that these instruments evaluating motivational deficits are tapping into a similar underlying construct [176]. A validated shortened Self-reported Apathy Evaluation Scale was also validated in first psychotic episode [177]. It is a 12-item scale, each item scoring on a four-point Likert scale, higher scores indicating severe apathy. The questions focus on the degree of self-experienced motivation and interests during the last 4 weeks and do not include measures of functioning.

***Recommendation 7*** (based on studies included in Table e6)

**Table:** 

Grade	Recommendation
D	The Apathy Evaluation Scale (AES) could be regarded as a useful tool for the assessment of apathy in schizophrenia.

### Assessment of negative symptoms in first-episode psychosis patients

In first episode psychoses, the assessment of negative symptoms is of interest for several reasons. Meta-analyses on first episode studies find that a higher level of negative symptoms is associated with a lower quality of life [178] and is predictive of a poorer functional outcome in terms of functional recovery [179]. Likewise, first-episode psychosis patients with a high level of negative symptoms have a lower adherence to treatment [180] and an increased risk of deliberated self-harm after treatment [181].

In the above-mentioned meta-analyses, most of the included trials used the original seven-item sub-score PANSS-Negative to estimate the severity of negative symptoms, while a minority of them measured negative symptoms with the SANS scale. The second-generation scales, that is, BNSS and CAINS, were not used in any of the included trials and there are no published first episode studies using them. Validation studies were mainly carried out in stable and/or chronic patients. Only one study, published after the search end date, included a small sample of unstable, first episode patients [142] and found a low discriminant validity with respect to positive symptoms and parkinsonism. Although the preliminary nature of these findings does not allow conclusions, they suggest that the challenge of separating primary negative symptoms from those secondary to psychosis and parkinsonism is not yet solved with the use of second-generation scales, such as BNSS, in first episode subjects. Accurate assessment of positive symptoms, depression, and parkinsonism should be carried out in FEP subjects to exclude the secondary nature of negative symptoms.

Although the vast majority of first episode studies have used PANSS or SANS for evaluating negative symptoms, there have been few studies focusing on specific domains, particularly apathy/avolition/amotivation. Only the Apathy Evaluation Scale has been validated in a sample of first episode patients [175] and was used in two studies [182,183].

As to the factor structure of negative symptoms in first episode samples, the sum score of selected items from PANSS believed to cover the subdomain of amotivation [184] have been used in two studies [185,186]. In line with this, few studies have used a suggested factor-structure from the SANS [187] to report on the severity of amotivation [188,189]. Several studies have reported specifically on each of the four SANS-subdomains, that is, Affective flattening, Alogia, Anhedonia/Asociality, and Avolition/Apathy [190–193]. For both scales, confirmatory factor analyses in first episode samples were published in 2013. The Wallwork/Fortgang five-factor model of PANSS [112] was confirmed to have a reasonable fit in patients with first-episode psychosis [194]. The factor-analyses on SANS detected a three-factor model, consisting of expressivity, experiential, and alogia/inattention, which showed similar model fit as the original SANS five-factor model [195]. However, in these factor analyses performed in first episode patients, none of the suggested factor models fully covers the five domains identified by the NIMH-consensus statement. Validation of BNSS and CAINS in first episode samples is therefore crucial for future optimal assessment of negative symptoms in this group of patients.

Because of the convincing prognostic role of negative symptoms in first episode psychosis [178–181], efforts have been made to identify patients with the deficit syndrome or persistent negative symptoms early in the disease. Identifying the deficit syndrome already at the time of admittance to psychiatric services is challenged by the inclusion of a 12-month observation period in the original criteria [31] and the need to use the specific scale, SDS [42]. When SDS is combined with a longitudinal observation-period, only 5–10% of a first episode cohort fulfill the criteria for the deficit syndrome [196], whereas 37% of the patients from another cohort was identified when SDS was applied without a longitudinal observation period [197]. When using proxy-measures based on BPRS or PANSS [45] in first episode studies, 26–31% fulfill the criteria of deficit syndrome [198,199], but again, these high numbers were based on cross-sectional observations only.

In order to evaluate the number of first episode patients with persistent negative symptoms, comparisons of six different definitions were carried out; the proportion of patients with persistent negative symptoms varied between 11 and 26 % [200]. This is in contrast to a large European first episode cohort, where only 6.7% of the sample was identified to fulfill the criteria for persistent negative symptoms when controlling for confounders like depression and parkinsonism [201].

In conclusion, most of the available studies in the literature on negative symptoms in first episode patients are based on measures from the first-generation negative symptom scales, mainly using the original factor-models of PANSS or SANS. Although new factor-models of PANSS and SANS were validated in first episode patients, they have not really gained a large diffusion in first episode studies, and they still have the shortcoming that they do not cover all five negative symptom domains. In contrast, both BNSS and CAINS cover all five domains, but neither of them has been validated nor implemented in first episode studies. Therefore, more experience with these scales in first episode samples is needed. Moreover, agreements on how to integrate the second-generation rating scales in the definitions of “the deficit syndrome” and “persistent negative symptoms” and control for confounding effect of secondary negative symptoms in first episode studies are warranted.

### Assessment of negative symptoms in clinical high-risk individuals

As the assessment and treatment of attenuated psychotic symptoms have traditionally been the primary focus in CHR settings [202,203], less attention has been given to the assessment of negative symptoms. The pivotal role of negative symptoms in CHR states is, however, reflected in findings of negative symptoms preceding the emergence of attenuated psychotic symptoms [204], and studies reporting negative symptoms of an equal magnitude in CHR individuals and patients with a first-episode psychosis [205,206]. Additionally, persistent negative symptoms of a moderate to high severity level are present in a subgroup of CHR individuals [204,207]. Abundant evidence shows negative symptoms to be robustly associated with profound functional impairments in CHR individuals [208–216] as well as a predictor of transition to psychosis [204,207,211,217]. This key role of negative symptoms in CHR states is also recognized in the proposal to include negative symptoms to define and enroll CHR samples [218].

While the rationale for evaluating negative symptoms in CHR states is robust, the assessment of negative symptoms in early intervention settings is commonly conducted by employing scales developed for the adult psychosis population (the SANS and the PANSS), or by using scales developed primarily for the assessment of attenuated psychotic symptoms with only aspects of negative symptoms being captured the Structured Interview for Prodromal Symptoms (SIPS) [219] and the Comprehensive Assessment of At-Risk Mental States (CAARMS) [220]. Reviewing the literature on predominantly larger-scale intervention trials in the CHR population assessing negative symptoms revealed the SIPS negative (*N* = 9) and the PANSS-Negative (*N* = 6) to be the most commonly used measurements followed by the SANS (*N* = 4) and CAARMS negative (*N* = 3) (depicted in Table e7). The vast majority of studies used the total scores of the instruments with only two studies using subscale scores (from the SANS). No intervention trial could be retrieved that used a second-generation negative symptom scale. While being frequently used scales, the PANSS, SANS, SIPS negative, and CAARMS negative have conceptual and psychometric limitations precluding an accurate understanding of the negative symptom complex in CHR states. We have already reviewed the psychometric limitations of the PANSS and SANS. Furthermore, these instruments have been developed for use in adult patients with manifest psychosis and may therefore not be sensitive to the potentially more subtle negative symptoms occurring in adolescents and young adults that constitute the CHR population. The SIPS and the CAARMS negative item scales, while being instruments developed specifically for the CHR population, do suffer limitations such as a significant content overlap between negative symptoms and functioning [209] and importantly, the scales do not assess the five domains of negative symptoms [23] and are therefore not in line with the present conceptualization of negative symptoms. In order to meet the advanced understanding of the negative symptom complex, it is advisable that the assessment of negative symptoms in CHR samples is conducted using second-generation negative symptom scales that have addressed the shortcomings of the previous scales. However, the two scales developed after the MATRICS Consensus initiative on negative symptoms, the BNSS and the CAINS were developed for primary use in adult samples with established psychotic disorders. To meet the requirements of scales used in CHR populations, adapted versions of the BNSS and the CAINS have been developed [221,222]. The adaptations to the scales comprised revising the probes so that they were relevant to the lifestyle and activities of adolescents and young adults (e.g., leisure activities or living situation), but the item anchors were in keeping with the original versions. In a study of 29 CHR participants, the BNSS adapted version showed strong internal consistency, good inter-rater reliability (0.85), and discriminant and convergent validity [221]. Similarly, the CAINS adapted version was administered to 29 CHR individuals, 31 patients with schizophrenia, and 32 healthy controls, revealing the CAINS to distinguish CHR from healthy controls with moderate to large effect sizes. Furthermore, the study established concurrent validity of the CAINS in a CHR sample [222]. While these studies provide preliminary evidence for the utility of the BNSS and the CAINS in CHR samples, future longitudinal studies are needed to elucidate on the stability of the BNSS and CAINS measurements in CHR samples. Finally, the Prodromal Inventory of Negative symptoms (PINS) is a second-generation negative symptom measure developed specifically for use in the CHR population [223]. In a study of 53 CHR individuals, the PINS showed good inter-rater reliability (>0.80), internal consistency, and convergent validity. By conducting 12 months follow-up assessments, the PINS proved to have high temporal stability on two PINS items, although the finding on the stability of the total score is equivocal [223].

A common feature of the BNSS, CAINS, and PINS is that they produce positively skewed data in CHR samples, indicating that, even though the scales have been developed to detect the subtleties of negative symptoms in CHR states, they may not be capturing the phenomenology of negative symptoms at the lower end of the spectrum. This warrants a further refinement of these scales, or the development of new scales that may be sensitive to the attenuated negative symptoms occurring in CHR states. In conclusion, the results on the use of the second-generation negative symptom scales in CHR populations are promising, but still in the initial stages with recognized limitations of the available measures. Despite these limitations, the PINS and the modified versions of the BNSS and the CAINS are currently the best available measures of negative symptoms in CHR populations, as they overcome the limitations of previous scales and are adapted (BNSS and CAINS for youth) or developed (PINS) to be used in CHR subjects.

Priority should, however, be given to future development of negative symptom scales with extended item selection mapping the breadth of negative symptoms in CHR states along with maintaining robust psychometric properties.

### Differentiating primary and secondary negative symptoms in the clinical practice

Negative symptoms are etiologically heterogeneous and may be mimicked and/or exacerbated by a variety of factors, often present in schizophrenia. Examples include blunted affect or avolition secondary to antipsychotic-induced akinesia and amotivation (especially with first-generation antipsychotics), social withdrawal due to delusions of moderate severity (e.g., delusions of persecution or reference with an impact on behavior), anhedonia due to depression, or avolition in chronic institutionalized subjects [4,5]. The correct identification of negative symptoms and the differentiation between primary and secondary negative symptoms is crucial in the clinical practice since it has diagnostic, prognostic, and therapeutic implications. Some of the factors causing secondary negative symptoms, for example, positive symptoms, depression, or extrapyramidal side effects, can be treated or reduced and result in improvement of the functional outcome and quality of life of the affected subjects. However, to date, there is limited evidence on the best methods for differential diagnosis (i.e., distinguishing primary vs secondary negative symptoms) in clinical practice.

The distinction between primary and secondary negative symptoms has been made with high inter- and intra-rater reliability and accuracy in research settings [38]. However, in clinical settings, without highly specialized training on specific research instruments, such as the SDS, or the availability of extensive longitudinal information on possible factors causing secondary negative symptoms in each patient, the distinction can be made with modest inter- and intra-rater reliability as reported by the only available study [224].

No further study has investigated the feasibility and reliability of the distinction in clinical practice. However, two expert opinion papers [33,41], a narrative review [35], and a systematic review [4] are available and provide some clarifications on how to distinguish between primary and secondary negative symptoms (Table e8).

Data concerning covariation of negative, psychotic, and extrapyramidal symptoms can be also extrapolated from clinical and pharmacological trial studies (Table e8). Secondary negative symptoms can sometimes be recognized based on “ex adiuvantibus” criteria, that is, their response to specific therapeutic interventions [33,35].

An algorithm was developed and recently revised and extended in order to assist clinicians in classifying negative symptoms as primary or secondary [33,35,41]. The algorithm does not provide criteria for differential diagnosis, but a guide to support the clinical judgment. Both the original algorithm and the revised one mainly consider the course of negative symptoms: those with episodic appearance, temporally related to potential confounding factors (such as recent increase in drug dosage or acute psychotic exacerbation), which improve with the correction of the confounders, are more likely secondary negative symptoms.

It is worth noticing that recognition of secondary negative symptoms, according to these algorithms, requires either a prospective repeated examination of subjects with schizophrenia on antipsychotic treatment, or the availability of adequate information.

The possibility to recognize secondary negative symptoms in first-episode subjects often requires a prospective longitudinal observation as extensive retrospective information is not always available.

The present review will summarize all available evidence on identification of secondary negative symptoms that have not improved or had appeared or worsened over time in subjects with a diagnosis of schizophrenia treated according to the available guidelines.

#### Recognition of secondary negative symptoms due to positive symptoms

In most cases, these negative symptoms demonstrate concurrent improvement with positive symptoms during antipsychotic treatment and concurrent worsening during periods of psychotic exacerbations or drug washout [4,33,35].

In clinical settings, the recognition of these secondary negative symptoms requires the investigation of patients’ internal experience as well as the course of negative symptoms during periods of psychotic exacerbation, changes in antipsychotic medication, and clinical stability. Negative symptoms are more likely secondary to the positive ones when they get worse with drug withdrawal and/or during psychotic exacerbations. On the contrary, they are more likely primary in the presence of a stable level of severity, independently of clinical stability or medication changes [4,33,35]. A single study (evidence level III), in subjects treated with haloperidol monotherapy for at least 3 months and then undergoing a 6-week washout period, demonstrated that changes in the factor diminished motivation (including asociality, anhedonia, and avolition), in the washout period, were predicted by changes in anxiety/depression and psychosis, while changes in affective flattening were predicted by changes in extrapyramidal side effects. Thus, covariation of positive symptoms or depression with negative symptoms might apply only to some domains of negative symptoms, such as asociality, anhedonia, and avolition.

**Figure 2.**
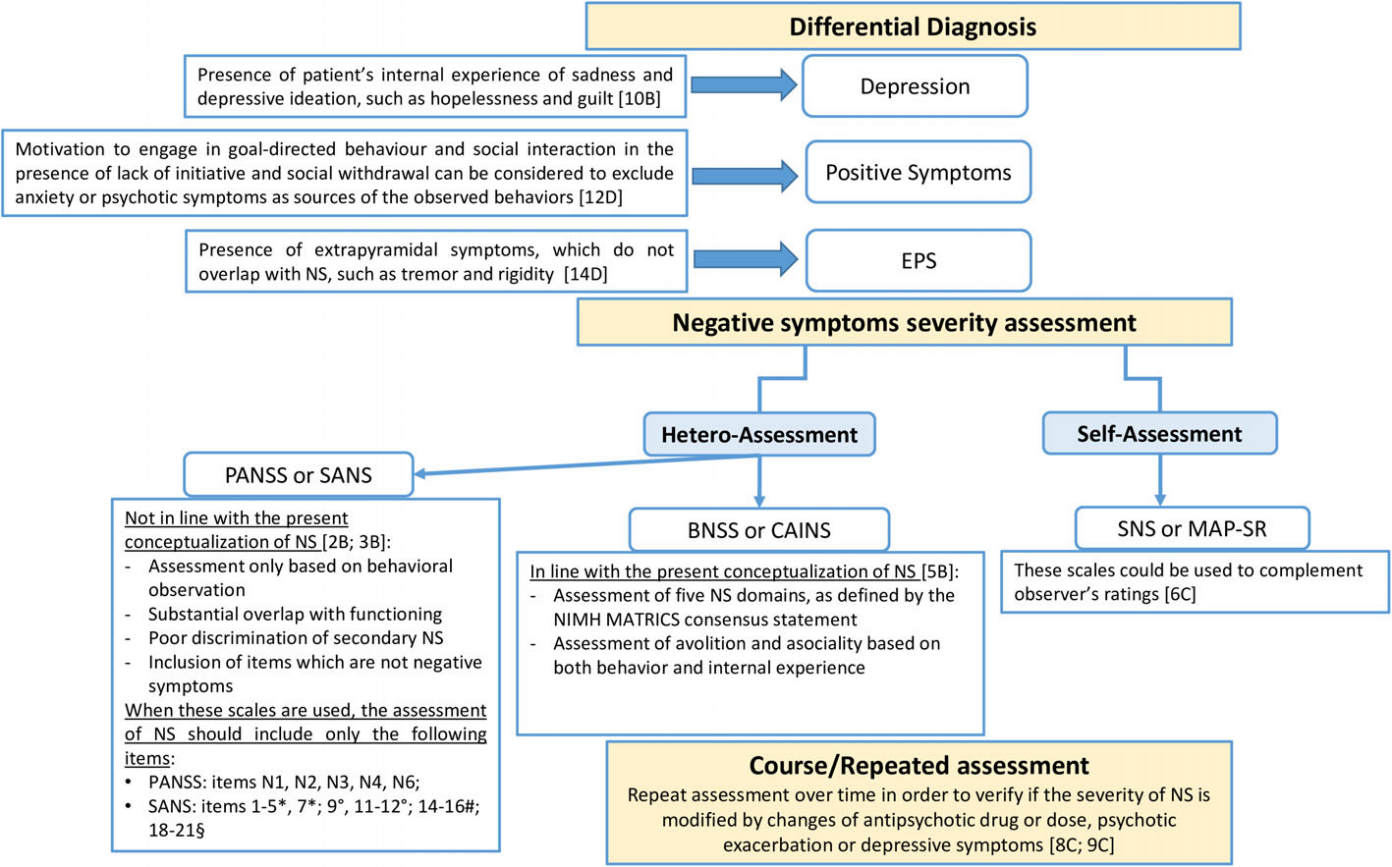
Clinical suspicion of negative symptoms—decision tree NS: negative symptoms; PANSS: Positive and Negative Syndrome Scale; SANS: Scale for the Assessment of Negative Symptoms; BNSS: Brief Negative Symptom Scale; CAINS: Clinical Assessment Interview for Negative Symptoms; SNS: Self-evaluation of Negative Symptoms; MAP-SR: Motivation and Pleasure Scale-Self-Report. The square brackets in the figure report the corresponding number and grade of the recommendations present in the text. PANSS items: N1 = Blunted affect, N2 = Emotional withdrawal, N3 = Poor rapport, N4 = Passive/apathetic social withdrawal, N6 = Lack of spontaneity and flow of conversation; *SANS Affective Flattening or Blunting subscale items: 1 = Unchanging facial expression, 2 = Decreased spontaneous movements, 3 = Paucity of expressive gestures, 4 = Poor eye contact, 5 = Affective nonresponsivity, 7 = Lack of vocal inflections; SANS Alogia subscale items: 9 = Poverty of speech, 11 = Blocking, 12 = Increased Latency of Response; #SANS Avolition-apathy subscale items: 14 = Grooming and Hygiene, 15 = Impersistence at work or school, 16 = Physical anergia; §SANS Anhedonia-Asociality subscale items: 18 = Recreational Interests and Activities, 19 = Sexual interest and activity, 20 = Ability to feel intimacy and closeness, 21 = Relationships with friends and peers.

Based on available evidence, the algorithms suggest to wait the improvement of negative symptoms following effective treatment of positive symptoms. However, for the domains of asociality, anhedonia, and avolition in particular, according to expert opinions and available reviews (Table e8), the investigation of subjects’ internal experience provides important information well before the observation of concurrent improvement of positive symptoms. In particular, clinicians need to assess whether social withdrawal, reduced involvement in pleasurable activities, or avolition are due to distress caused by delusions or other psychotic experiences, anxiety, or concomitant depression (Figure 2). Clinician should further inquire about the degree to which subjects with schizophrenia value and desire close relationships, enjoy available sources of pleasure, or struggle to participate in activities.

#### Recognition of secondary negative symptoms due to side effects

It is very difficult to differentiate between the Expressive Deficit domain of negative symptoms, including blunted affect and alogia, and drug-induced parkinsonism [4,35,225].

To recognize negative symptoms due to antipsychotic drug treatment in the clinical practice, expert opinion papers, available reviews, and proposed algorithms recommend the evaluation of blunted affect and alogia course, taking into account changes in antipsychotic treatment [4,33,35]. In fact, in the case of drug-induced blunted affect and alogia, a linear increase in the severity of the symptoms will be noticed as a consequence of the drug dose increase, and the variation will be even more noticeable if the drug used is a first-generation antipsychotic. In addition, a standard clinical examination to assess the presence of other extrapyramidal signs, such as tremor or rigidity, which are not negative symptoms, should be carried out to exclude or diagnose drug-induced parkinsonism [4,33,35].

In the clinical practice, the distinction between primary and secondary avolition can be challenging, and sedation and/or amotivation induced by antipsychotics, especially first-generation ones, should be considered as part of the assessment [226,227]. Longitudinal observation showing an increased severity with an increase in drug dose or the appearance of the symptom following the introduction of an antipsychotic will support the classification of the symptom as secondary (Figure 2).

#### Recognition of secondary negative symptoms due to depression

The level of evidence for differential diagnosis between primary negative symptoms and negative symptoms due to depression is based on two expert opinions, three narrative reviews, and two systematic reviews [4,33,35,228–230]. It is challenging to distinguish between primary negative symptoms, secondary negative symptoms due to depression, and depression without negative symptoms [4,33,35,41].

Depression is an important co-occurring syndrome in schizophrenia, presenting with substantial anhedonia, reduced goal-directed behavior, and social withdrawal, that is, symptoms that are in overlap with negative symptoms [4,35,228–231]. However, according to a meta-analysis conducted by Lako and colleagues (2012) [232] and three more recent studies [233–235], the differential diagnosis might improve using the Calgary Depression Scale for Schizophrenia [236], which is considered the best assessment instrument for depressive symptoms in subjects with schizophrenia compared to other scales such as the PANSS, the BPRS, the Hamilton Rating Scale for Depression, the Montgomery–Asberg Depression Rating Scale, the Beck Depression Inventory, as well as the Quick Inventory of Depressive Symptomatology-Self-Report (Table e8).

Furthermore, subjects with schizophrenia and those with depression have been found to differ more in self-assessment of depressive symptoms than in observer ratings. Subjects with schizophrenia with negative symptoms self-reported fewer depressive symptoms than those observed by clinicians, unlike subjects with depression [165]. Therefore, the investigation of the subjective feelings of depression might help identifying subjects with depression and instigate appropriate treatment with improvement of the mood disorder and secondary negative symptoms [165]. If we consider the two-factor model of negative symptoms, the relationship is primarily between depression and avolition–apathy [159,229,230], while the Expressive Deficit is more characteristic of negative symptoms [4,159,165].

Therefore, high scores for self-reported depressive symptoms in the presence of unimpaired expressive functions suggest a depressive syndrome [4,165]. According to the reviewed evidence, the presence of the subjective component of depressed mood as well as depressive ideation, such as hopelessness and guilt, favor the diagnosis of depression and should be clinically assessed, whereas the presence of blunted affect is more characteristic of negative symptoms (Figure 2).

#### Recognition of secondary negative symptoms due to substance abuse and social deprivation

Despite the hypothesized relationship between substance abuse and negative symptoms, to date, the impact of comorbid substance abuse on negative symptoms in schizophrenia remains controversial and requires further investigation [35]. Nevertheless, a drug history should be obtained for patients presenting with negative symptoms.

With regard to social deprivation, the evidence regarding the relationship between this factor and negative symptoms is scant [35,237,238]. Based on the improvement of these symptoms after deinstitutionalization, it has been hypothesized that chronic institutionalized patients might present negative symptoms due to a hypostimulating environment [35]. However, it is not clear whether the possible improvement of negative symptoms after discharge is linked to the deinstitutionalization or community programs or both these factors [35,239]. In addition, there is no evidence of the impact of social deprivation in outpatients. Thus, further studies are needed to draw conclusions.

#### Recommendations

Evidence for the differentiation between primary and secondary negative symptoms is limited.

On the basis of the limited evidence available, which can be classified as Level II–IV (Table e8) in most cases, the recommendations for differentiating primary from secondary negative symptoms in clinical settings can only be of grade C or D. The EPA Guidance Group on Negative Symptoms elaborated the following recommendations.

***Recommendation 8*** (based on studies included in Table e8)

**Table:** 

Grade	Recommendation
C	Patients presenting with negative symptoms can be repeatedly assessed over time to identify possible sources of secondary negative symptoms that might be amenable to treatment.

***Recommendation 9*** (based on studies included in Table e8)

**Table:** 

Grade	Recommendation
C	To identify secondary negative symptoms, it can be useful to verify if their severity is modified by changes of antipsychotic drug or dose, or psychotic exacerbation or depressive symptoms over time.

***Recommendation 10*** (based on studies included in Table e8)

**Table:** 

Grade	Recommendation
B	To identify depression as a cause of secondary negative symptoms in subjects with schizophrenia, the Calgary Depression rating Scale should be used to investigate patient’s internal experience of depressed mood and depressive ideation, such as hopelessness and guilt.

***Recommendation 11*** (based on studies included in Table e8)

**Table:** 

Grade	Recommendation
C	The presence of expressive deficits can be more characteristic of subjects with negative symptoms than of those with depression.

***Recommendation 12*** (based on studies included in Table e8)

**Table:** 

Grade	Recommendation
D	Patient’s internal experience of motivation to engage in goal-directed behavior and social interaction in the presence of lack of initiative and social withdrawal could be considered to exclude anxiety or psychotic symptoms as sources of the observed behaviors.

***Recommendation 13*** (based on studies included in Table e8)

**Table:** 

Grade	Recommendation
D	In the presence of negative symptoms and concomitant moderate to severe positive symptoms, remission of positive symptoms could be pursued before classifying negative symptoms as primary.

***Recommendation 14*** (based on studies included in Table e8)

**Table:** 

Grade	Recommendation
D	In subjects with negative symptoms treated with antipsychotics, a standard clinical examination to assess the presence of extrapyramidal signs, which are not in overlap with negative symptoms (e.g., tremor or rigidity), could be carried out to exclude drug-induced parkinsonism.

## Discussion

The definition of negative symptoms has improved in the last decades, and studies reviewed in the present paper provide evidence that they can be reliably assessed using appropriate instruments. In line with the NIMH consensus conference and major systematic reviews [4,5,22,23], the negative symptom dimension includes five domains: blunted affect, alogia, anhedonia, avolition, and asociality.

Signs and symptoms resembling negative symptoms are sometimes due to other illness dimensions, in particular positive symptoms, depression, extrapyramidal symptoms, sedation, environmental deprivation, or substance use. In this case, they are named secondary negative symptoms. The exclusion of factors underlying secondary negative symptoms is important in clinical trials aimed to test efficacy of new treatments for negative symptoms.

The present guidance for the optimal assessment of primary and persistent negative symptoms is based on expert consensus and systematic reviews [4,14,32,34,36,38,39,43,44].

Based on the reviewed evidence, we recommend the use of the persistent negative symptom construct in the context of clinical trials, and highlight the need for further efforts to make the definition consistent across studies, as thresholds for the exclusion of depression, positive symptoms, and extrapyramidal side effects are not univocally defined and highly heterogeneous across studies [4,14,32,34,38,39,43,44,55]. Furthermore, the minimum prospective persistence required in subjects with a first episode of schizophrenia, in which extensive retrospective data are not available, is still to be defined [14,38].

As to the factor structure of negative symptoms, no recommendation is deemed appropriate by the EPA Guidance Group on Negative Symptoms on the basis of the available evidence. In fact, the two-factor model (with experiential and expressive deficit factors) might be useful to complement total scores in clinical trials, but available confirmatory factor analyses favor a five-factor model [37,71–73]. However, the available evidence relevant to the five-factor model is provided by one group of researchers and needs independent replications before allowing a recommendation.

In the last decades, the assessment of negative symptoms progressed with the development of second-generation clinician-rated scales and self-rated instruments with better assessment of experiential negative symptoms, with respect to first-generation rating scales. However, these latter scales are still largely used in clinical trials. This guidance paper provides evidence-based recommendations for using second-generation scales, such as the BNSS and CAINS; we also provide evidence for complementing the use of first-generation scales with the second-generation ones. The recommendation is of grade B as head-to-head comparisons of first- and second-generation instruments are still limited, and sensitivity to change of second-generation assessment instruments is not fully established (Tables e3 and e5).

Self-assessments of negative symptoms have been recently developed and necessitate further studies, carried out by independent groups. However, they provide complementary information to hetero-assessments and their use as complementary measures to clinician-rated scales might be pursued as a measure of the internal experience of the subjects presenting negative symptoms.

For first-generation rating scales, for example, SANS, PANSS, and, BPRS, this guidance paper provides a summary of evidence (i.e., confirmatory factor analyses and systematic reviews) supporting the exclusion of several items from negative symptom summary scores or subscale scores (Table e2). The comprehensive review of the evidence and the elaboration of a recommendation of grade B might contribute to advance the field, allowing a better assessment of negative symptoms, avoiding overlaps with other psychopathological dimensions, and cognitive impairment.

The guidance provides a systematic review also of the state of the art of assessment in first-episode and CHR subjects, highlighting the need of extending to early psychosis the use of second-generation scales and further development of these instruments in CHR subjects.

Evidence for the differentiation between primary and secondary negative symptoms in routine clinical practice is still limited. The present guidance paper provides several recommendations of grade C and D, which might assist clinicians in the above differentiation and in the identification of treatable causes of secondary negative symptoms (Table e8).

The low grade of these recommendations reflects the limited literature available in spite of the clinical relevance of the identification of secondary negative symptoms to improve the care of people with schizophrenia.

## Conclusions

After more than 15 years from the NIMH consensus initiative on negative symptoms and notwithstanding the development of assessment instruments reflecting the large consensus on the definition of different domains of negative symptoms, the assessment of these symptoms is still to be improved both in research and clinical settings.

This guidance paper is aimed to instigate the adoption of shared assessment protocols both in clinical trials and routine clinical practice, paving the way to further progress in the field of negative symptom recognition.

In clinical trials, the use of first-generation rating scales alone and the inclusion of items that are not part of the negative symptom construct in summary scores of negative symptom should be avoided. The systematic inclusion of second-generation scales is encouraged and might move forward the field of assessment of negative symptoms as these scales provide a better assessment of the experiential domains.

To reinforce the assessments of the latter domains, self-assessments can be associated.

Priority should also be given to the use of second-generation scales in first-episode subjects and further adaptation of these scales to develop negative symptom scales for CHR states, with extended item selection mapping the breadth of negative symptoms in these states. Improved assessment of negative symptoms in CHR might advance the field of early recognition of subjects at risk for schizophrenia and poor outcome as these symptoms often precede the positive ones and predict impaired real-life functioning.

Studies specifically aimed to assess secondary negative symptoms in subjects with schizophrenia at all stages of the disorder should be carried out to optimize the recognition and management of these negative symptoms, which cause significant disability and are often amenable to treatment.

Rigorous longitudinal studies aimed to assess the natural course of negative symptoms are highly needed. They should include clear procedures for the identification of secondary negative symptoms and the reduction of potential underlying sources (extrapyramidal side effects, depression, positive symptoms, isolation, and hypostimulation).

To this aim, training of psychiatrists should focus more on careful and up-to-date assessment of negative symptoms, including the assessment of internal experience and promotion of self-report of negative symptoms.

However, much remains to be done to achieve a standardization of the persistent negative symptom construct, effective strategies for the identification of secondary negative symptoms in routine clinical practice, and to establish the sensitivity to change of second-generation scales.

The dissemination of this guidance paper may promote the development of national guidelines on negative symptom assessment and ultimately improve the care of people with schizophrenia.

## Data Availability

All data supporting the findings of this study are available within the article and its supplementary materials.

## Supplementary material

For supplementary material accompanying this paper visit https://doi.org/10.1192/j.eurpsy.2021.11. click here to view supplementary material
