# Supplementary material for: EPA guidance on assessment of negative symptoms in schizophrenia
Source: Eur Psychiatry. 2021 Feb 8;64(1):e23. doi: 10.1192/j.eurpsy.2021.11 (PMC8080207; doi:10.1192/j.eurpsy.2021.11)
Supplement: Supplementary file 1 [file S0924933821000110sup001.docx]

**Index**

- **eTable 1. Level of evidence and main results of included studies on the classification of negative symptoms**
- **eTable 2. Level of evidence, methods and main results of included studies on the factor structures of general psychopathological rating scales**
- **eTable 3.** **Level of evidence, methods and main results of included studies on the factor structure of negative symptoms**
- **eTable 4. Evaluation of the 5 negative symptom dimensions in 17 scales**
- **eTable 5. List of included reviews, their methods and process of analysis, the main results and comments including evidence ratings on negative symptoms in schizophrenia**
- **eTable 6. List of included reviews, their methods and process of analysis, the main results and comments including evidence ratings on apathy in schizophrenia**
- **eTable 7. Review of negative symptoms assessments in larger-scale intervention studies in the CHR population**
- **eTable 8. Level of evidence, methods and main results of included studies on the differential diagnosis between primary and secondary negative symptoms in the clinical practice**
- **References**

**eTable 1. Level of evidence and main results of included studies on the classification of negative symptoms.**

| **Study** | **Type of study** | **Level of evidence** | **Results** |
| --- | --- | --- | --- |
| **Deficit Schizophrenia (DS)** | | | |
| Kirkpatrick et al., 2001 [1] | Review | III | -Construct validity of DS.  -DS conceptualized as a separate disease with respect to non-deficit schizophrenia (NDS) as it differed from NDS in terms of signs and symptoms, course of illness, risk factors, biological correlates, as well as treatment response. These differences were not confounded by demographic features, antipsychotic treatment, severity of psychotic symptoms or drug abuse and were not more of the same observed in NDS, as it would be expected if DS was a more severe form of the disease.  -Although the diagnosis of deficit schizophrenia is reliable with the use of SDS, and the inclusion of DS subjects in clinical trials has the advantage to overcome problems that might arise in the interpretation of treatment response, excluding possible sources of secondary negative symptoms, this conceptualization has important limits: it requires the use of the Schedule for the Deficit Syndrome (SDS), examiners trained in the use of this instrument. |
| Buchanan, 2007 [2] | Review | III | -Construct validity of DS.  -DS conceptualized as a separate disease with respect to non-deficit schizophrenia (NDS) as it differed from NDS in terms of signs and symptoms, course of illness, risk factors, biological correlates, as well as treatment response.  -Although the diagnosis of deficit schizophrenia is reliable with the use of SDS, and the inclusion of DS subjects in clinical trials has the advantage to overcome problems that might arise in the interpretation of treatment response, excluding possible sources of secondary negative symptoms, this conceptualization has important limits: it requires the use of the Schedule for the Deficit Syndrome (SDS), examiners trained in the use of this instrument and the longitudinal observation that is not always available, especially in first episode patients. |
| Kirkpatrick and Galderisi, 2008 [3] | Review | III | -Construct validity of DS.  -DS was conceptualized as a separate disease with respect to non-deficit schizophrenia (NDS) as it differed from NDS in terms of signs and symptoms, course of illness, risk factors, biological correlates, as well as treatment response.  -Some studies reported discrepant findings regarding differences between DS and NDS in terms of clinical and neurobiological features. This could be due to different factors, such as the clinical characterization of the patients. |
| Galderisi and Maj, 2009 [4] | Review | III | -Construct validity of DS.  -DS was conceptualized as a separate disease with respect to non-deficit schizophrenia (NDS) as it differed from NDS in terms of signs and symptoms, course of illness, risk factors, biological correlates, as well as treatment response.  -Some studies reported discrepant findings regarding differences between DS and NDS in terms of clinical and neurobiological features. This could be due to different factors, such as the assessment instruments used (DS or proxy).  -Although the diagnosis of deficit schizophrenia is reliable with the use of SDS, and that the inclusion of DS subjects in clinical trials has the advantage to overcome problems that might arise in the interpretation of treatment response, excluding possible sources of secondary negative symptoms, this conceptualization has important limits: it requires the use of the Schedule for the Deficit Syndrome (SDS), examiners trained in the use of this instrument and the longitudinal observation that is not always available, especially in first episode patients. |
| Kirkpatrick, 2014 [5] | Review | III | -Construct validity of DS.  -DS was conceptualized as a separate disease with respect to non-deficit schizophrenia (NDS) as it differed from NDS in terms of signs and symptoms, course of illness, risk factors, biological correlates, as well as treatment response. These differences were not confounded by demographic features, antipsychotic treatment, severity of psychotic symptoms or drug abuse and were not more of the same observed in NDS, as it would be expected if DS was a more severe form of the disease. |
| Kirkpatrick et al., 2017 [6] | Review | III | -Construct validity of DS.  -DS was conceptualized as a separate disease with respect to non-deficit schizophrenia (NDS) as it differed from NDS in terms of signs and symptoms, course of illness, risk factors, biological correlates, as well as treatment response.  -Some studies reported discrepant findings regarding differences between DS and NDS in terms of clinical and neurobiological features. This could be due to different factors, such as the assessment instruments used (DS or proxy) or the clinical characterization of the patients. Although the diagnosis of deficit schizophrenia is reliable with the use of SDS, and the inclusion of DS subjects in clinical trials has the advantage to overcome problems that might arise in the interpretation of treatment response, excluding possible sources of secondary negative symptoms, this conceptualization has important limits: it requires the use of the Schedule for the Deficit Syndrome (SDS) and examiners trained in the use of this instrument. |
| Bucci et al., 2017 [7] | Review | II | -Construct validity of DS.  -DS was conceptualized as a separate disease with respect to non-deficit schizophrenia (NDS) as it differed from NDS in terms of signs and symptoms, course of illness, risk factors, biological correlates, as well as treatment response.  -Some studies reported discrepant findings regarding differences between DS and NDS in terms of clinical and neurobiological features. This could be due to different factors, such as the assessment instruments used (DS or proxy).  -Although the diagnosis of deficit schizophrenia is reliable with the use of SDS, and the inclusion of DS subjects in clinical trials has the advantage to overcome problems that might arise in the interpretation of treatment response, excluding possible sources of secondary negative symptoms, this conceptualization has important limits: it requires the use of the Schedule for the Deficit Syndrome (SDS), examiners trained in the use of this instrument and the longitudinal observation that is not always available, especially in first episode patients. |
| Mucci et al., 2017 [8] | Review | III | -Construct validity of DS.  -DS was conceptualized as a separate disease with respect to non-deficit schizophrenia (NDS) as it differed from NDS in terms of signs and symptoms, course of illness, risk factors, biological correlates, as well as treatment response.  -Some studies reported discrepant findings regarding differences between DS and NDS in terms of clinical and neurobiological features. This could be due to different factors, such as the assessment instruments used (DS or proxy) or the clinical characterization of the patients. Although the diagnosis of deficit schizophrenia is reliable with the use of SDS, and the inclusion of DS subjects in clinical trials has the advantage to overcome problems that might arise in the interpretation of treatment response, excluding possible sources of secondary negative symptoms, this conceptualization has important limits: it requires the use of the Schedule for the Deficit Syndrome (SDS), examiners trained in the use of this instrument and the longitudinal observation that is not always available, especially in first episode patients. |
| Galderisi et al., 2018 [9] | Review | II | -Construct validity of DS.  -DS was conceptualized as a separate disease with respect to non-deficit schizophrenia (NDS) as it differed from NDS in terms of signs and symptoms, course of illness, risk factors, biological correlates, as well as treatment response. |
| **Persistent Negative Symptoms (PNS)** | | | |
| Buchanan, 2007 [2] | Review | III | -Construct validity of PNS.  -PNS identifies a patient population larger than the one with DS; it requires less longitudinal observation than the DS; it allows the control of potential sources of indirect changes of negative symptoms during the course of clinical trials.  -The definition of PNS might facilitate the selection of subjects for inclusion in clinical trials.  -However, PNS construct allows the use of any validated psychopathological rating scale, including also those scales, such as SANS and PANSS, that include items which do not assess negative symptoms; threshold of confounding factors (positive, depressive, extrapyramidal symptoms) are not uniquely defined across the studies. |
| Bucci et al., 2017 [7] | Review | III | -Construct validity of PNS.  -PNS identifies a patient population larger than the one with DS; it requires less longitudinal observation than the DS; it allows the control of potential sources of indirect changes of negative symptoms during the course of clinical trials.  -However, PNS construct allows the use of any validated psychopathological rating scale, including also those scales, such as SANS and PANSS, that include items which do not assess negative symptoms; threshold of confounding factors (positive, depressive, extrapyramidal symptoms) are not uniquely defined across the studies. |
| Mucci et al., 2017 [8] | Review | III | -Construct validity of PNS.  -PNS identifies a patient population larger than the one with DS; it allows the control of potential sources of indirect changes of negative symptoms during the course of clinical trials.  -However, PNS construct allows the use of any validated psychopathological rating scale, including also those scales, such as SANS and PANSS, that include items which do not assess negative symptoms; threshold of confounding factors (positive, depressive, extrapyramidal symptoms) are not uniquely defined across the studies. |
| Galderisi et al., 2018 [9] | Review | III | -Construct validity of PNS.  -PNS identifies a patient population larger than the one with DS; it allows the control of potential sources of indirect changes of negative symptoms during the course of clinical trials.  -However, PNS construct allows the use of any validated psychopathological rating scale, including also those scales, such as SANS and PANSS, that include items which do not assess negative symptoms; threshold of confounding factors (positive, depressive, extrapyramidal symptoms) are not uniquely defined across the studies. |
| **Predominant Negative Symptoms** | | | |
| Galderisi et al., 2018 [9] | Review | III | -No construct validity.  -Large heterogeneity in its definition.  -A threshold for secondary negative symptoms is required but not defined.  -It identifies a patient population larger than the one with DS.  - It does not require a longitudinal observation. |
| Bucci et al., 2017 [7] | Review | III | -No construct validity.  -Large heterogeneity in its definition.  -A threshold for secondary negative symptoms is required but not defined.  -It does not require a longitudinal observation. |
| Mucci et al., 2017 [8] | Review | III | -No construct validity.  -Large heterogeneity in its definition.  -It includes a mixture of primary and secondary negative symptoms with a possible large fluctuation over time, thus possibly confounding the results of clinical trials. |
| Marder et al., 2013 [10] | Expert International Conference | III | -No agreement on whether predominant or prominent negative symptoms should be required in designing clinical trials. |
| **Prominent Negative Symptoms** | | | |
| Mucci et al., 2017 [8] | Review | III | -No construct validity.  -It includes a mixture of primary and secondary negative symptoms with a possible large fluctuation over time, thus possibly confounding the results of clinical trials. |
| Marder et al., 2013 [10] | Expert International Conference | III | -No agreement on whether predominant or prominent negative symptoms should be required in designing clinical trials. |

**eTable 2. Level of evidence, methods and main results of included studies on the factor structures of general psychopathological rating scales.**

| **Study** | **Type of study** | **Level of evidence** | **Statistics** | **Assessment Instruments** | **Items** | **Sample size** | **Results** |
| --- | --- | --- | --- | --- | --- | --- | --- |
| Kay and Sevy, 1990 [11] | EFA | II | PCA | PANSS | 30 items | 240 SCZ | Seven factor model: 1. Negative (excluding Difficulty in abstract thinking 🡪 Cognitive and Stereotyped thinking 🡪 Stereotyped thinking) -Emotional withdrawal -Passive/apathetic social withdrawal -Lack of spontaneity & flow of conversation -Blunted affect, -Poor rapport, -Poor attention, -Active social avoidance, -Motor retardation, -Disturbance of volition, -Mannerisms & posturing;  2. Positive; 3. Excited; 4. Depressive; 5. Cognitive; 6. Suspicious/persecutory; 7. Stereotyped thinking. |
| Bell et al., 1993 [12] | EFA;  CFA | II | PCA; covariance matrices | PANSS | 30 items | 146 SCZ (divided into two samples) | EFA Five factor model (first sample): 1. Negative (exc Difficulty in abstract thinking🡪Cognitive; Stereotyped thinking 🡪 Positive): -Blunted Affect, -Emotional withdrawal, -Poor rapport, -Passive/apathetic social withdrawal,  -Lack of spontaneity and flow of conversation, -Motor retardation, -Active social avoidance ;  2. Positive; 3. Cognitive discomfort; 4. Hostility.  EFA Five factor model (second sample): 1. Negative (exc Difficulty in abstract thinking and Stereotyped thinking 🡪 Cognitive discomfort): -Blunted Affect, -Emotional withdrawal, -Poor rapport, -Passive/apathetic social withdrawal, -Lack of spontaneity and flow of conversation, -Motor retardation,  -Disturbance of volition, -Preoccupation;  2. Positive; 3. Cognitive discomfort; 4. Hostility  CFA: poor fit of this model |
| Lindnstrom and Knorring, 1993 [13] | EFA | II | PCA | Swedish version of PANSS | 30 items | 120 SCZ | Five-factor model:  1. Negative (exc. difficulty in abstract thinking 🡪Cognitive; stereotyped thinking): -Blunted affect, -Emotional withdrawal, -Poor rapport, -Passive/apathetic social withdrawal, -Lack of spontaneity and flow of conversation, -Motor retardation, -Active social avoidance,  2. Excitement; 3. Anxious/Depression; 4. Positive; 5.Cognitive. |
| Kawasaki et al., 1994 [14] | EFA | III | PCA | PANSS | 30 items | 70 SCZ | Five-Factor model: 1. Negative (exc Difficulty in abstract thinking 🡪 Thought  disordered): -Blunted affect; -Emotional withdrawal; -Poor rapport, -Passive social withdrawal, -Lack of spontaneity, -Stereotyped thinking, -Mannerism, -Motor retardation, -Disturbance of volition; 2. Hostile excited; 3. Thought Disordered; 4. Delusions and Hallucinations; 5. Depressive. |
| Lindenmayer et al., 1994 [15] | EFA | II | PCA | PANSS | 30 items | 240 SCZ | Five-factor model: 1. Negative (exc Difficulty in abstract thinking 🡪 Cognitive and Stereotyped thinking): -Blunted affect, -Emotional withdrawal, -Poor rapport, -Passive/apathetic social withdrawal, -Lack of spontaneity, -Active social avoidance;  2. Excited;  3. Cognitive; 4. Positive; 5. Depressive. |
| Lindenmayer et al., 1994 [16] | EFA | II | PCA | PANSS | 30 items | 240 SCZ | Five-factor model: 1. Negative (exc Difficulty in abstract thinking 🡪 Cognitive and Stereotyped thinking): -Blunted affect, -Emotional withdrawal, -Poor rapport, -Passive/apathetic social withdrawal, -Lack of spontaneity, -Active social avoidance;  2. Excited;  3. Cognitive; 4. Positive; 5. Depressive. |
| White et al., 1997 [17] | CFA | II | Satorra-Bentler X2; CFI | PANSS | 25 items  (exc. P2, P6, G10, G12, G16) | 1233 SCZ or schizoaffective | Five factor model  1. Negative (exc. difficulty in abstract thinking and stereotyped thinking🡪Disorganization): -Blunted affect, -Emotional withdrawal, -Poor rapport, -Passive/apathetic social withdrawal, -Lack of spontaneity and flow of conversation (uncooperativeness and impulsivity as secondary loading), - Mannerism and posturing, - Motor retardation;  2. Positive; 3. Activation; 4. Dysphoric mood; 5. Autistic preoccupation. |
| Dollfus and Everitt, 1998 [18] | CFA | II | Chi square | SAPS; SANS; **PANSS** | PANSS 25 items (exc N7: Stereotyped thinking, G7, G8, G12 and G13) | 135 SCZ | Three-factor model 1. Negative (exc Difficulty in abstract thinking 🡪Disorganization): -Blunted Affect; -Emotional withdrawal; -Poor rapport -Passive/apathetic social withdrawal; -Lack of spontaneity and flow of conversation;  2. Positive;  3. Disorganization. |
| Lancon et al., 1998 [19] | CFA | II | PCA | PANSS | 24 items (exc N7 Stereotyped thinking, G5, G11, G12, G13, G15) | 205 SCZ | Five factor model: 1. Negative (exc Difficulty in abstract thinking 🡪 Cognitive): -Blunted affect, -Emotional withdrawal, -Poor rapport, -Passive/apathetic social withdrawal, -Lack of spontaneity, -Motor retardation , -Active social avoidance;  2. Positive; 3. Activation; 4. Depressive; 5. Cognitive. |
| Nakaya et al., 1999 [20] | CFA | II | GFI; AGFI; NFI; NNFI | PANSS | 28 items (exc. G15 and G16) | 100 SCZ or schizophreniform disorder patients | Five-factor model:  1. Negative (exc. emotional withdrawal and passive/apathetic social withdrawal🡪 Relational; Difficulty in abstract thinking and Stereotyped thinking): -Blunted affect,  -Poor rapport, -Lack of spontaneity and flow of conversation;  2. Positive; 3. Disorganization; 4. Relational; 5. Excitement.  In the chronic stable phase, only the ﬁve-dimensional model adequately ﬁts the data, while during the acute phase a three, four and five models ﬁt the data relatively well. |
| Lykouras et al., 2000 [21] | EFA;  CFA | II | PCA | PANSS | 30 items | 258 SCZ | Five-factor model:  1. Negative (exc. difficulty in abstract thinking and stereotyped thinking🡪Cognitive): -Blunted affect, -Emotional withdrawal, -Poor rapport, -Passive/apathetic social withdrawal, -Lack of spontaneity and flow of conversation, -Motor retardation,  2. Excitement; 3. Depression; 4. Positive; 5.Cognitive.  CFA: poor fit of this model |
| Mass et al., 2000 [22] | EFA | II | PCA;  CFI | PANSS | 30 items | 253 SCZ | Five-factor model: 1. Negative (exc. Difficulty in abstract thinking🡪Cognitive): -Blunted affect, -Emotional withdrawal, -Poor rapport, -Passive/apathetic social withdrawal, -Lack of spontaneity and flow of conversation, -Active social avoidance;  2. Positive; 3. Depression; 4. Hostile excitement; 5. Cognitive |
| Lancon et al., 2000 [23] | EFA | II | PCA | PANSS | 24 items (exc N7: Stereotyped thinking, G1, G5, G8, G13, G15) | 118 SCZ in acute phase and 224 SCZ in chronic phase | Five factor model: 1. Negative (exc Difficulty in abstract thinking 🡪 Cognitive): -Blunted affect, -Emotional withdrawal, -Poor rapport, -Passive/apathetic social withdrawal, -Lack of spontaneity, -Motor retardation,  -Active social avoidance,  2. Positive; 3. Excitation; 4. Depression; 5. Cognitive. |
| Wolthaus et al., 2000 [24] | Multicenter study | II | PCA | PANSS | 30 items | 138 recent-onset schizophrenia patients | Five factor model:  1. Negative (exc. difficulty in abstract thinking and stereotyped thinking🡪Disorganization): -Blunted affect, -Emotional withdrawal, -Poor rapport, -Passive/apathetic social withdrawal, -Lack of spontaneity and flow of conversation, -Motor retardation, -Active social avoidance;  2. Positive; 3. Depression; 4. Agitation-excitement; 5. Disorganization. |
| Emsley et al., 2003 [25] | EFA | II | PCA | PANSS | 30 items | 535 SCZ | Five-factor model:  1. Negative (exc Difficulty in abstract thinking and Stereotyped thinking 🡪 Disorganization): -Blunted affect, -Emotional withdrawal, -Poor rapport, -Passive social withdrawal, -Lack of spontaneity, -Motor retardation, -Disturbance of volition, -Active social avoidance;  2. Disorganization; 3. Positive; 4. Excited factor; 5. Depressive and Anxiety. |
| Fitzgerald et al., 2003 [26] | CFA | II | Chi square; CFI | PANSS | 25 items (exc P2, P6, G10, G12 and G16) | 347 SCZ | Five-factor model had inadequate goodness of fit.  Five factor model: 1. Negative (exc Difficulty in abstract thinking and Stereotyped thinking 🡪 Autistic preoccupation): -Blunted affect, -Emotional withdrawal, -Poor rapport, -Passive social withdrawal, -Lack of spontaneity, -Mannerism and posturing, -Motor retardation,  2. Positive;  3. Activation;  4. Dysphoric; 5. Autistic preoccupation. |
| Lee et al., 2003 [27] | EFA | II | PCA | PANSS | 30 items | 105 SCZ | Five factor model: 1. Negative (exc Difficulty in abstract thinking and Stereotyped thinking 🡪Disorganisation): -Blunted affect, -Emotional withdrawal, -Poor rapport, -Passive/apathetic social withdrawal. -Lack of spontaneity, -Motor retardation, -Active social avoidance;  2. Disorganisation; 3. Reality distortion; 4. Excitement; 5. Depression. |
| Fresan et al., 2005 [28] | EFA | II | PCA | PANSS | 28 items (exc P5 and G10) | 150 SCZ | Five-factor model: 1. Negative (exc Difficulty in abstract thinking, Lack of spontaneity and flow of conversation and Stereotyped thinking 🡪 Cognitive): -Emotional withdrawal, -Apathetic social withdrawal;  -Blunted affect, -Poor rapport, -Disturbance of volition, -Poor attention;  2. Excitement; 3. Positive; 4. Anxiety/Depression; 5. Cognitive. |
| van der Gaag et al., 2006 [29] | CFA  10-fold cross-validation | II | CFI; RMSEA | PANSS | 30 items | 5769 | Five factor model:  1. Negative (exc. difficulty in abstract thinking and stereotyped thinking🡪Disorganization):  -Blunted affect, -Emotional withdrawal, -Poor rapport, -Passive/apathetic social withdrawal, -Lack of spontaneity and flow of conversation, -Motor retardation, -Uncooperativeness, -Active social avoidance (multiple factor loadings on POS, EXC and EMO);  Secondary factor loadings (P2 and G13)  2. Positive; 3. Emotional Distress; 4. Excitement; 5. Disorganization |
| Wallwork et al., 2012 [30] | CFA | II | Polychoric correlation matrix; CFI; NNFI; RMSEA | PANSS | 20 items (exc. P6, N7-Stereotyped Thinking, G1, G4, G5, G10, G12, G13, G15, G16) | 458 SCZ | Five factor model:  1. Negative (exc. difficulty in abstract thinking 🡪Disorganization): -Blunted affect, -Emotional withdrawal, -Poor rapport, -Passive/apathetic social withdrawal, -Lack of spontaneity and flow of conversation, -Motor retardation;  2. Positive; 3. Depression; 4. Excited; 5. Disorganization |
| Fong et al., 2015 [31] | CFA | II | X2; CFI; RMSEA; TLI | PANSS | 20 items (exc N7: Stereotyped thinking; G1, G4, G5, G10, G12, G13, G15, G16 and P6) | 146 chinese chronic SCZ | Five-factor model: 1. Negative (exc Difficulty in abstract thinking 🡪 Cognitive): -Blunted affect, -Emotional withdrawal, -Poor rapport, -Passive social withdrawal;  2. Positive; 3. Excited; 4. Depressive; 5. Cognitive. |
| Munoz-Negro et al., 2015 [32] | EFA; CFA | II | PCA; Oblimin rotation | PANSS | 30 items | 550 subjects (373 SCZ; 137 delusional disorder; 40 Schizoaffective disorder) | Five factor model:  1. Negative (exc. stereotyped thinking🡪Cognitive): -Blunted affect, -Emotional withdrawal, -Poor rapport, -Passive/apathetic social withdrawal, -Difficulty in abstract thinking, -Lack of spontaneity and flow of conversation, -Motor retardation, -Disturbance of volition, -Active social avoidance;  2. Positive; 3. Depression; 4. Manic; 5. Cognitive |
| Grover et al., 2018 [33] | EFA | II | Factor analysis; Varimax rotation | PANSS | 30 items | 181 SCZ | Three (PANSS) Four- (CDSS +PANSS), Five- (also YMRS), Seven- (also YBOCS) factor model: 1. Negative (exc Difficulty in abstract thinking 🡪 Anxiety): -Blunted affect, -Emotional withdrawal, -Poor rapport, -Passive social withdrawal, -Lack of spontaneity, -Stereotyped thinking, -Motor retardation,  -Active social avoidance;  2. Depressive; 3. Obsessive compulsive; 4. Positive; 5. Manic; 6. Anxiety; 7. Obsessive compulsive-2. |
| Shafer and Dazzi, 2019 [34] | Meta-analysis | II | PCA; Varimax solution | PANSS | 45 factor analyses |  | Five-factor model:  1. Negative symptoms  - Emotional  withdrawal,  - Blunted affect,  - Passive apathetic social withdrawal,  - Lack of spontaneity,  - Poor rapport,  - Motor retardation,  - Active social avoidance;  2. Positive symptoms;  3. Disorganization;  4. Depression-Anxiety;  5. Resistance or Excitement/Activity. |
| Gur at al., 1991 [35] | EFA | III | PCA; Varimax solution | SANS; SAPS | 5 Global Rating of SANS; 4 Global Rating from SAPS | 47 SCZ | Three-factor model: 1. Negative symptoms (exc Attention🡪Bizzarre behavior and thought disorder): -Affective flattening, -Alogia, -Avolition-Apathy, -Anhedonia-asociality;  2. Bizzarre behaviour and Thought disorder; 3. Positive symptoms. |
| Arndt et al., 1991 [36] | EFA | II | PCA | SANS;  SAPS | Five global ratings of SANS and four global ratings of SAPS | 207 SCZ | Three-factor model: 1. Negative: -Avolition, -Anhedonia, -Affective flattening, -Alogia, -Attentional deficit;  2. Positive formal thought disorder and bizarre behaviour; 3. Delusions and hallucinations. |
| Goldman et al., 1991 [37] | EFA | III | PCA | SANS; BPRS | Five global ratings of SANS and 6 BPRS items for positive symptoms | 40 SCZ | Three-factor model (both at baseline and post-treatment): 1. Negative: - Affective flattening, - Alogia, - Avolition/apathy, - Anhedonia-asociality, - Attention;  2. Positive; 3. Behavioural agitation. |
| Peralta et al., 1992 [38] | EFA | II | PCA;  Varimax solution | SANS; SAPS | 5 global ratings from SANS plus Inappropriate affect and Poverty of content of speech; 4 global rating from SAPS | 115 SCZ | Four-factor model: 1. Negative factor; (exc Attention, Inappropriate affect🡪 Disorganization) -Affective flattening, -Alogia, -Avolition, -Anhedonia; - Poverty of Content of speech;  2. Disorganisation factor; 3. Delusions and Hallucination factor; 4. Bizzarre behaviour factor. |
| Brown and White, 1992 [39] | EFA | II | PCA;  Varimax solution | SANS;  Hamilton Rating Scale for Depression positive symptom subscale of the Manchester Scale | 11 items; 8 from SANS (Facial movements, Spontaneous movements, Expressive gestures, Affective responsiveness, Poverty of speech, Poverty of content of speech, Inattentiveness, Inappropriate affect) | 139 SCZ | Three-factor model: 1. Negative symptoms (exc. Inattentiveness and inappropriate affect🡪Disorganization): -Facial movements, -Spontaneous movements, -Expressive gestures, -Affective responsiveness, -Poverty of speech;  2. Psychotic symptoms; 3. Disorganization. |
| Minas et al., 1992 [40] | EFA | II | Correlation matrix with a non-metric multidimensional scaling | SANS; SAPS | Full 20 items from SANS except Inattentiveness because 26 subjects had missing data on this item; full 30 items from SAPS | 114 patients with psychotic disorder | Three-factor model:  1. Negative Symptoms (exc Poverty of content of speech🡪 Thought Disorder; Inappropriate affect that did not fit uniquely with a single symptom dimension; blocking, poor grooming and hygiene🡪low communalities): -Unchanging facial expression, -Decreases spontaneous movements, -Paucity of expressive gestures, -Poor eye contact, -Affective nonresponsivity, -Lack of vocal inflection, -Poverty of speech, -Increased response latency, -Impersistence at work or school, -Physical anergia, -Recreational interests and activities, -Sexual interest and activity, -Ability to feel intimacy and closeness, -Relationships with firends and peers, -Social Inattentiveness;  2. Thought Disorder; 3. Hallucinations/Delusions |
| Malla et al., 1993 [41] | EFA | II | PAF | SAPS;  SANS | -First analysis derived from Liddle: 10 items SANS (derived from affective flattening and alogia subscales exc “blocking”) and 14 items from SAPS  - Second analysis: full 20 items SANS exc “blocking” and 14 items from SAPS | 155 SCZ | First analysis:  Three-factor model: 1. Psychomotor poverty (exc. Inappropriate affect and poverty of content of speech🡪Disorganization):  -Unchanging facial expression, -Decreased spontaneous movements, -Paucity of expressive gestures, -Poor eye contact, -Affective non-responsivity, -Lack of vocal inflections, -Poverty of speech, -Increased latency of response;  2. Disorganization; 3. Reality distortion.  Second analysis:  Three-factor model  1. Psychomotor poverty (exc. Inappropriate affect and poverty of content of speech🡪Disorganization; Grooming and Hygiene; Inattentiveness During mental status testing):  -Unchanging facial expression, -Decreased spontaneous movements, -Paucity of expressive gestures, -Poor eye contact, -Affective non-responsivity, -Lack of vocal inflections, -Poverty of speech, -Increased latency of response, -Impersistence at work and school, -Physical Anergia, -Recreational interests and activities, - Sexual activity, - Ability to feel intimacy and closeness, -Relationships with friends and peers, -Social inattentiveness;  2. Disorganization; 3. Reality distortion. |
| Palacios-Araus et al., 1995 [42] | EFA | III | PCA | SANS; SAPS | Five global ratings of SANS; Four global ratings of SAPS | 67 chronic SCZ | Three-factor model 1. Negative symptoms (exc Attentional impairment, not associated with any specific factor, since it loaded on first and third factors with similar strength): - Affective flattening, - Alogia, - Avolition/apathy, - Anhedonia-asociality;  2. Formal thought disorder/bizarre behavior; 3. Hallucinations /Delusion. |
| McAdams et al., 1997 [43] | EFA | II | PCA; Factor extension analysis | SANS; SAPS; BPRS; | **Five global ratings of SANS,  Four global ratings of SAPS** BPRS | 109 SCZ | First and Second analysis: Three-factor model: 1. Negative symptoms (exc Avolition/apathy 🡪 Depressive): - Affective flattening, - Alogia, - Anhedonia-asociality, - Attention;  2. Positive; 3. Depressive. |
| Grube et al., 1998 [44] | Meta-analysis | II | PCA | SANS;  SAPS or SADS | Five global ratings of SANS; hallucinations, delusions, positive formal  thought disorder and bizarre behavior | 10 studies | Three-factor model: 1. Negative symptoms: - Affective flattening, - Alogia, - Avolition/apathy, - Anhedonia-asociality, - Attention;  2. Positive; 3. Conceptual disorganization. |
| Basso et al., 1998 [45] | EFA | III | PCA; Varimax solution | SAPS;  SANS | 5 items from SANS (Global rating) | 62 SCZ | Three-factor model: 1. Negative symptoms: (exc Inattention) -Flattened affect, -Anhedonia, -Avolition/Apathy, -Alogia;  2. Bizzarre behaviour, Inattention and Thought disorder; 3. Positive symptoms. |
| Dollfus and Everitt, 1998 [18] | CFA | II | Chi square | **SAPS; SANS;** PANSS | 4 global ratings of SANS (no global rating of attention) plus inappropriate affect, ability to feel intimacy and closeness, relationship with friends and peers and 4 global ratings of SAPS | 135 SCZ | Good fit of the three factor-model and four-factor model  Three-factor model:  1. Negative (exc. Alogia, ability to feel intimacy and closeness, relationship with friends; inappropriate affect🡪 Disorganization)  -Affective flattening, -Anhedonia, -Avolition;  Four-factor model 1. Negative (exc. Anhedonia; inappropriate affect🡪 Disorganization; ability to feel intimacy and closeness, relationship with friends🡪 Relational): -Affective flattening, -Alogia, -Avolition;  2. Positive;  3. Disorganization; 4. Relational. |
| Peralta and Cuesta, 1999 [46] | EFA | II | -First-order PCA  -Second order PCA | SANS;  SAPS | full 20 items SANS; full 30 items SAPS | 660 psychotic inpatients (352 SCZ) | - 11 first-order factors (64% variance)  - 4 second-order factors (54% variance):  1. Negative factor (exc Inappropriate affect and Grooming and Hygiene 🡪 Disorganisation; Blocking, Latency of Response, Social Inattentiveness, Inattentiveness During Mental Status Testing);  -Poverty of affect and speech (1^st^ order) (Facial expression, Spontaneus movements, Espressive gestures,  Eye contact,  Affective Nonresposivity, Vocal inflections,  Poverty of speech,  Poverty of contents); -Social dysfunction (1^st^ order)  (Impersistence at work,  Physical Anergia,  Recreational interests,  Sexual interest,  Intimacy and closeness, Relationship with friends);  2. Disorganization; 3. Psychosis; 4. Other Delusion. |
| Cardno, 1999 [47] | CFA | II | Varimax solution; Regression anal | SANS; SAPS; | Global rating of Affective Flattening and Alogia from SANS plus inappropriate affect and poverty of content of speech; 4 Global rating from SAPS | 191 SCZ, grouped as 109 sibling pairs (82 pairs; 9 trios, each counting as 3 pairs) | Three-factor model: 1. Negative (exc inappropriate affect and poverty of content of speech🡪Disorganization): -Affective Flattening, -Alogia;  2. Positive; 3. Disorganisation; |
| Emsley et al., 2001 [48] | EFA | II | PCA | SANS; SAPS | -First and second analyses:  5 Global Ratings (SANS) and 4 Global Rating (SAPS); -Third analysis: 18 items from SANS (exc Sexual Activities and Inattentivenes During Mental Status Testing); Full 30 item SAPS | 422 SCZ | First Analysis: Two-factor model (Global Ratings): 1. Negative symptoms: (exc Attention) -Flattened affect, -Anhedonia, -Avolition/Apathy, -Alogia;  2. Positive symptoms;  Second Analysis: Five-factor model (Global Ratings): 1. Negative symptoms: (exc Alogia 🡪Alogia and Attention 🡪Attention) -Flattened affect, -Anhedonia, -Avolition/Apathy;  2. Positive symptoms; 3. Attention; 4. Alogia; 5. Disorganisation.  Third Analysis: Five-factor model (Items): 1. Psychotic;  2. Diminished Expression (exc. Inappropriate affect and Blocking🡪Thought disorder; Poverty of content of speech, Recreational interests, Intimacy and closeness, Relationships 🡪Disordered related; Grooming and hygiene🡪 Bizzare Behaviour): -Facial movements; -Spontaneous movements; -Expressive gestures; -Poor eye contact; -Affective responsiveness; -Lack of vocal inflections; -Poverty of speech; -Increased latency of response; -Impersistence; -Physical Anergia; -Social Inattentiveness;  3. Thought disorder; 4. Disordered relating; 5. Bizzarre Behavior. |
| Peralta and Cuesta, 2001 [49] | review | II | N/A | N/A | N/A | 26 studies | Three-factor model:  1. Negative: Poverty of affect and speech, avolition, social inappropriateness, social isolation;  2. Catatonia; 3. Psychosis: Hallucinations and delusions. |
| Kulhara and Avasthi, 1993 [50] | EFA | II | PCA | SAPS; SANS | Five global ratings of SANS and four global ratings of SAPS | 151 SCZ | Three- factor model: 1. Negative: - Affective flattening, - Alogia, - Avolition/apathy, - Anhedonia-asociality, - Attention;  2. Reality-distortion;  3. Disorganization |
| Andreasen et al., 1995 [51] | review | II | N/A | N/A | N/A | 5 studies | Three-factor model: 1. Negative;  2. Positive; 3. Disorganization. |
| Niehaus et al., 2005 [52] | EFA | II | PCA | SANS;  SAPS | Three global ratings of SANS (no alogia and attention global rating) and 4 global ratings of SAPS | 208 sib pair SCZ | Five-factor model: 1. Negative symptoms (exc. Affective flattening🡪 Affective changes)  -Avolition, -Anhedonia;  2. Positive symptoms; 3. Thought disorder; 4. Bizzarre behaviour; 5. Affective changes. |
| Mueser et al., 1997 [53] | EFA; CFA | II | Chi-square;  TLI; CFI; RMSEA | BPRS | 18 items (exc suicidality, elevated mood, bizarre behaviour, self-neglect. Distraibility, motor hyperactivity.) | 474 SCZ | First analysis: Four-factor model:  1. Negative:  -Blunted affect -Emotional withdrawal -Motor retardation -Uncoperativeness;  2. Positive symptoms; 3. Affect (depression and anxiety); 4. Disorganization.  Second analysis: Four-factor model:  1. Negative:  -Blunted affect -Emotional withdrawal -Motor retardation;  2. Positive symptoms; 3. Affect (depression and anxiety); 4. Disorganization.  Authors concluded that much greater confidence can be placed in the validity of the four-factor structure identified in the EFA presented earlier. |
| McAdams et al., 1997 [43] | EFA | II | PCA | SANS; SAPS; **BPRS;** | BPRS subscales: -Negative symptom subscale (emotional withdrawal, motor retardation, and blunted affect.) -Positive symptom subscale;  -Depressive symptom subscale. | 109 SCZ | First and Second Analysis: Three-factor model: 1. Negative: -Emotional withdrawal,  -Motor retardation, -Blunted affect;  2. Positive; 3. Depressive. |
| Long et al., 1999 [54] | CFA | II | Chi-square; GFI, CFI, NNFI | BPRS | 18 items as in Mueser et al., 1997[53] to test the four factor model;  16 items (18 items exc excitement and disorientation) | 193 SCZ or schizoaffective subjects | Replication of the four factor model of Mueser et al.,1997.  The results show that the model was configurally invariant over time, but it was more invariant for the 16-item version. |
| Shafer, 2005 [55] | Meta-analysis | II | N/A | BPRS | N/A | 12 studies on SCZ | First analysis: Four-factor model: 1. Negative Symptoms: -Blunted affect, -Emotional withdrawal, -Motor retardation, -Disorientation;  . Positive symptoms; 3. Affect; 4. Activation.  Second analysis: Five-factor model: 1. Negative Symptoms: -Blunted affect, -Emotional withdrawal, -Motor retardation, -Disorientation;  2. Positive Symptoms; 3. Affect; 4. Resistance;  5. Activation. |
| Colasanti et al. , 2010 [56] | EFA | II | PCA | BPRS | Twenty-two items (exc guilt and motor hyperactivation) | 183 psychotic patients (106 with schizophrenia, schizoaffective disorder or Schizophreniform disorder) | Four-factor model: 1. Negative symptoms: -Blunted affect, -Conceptual disorganization, -Self-neglect, -Distractibility, -Disorientation, -Motor retardation, -Emotional withdrawal;  2. Positive symptoms (Resistance); 3. Labelled Excitement (Activation); 4. Depression (Anxiety). |
| Picardi et al., 2012 [57] | CFA | II | Factor mixture analysis | BPRS | 24 items | 239 SCZ | Five-factor model: 1. Negative Symptoms: -Blunted affect, -Emotional withdrawal, -Motor retardation, - Self neglect;  2. Positive Symptoms; 3. Disorganization; 4. Depression; 5. Activation. |

EFA=explorative factor analysis; CFA=confirmatory factor analysis; PCA=principal component analysis; SCZ: subjects with schizophrenia; PANSS: Positive and Negative Syndrome Scale; BPRS: Brief Psychiatric Rating Scale; SAPS: Scale for the Assessment of Positive Symptoms; SANS: Scale for the Assessment of Negative Symptoms; PRS: Psychopathology Rating Scale; SADS: Schedule for Affective Disorders and Schizophrenia; CFI=comparative fit index; NFI= normed ﬁt index; NNFI= Non-Normed Fit Index; RMSEA= Root Mean Square Errors of Approximation; GFI= goodness-of-ﬁt-index; AGFI= adjusted for degree of freedom; TLI=Tucker-Lewis index; YMRS= Young mania rating scale; YBOCS=Yale Brown Obsessive Compulsive Checklist; PAF= principal axis factor

**eTable 3.** **Level of evidence, methods and main results of included studies on the factor structure of negative symptoms.**

| **Study** | **Type of study** | **Level of evidence** | **Statistics** | **Assessment Instruments** | **Items** | **Sample size** | **Results** |
| --- | --- | --- | --- | --- | --- | --- | --- |
| Blanchard and Cohen, 2006 [58] | Review | II | N/A | N/A | N/A | N/A | The most replicated model is the two-factor model:  1. Experiential factor; 2. Expressive factor. |
| Marder and Galderisi, 2017 [59] | Review | II | N/A | N/A | N/A | N/A | The most replicated model is the two-factor model:  1. Experiential factor; 2. Expressive factor. |
| Bucci and Galderisi, 2017 [7] | Review | II | N/A | N/A | N/A | N/A | The most replicated model is the two-factor model:  1. Experiential factor; 2. Expressive factor. |
| Galderisi et al., 2018 [9] | Systematic review | II | N/A | N/A | N/A | N/A | The most replicated model is the two-factor model:  1. Experiential factor; 2. Expressive factor. |
| Strauss et al., 2019 [60] | Review | II | N/A | N/A | N/A | N/A | Five-factor model:  1. Avolition; 2. Anhedonia; 3. Asociality; 4. Blunted affect; 5. Alogia. |
| Gibbons et al., 1985 [61] | CFA | II | likelihood ratio x2  statistic;  Models tested:  1-factor model  2-factor model  3-factor model | Inpatient Multidimensional Psychiatric Scale | 78 items | 416 SCZ | Three-factor model for negative symptoms: 1. Apathy: -Apathy toward treatment, -Apathy toward the environment, -Fixed facial expression;  2. Psychomotor retardation: -Slow speech, -Fixed facial expression, -Slow movement, -Thought blocking, -Poverty of speech;  3. Loss of goal: -Incoherent speech, -Irrelevant speech, -Wandering speech, -Inappropriate affect. |
| Axelrod et al., 1994 [62] | CFA | II | Satorra-Bentler x2 test;  Models tested:  6-factor model  5-factor model  1-factor model  Null-model | NSA | Full 26 items NSA | 223 SCZ | Six-factor model:  1. Communication: -Prolonged time to respond; -Restricted speech quantity, -Impoverished speech content, -Failure to answer, -Inarticulate speech;  2. Emotion/affect: -Blank, expressionless face, -Emotion: reduced range, -Affect: reduced modulation, -Affect: reduced display;  3. Social Involvement: -Reduced social drive, -Poor rapport with interviewer, -Avoids looking with interviewer, Reduced sexual interest;  4. Motivation: -Poor grooming and hygiene, -Reduced sense of purpose, -Reduced hobbies and interests, -Reduced daily activity;  5. Gross Cognition: -Poor abstraction; -Poor memory, -Temporal disorientation;  6. Retardation: -Slow speech; -Monotonous speech, -Muted speech, -Reduced expressive gestures. |
| Keefe at al., 1992 [63] | CFA | II | PCA;  Models tested:  1-factor model  2-factor model  3-factor model  4-factor model  Null-model | SANS | 13 of 20 (exc Poor eye contact, Blocking, Increased Latency of Response, Recreational Interests and Activities, Sexual Activity, Social Inattentiveness, Inattentiveness During Mental Status) | 130 males hospitalized SCZ | Three-factor model:  1. Diminished expression: -Paucity of expressive gestures, -Decreased spontaneous movements,  -Unchanging facial expression -Affective nonresponsivity, -Lack of vocal inflections, -Poverty of speech, -Physical anergia;  2. Social Dysfunction: -Ability to feel intimacy/closeness, -Relationships with friends and peers, -Grooming and hygiene, -lmpersistence at work or school;  3. Disorganization: -Inappropriate affect -Poverty of content of speech. |
| Mueser et al., 1994 [64] | EFA | II | PCA | SANS | Full 20 items SANS | 207 SCZ | Three factor model: 1. Affective flattening/blunting subscales; 2. Avolition/apathy and Anhedonia/asociality subscales; 3. Alogia and Inattention subscales. |
| Peralta and Cuesta, 1995 [65] | CFA | II | ICC;  Models tested:  1-factor model  2-factor model (in accordance with Miller et al., 1993)  2-factor model (in accordance with Strauss et al., 1974)  3-factor model  5-factor model  5-factor model  (in accordance with Andreasen et al., 1986)  Null-model | SANS | Full 20 items SANS | 253 SCZ | Five-factor model: 1. Affective flattening: -Unchanging facial expression; -Decreased spontaneous movements, -Paucity of expressive gestures, -Poor eye contact, -Affective nonresponsivity,  -Lack of vocal inflections;  2. Alogia: -Poverty of speech, -Poverty of content of speech, -Blocking, -Increased latency of response;  3. Avolition-apathy: -Grooming and hygiene, -Impersistence at work, -Physical anergia;  4. Anhedonia-asociality: -Recreational interests,  -Sexual interest and activity,  -Ability to feel intimacy, -Relationship with friends;  5. Attention: -Social Inattentiveness, -Inattentiveness during testing.  the model excluding inappropriate affect from the SANS was the best adjusted. |
| Sayers et al., 1996 [66] | EFA; CFA | II | ICC;  Models tested:  1-factor model  3-factor model  5-factor model | SANS | 19 of 20 (exc inappropriate affect) | 457 SCZ | EFA Three-factor model:  1. Diminished Expression: -Unchanging facial expression; -Decreased spontaneous movements, -Paucity of expressive gestures, -Affective nonresponsivity,  -Lack of vocal inflections, -Poverty of speech;  2. Inattention-alogia: -Poverty of content of speech, -Blocking, -Increased latency of response, -Social Inattentiveness, -Inattentiveness during testing;  3. Social Amotivation: -Grooming and hygiene, -Impersistence at work, -Physical anergia, -Recreational interests, -Sexual interest and activity, -Ability to feel intimacy and closeness, -Relationship with friends and peers;  CFA confirmed the EFA results |
| Kelley et al., 1999 [67] | EFA | II | PCA | SANS | full 20 items SANS | 93 SCZ or schizoaffective | Two-factor model:  1. Affective Flattening: -Unchanging facial expression; -Decreased spontaneous movements, -Paucity of expressive gestures, -Affective nonresponsivity,  -Lack of vocal inflections, -Poverty of speech;  2. Diminished Motivation: -Grooming and hygiene, -Impersistence at work, -Physical anergia, -Recreational interests, -Sexual interest and activity, -Ability to feel intimacy and closeness, -Relationship with friends and peers.  The remaining factors included what have been found to be disorganization/positive symptom items, which are considered to be dimensions independent of negative symptoms and thus were not pursued in further analysis (Inappropriate affect, Poor Eye Contact, Poverty of content of speech, Blocking, Increased latency of response, Social Inattentiveness, Inattentiveness during testing). |
| Strauss et al., 2013 [68] | EFA | II | PCA | SANS;  SDS | 4 Global Score (exc Inattention); 6 items. | Study 1: 199 SCZ (SANS)  Study 2: 169 deficit SCZ (SDS) | Two-factor model:  1. Avolition-Apathy  SANS: -Affective flattening, -Alogia, SDS: -Curbed interest, -Diminished sense of purpose, -Diminished social drive;  2. Diminished expression SANS: -Avolition; -Anhedonia-Asociality; SDS: -Restricted affect, -Diminished emotional range, -Poverty of speech. |
| Ergul and Ucok, 2015 [69] | EFA | II | PCA | SANS | 4 Global Score (exc Inattention). | 174 drug-naive FEP | Two-factor model at baseline: 1. Expressive deficit (ED): -Alogia, -Blunted affect; 2. Motivation-Pleasure Deficit (MPD): -Avolition, -Anhedonia.  One factor model after 12 and 24 months follow-up |
| Liemburg et al., 2013 [70] | EFA;  CFA | II | PAF; asymptotic covariance matrix and comparative fit indices;  Models tested:  1-factor model  2-factor model | PANSS | N1 Flat affect, N2 Emotional withdrawal, N3 Poor rapport, N4 Passive/ apathetic social withdrawal, N6 Lack of spontaneity, G5 Mannerisms and posturing, G7 Motor retardation, G13 Avolition, and G16 Active social avoidance | 664 for EFA; 2172 for CFA | Two-factor model:  1. Expressive Deficit:  -Flat affect, -Poor rapport, -Lack of spontaneity,  -Mannerisms and posturing, -Motor retardation,  -Avolition;  2. Social Amotivation:  -Emotional withdrawal, -Passive/apathetic social withdrawal, -Active social avoidance.  The CFA confirmed the EFA |
| Stiekema et al., 2016 [71] | CFA | II | Correlation matrix | PANSS | N1-N4, N6, G5, G7, G13, G16 | 1157 chronic psychotic patients | Two-factor model:  1. Social Amotivation:  -Emotional Withdrawal, -Passive/Apathetic social withdrawal, -Active social avoidance;  2. Expressive Deficits:  -Blunted affect, -Poor rapport, -Lack of spontaneity, -Mannerism and Posturing, -Motor retardation, -Disturbance of volition. |
| Jang et al., 2016 [72] | CFA | II | Correlation analysis;  Models tested:  1-factor model  2-factor model | PANSS | N1, N2, N3, N4, N6, G7, and G16 | 220 SCZ | Two-factor model:  1. Expressive Deficits:  -Blunted affect, -Poor rapport, -Lack of spontaneity, -Motor retardation,  2. Experiential Deficits: -Emotional Withdrawal, -Passive/Apathetic social withdrawal, -Active social avoidance. |
| Kimhy et al., 2006 [73] | EFA | II | PCA | SDS | 6 items | 52 DS | Two factor model:  1. Avolition:  -Curbing of interest, -Diminished social drive, -Diminished sense of purpose;  2. Emotional Expression: -Restricted affect, -Diminished emotional range, -Poverty of speech. |
| Nakaya and Ohmori, 2008 [74] | EFA | II | PCA | SDS | 6 items | 70 DS | Two factor model:  1. Avolition:  -Curbing of interest, -Diminished social drive, -Diminished sense of purpose;  2. Emotional Expression: -Restricted affect, Diminished emotional range, -Poverty of speech. |
| Kirkpatrick et al., 1989 [75] | N/A | III | ICC | SDS | 6 items | 40 SCZ | Item-item correlation between “Restricted affect” and “Poverty of speech” (r=0.84; other items ranged 0.48-0.68) |
| Galderisi et al., 2013 [76] | EFA | II | PCA | SDS | 6 items | 51 DS  44 NDS | Two-factor model at baseline and at 5-year follow-up:  1. Avolition:  -Curbing of interest, -Diminished social drive, -Diminished sense of purpose;  2. Emotional Expression:  -Restricted affect, -Diminished emotional range, -Poverty of speech. |
| Peralta et al., 2014 [77] | EFA | III | PCA | SDS | 6 items | 20 DS | Two factor model:  1. Avolition:  -Curbing of interest, -Diminished social drive, -Diminished sense of purpose;  2. Emotional Expression: -Restricted affect, -Diminished emotional range, -Poverty of speech. |
| Horan et al., 2011 [78] | EFA | II | PAF | CAINS | 23 items (final version 16 items) | 281 SCZ and schizoaffective | Two-factor model:  1. Experential: -Anhedonia: social frequency, -Avolition: social, -Anhedonia: recreation frequency, Anhedonia: physical frequency, -Anhedonia: Social intensity  -Avolition: recreation, -Anhedonia: social expected intensity, -Asociality: friendship, -Anhedonia: physical intensity, -Asociality: family, -Anhedonia: recreation intensity, -Avolition: vocational, -Anhedonia: recreational expected intensity, -Anhedonia: physical expected intensity, -Asociality: romantic, -Avolition: self-car;  2. Expressive: -Alogia: spontaneous, -Alogia: quantity, -Blunted: gestures, -Blunted: vocal, -Blunted: facial, -Blunted: spontaneous movement,  -Blunted: eye contact.  Hierarchical model confirmed the EFA results |
| Kring et al., 2013 [79] | EFA | II | PAF | CAINS | 16 items (final version 13 items) | 162 SCZ and schizoaffective | Two-factor model:  1. Expression: -Expression: vocal prosody, -Expression: facial, -Expression: gestures, -Expression: speech;  2. Motivation/Pleasure: -Social: expected pleasure, -Recreation: expected pleasure, -Recreation: past-week pleasure, -Social: past-week pleasure, -Recreation: motivation, -Vocational: expected pleasure, -Social: family relationships, -Social: friendships, -Vocational: motivation. |
| Engel et al., 2014[80] | EFA | III | PAF | German version CAINS | 13 items | 53 SCZ and schizoaffective | Two-factor model:  1. Motivation and pleasure: -Social: expected pleasure, -Recreation: expected pleasure, -Recreation: past-week pleasure, -Social: past-week pleasure, -Recreation: motivation, -Vocational: expected pleasure, -Social: family relationships, -Social: friendships, -Vocational: motivation;  2. Expression: -Expression: vocal prosody, -Expression: facial, -Expression: gestures, -Expression: speech. |
| Blanchard et al., 2017 [81] | EFA | II | PAF | CAINS | 13 items | 501 SCZ | Two-factor model:  1. Motivation and pleasure: -Social: expected pleasure, -Recreation: expected pleasure, -Recreation: past-week pleasure, -Social: past-week pleasure, -Recreation: motivation, -Vocational: expected pleasure, -Social: family relationships, -Social: friendships, -Vocational: motivation;  2. Expression: -Expression: vocal prosody, -Expression: facial, -Expression: gestures, -Expression: speech. |
| Rekhi et al., 2019 [82] | EFA;  CFA | II | χ2 value and the comparative fit index;  Models tested:  2-factor model  4-factor model | CAINS | 13 items | 274 SCZ | Four-factor model:  1. MAP social: -Social: expected pleasure, -Social: past-week pleasure, -Social: family relationships, -Social: friendships;  2. MAP vocational: -Vocational: expected pleasure, -Vocational: motivation;  3. MAP recreational: -Recreation: expected pleasure, -Recreation: past-week pleasure, -Recreation: motivation;  4. MAP expression: -Expression: vocal prosody, -Expression: facial, -Expression: gestures, -Expression: speech. |
| Kirkpatrick et al., 2011 [83] | EFA | III | PCA | BNSS | 13 items | 20 SCZ | Two-factor model:  1. Anhedonia, Asociality, Avolition: -Intensity of pleasure during activities, -Frequency of pleasure during activities, -Intensity of expected pleasure from future activities, -Distress, -Asociality behavior, -Asociality inner experience, -Avolition behavior, -Avolition inner experience;  2. Blunted affect and alogia: -Facial expression, -Vocal expression, -Expressive gestures, -Quantity of speech, -Spontaneous elaboration. |
| Strauss et al., 2012 [84] | EFA | II | PCA | BNSS | 13 items | 146 SCZ and schizoaffective | Two-factor model:  1. Motivation and pleasure: -Intensity of pleasure during activities, -Frequency of pleasure during activities, -Intensity of expected pleasure from future activities,  -Asociality behavior, -Asociality inner experience, -Avolition behavior, -Avolition inner experience;  2. Emotional expressivity: -Facial expression, -Vocal expression, -Expressive gestures, -Quantity of speech, -Spontaneous elaboration -Lack of normal distress. |
| Mucci et al., 2015 [85] | EFA | II | PAF | BNSS | 13 items | 912 SCZ | Two-factor model:  1. Avolition: -Intensity of pleasure during activities, -Frequency of pleasure during activities, -Intensity of expected pleasure from future activities,  -Asociality behavior, -Asociality inner experience, -Avolition behavior, -Avolition inner experience;  2. Poor Emotional Expression: -Facial expression, -Vocal expression, -Expressive gestures, -Quantity of speech, -Spontaneous elaboration, -Distress. |
| Garcia-Portilla et al., 2015 [86] | EFA | II | PCA | BNSS | 13 items | 190 SCZ | Three-factor model:  1. External world: anhedonia and asociality: -Pleasure: intensity, -Pleasure: frequency, - Expected pleasure: intensity, -Asociality behavior, -Asociality inner experience;  2. Inner world: Avolition and blunted affect: -Avolition behavior, -Avolition inner experience; -Facial expression, -Vocal expression, -Expressive gestures;  3. Alogia: -Quantity of speech, -Spontaneous elaboration. |
| de Meideros et al., 2018 [87] | EFA | II | Correlation analysis | BNSS-brazilian version | 13 items | 111 SCZ | Two-factor model:  1. Motivation/pleasure:  -Anhedonia (Intensity of pleasure during activities, Frequency of pleasure during activities, Intensity of expected pleasure from future activities); -Asociality (Asociality behavior, Asociality inner experience); -Avolition (Avolition behavior, Avolition inner experience);  2. Emotional expressivity: -Blunted Affect (Facial expression, Vocal expression, Expressive gestures); -Alogia (Quantity of speech, Spontaneous elaboration). |
| Ahmed et al., 2019 [88] | EFA;  CFA | II | χ2 value and the comparative fit index;  Models tested:  1-factor model  2-factor model  5-factor model  Hierarchical model | BNSS | 13 items | 1691 psychotic patients | Five-factor model  1. Anhedonia: -Intensity of pleasure during activities, -Frequency of pleasure during activities, -Intensity of expected pleasure from future activities;  2. Asociality: -Asociality behavior, -Asociality inner experience;  3. Avolition: -Avolition behavior, -Avolition inner experience;  4. Blunted affect: -Facial expression, -Vocal expression, -Expressive gestures;  5. Alogia: -Quantity of speech, -Spontaneous elaboration.  The “distress” might represent a six factor;  Good fit- Hierarchical model: 5 first order factors and two order factors (motivation/pleasure and diminished expressivity) |
| Strauss et al., 2018 [89] | CFA | II | χ2 value and the comparative fit index;  Models tested:  1-factor model  2-factor model  5-factor model  Hierarchical model | SANS;  BNSS;  CAINS |  | 860 SCZ | Best fit- Five-factor model:  1. Anhedonia; 2. Asociality; 3. Avolition; 4. Blunted affect; 5. Alogia.  Good fit- Hierarchical model: 5 first order factors and two order factors (motivation/pleasure and diminished expressivity) |
| Strauss et al., 2018 [90] | Network analysis | II | Network analysis | BNSS | 13 items | Study 1:201  Study 2: 912 | Five-factor model and six factor model  1. Anhedonia; 2. Asociality; 3. Avolition; 4. Blunted affect; 5. Alogia;  The “distress” might represent a six factor |
| Mucci et al., 2019 [91] | CFA | II | χ2 value and the comparative fit index;  Models tested:  1-factor model  2-factor model  5-factor model  Hierarchical model | BNSS | 13 items | 249 SCZ | Best fit- five-factor model:  1. Anhedonia: -Intensity of pleasure during activities, -Frequency of pleasure during activities, -Intensity of expected pleasure from future activities;  2. Asociality: -Asociality behavior, -Asociality inner experience;  3. Avolition: -Avolition behavior, -Avolition inner experience;  4. Blunted affect: -Facial expression, -Vocal expression, -Expressive gestures;  5. Alogia: -Quantity of speech, -Spontaneous elaboration.  Good fit-Hierarchical model: 5 first order factors and two order factors (motivation/pleasure and diminished expressivity) |

CFA: Confirmatory Factor Analysis; PAF: Principal Axis Factoring; BNSS: Brief Negative Symptom Scale; SCZ: subjects with schizophrenia; EFA: Exploratory Factor Analysis; PCA: Principal Component Analysis; LISREL: Linear Structural Relations; SANS: Scale for the Assessment of Negative Symptoms; BPRS: Brief Psychiatric Rating Scale; SAPS: Scale for the Assessment of Positive Symptoms; PANSS: Positive and Negative Syndrome Scale; SDS: Schedule for Deficit Syndrome; DS; Deficit Schizophrenia subjects; NDS: Non- Deficit Schizophrenia subjects; CAINS: Clinical Assessment Interview for Negative Symptoms; MAP: Motivation and pleasure; NSA: Negative Symptoms Assessment.

**eTable 4. Evaluation of the 5 negative symptom dimensions in 17 scales.**

|  | **Alogia** | **Social withdrawal** | **Blunted affect** | **Avolition** | **Anhedonia** |
| --- | --- | --- | --- | --- | --- |
| SSRAWS (Venables, 1957) [92] | x | x |  | x | x |
| BPRS anergy factor (Overall et al., 1967) [93] |  |  | x |  |  |
| EBS (Abrams and Taylor, 1978) [94] | x | x | x | x |  |
| NSRS (Iager et al., 1985) [95] | x | x | x | x |  |
| Negative PANSS sub-scale (Kay et al., 1989) [96] | x | x | x |  |  |
| SEDS (Liddle and Barnes, 1988) [97] |  |  | x | x |  |
| SANS (Andreasen, 1989) [98] | x | x | x | x | ^1^ |
| SDS (Kirkpatrick, 1989) [75] | x | x | x | x | ^2^ |
| NSA-16 (Alphs et al., 1989) [99] | x | x | x | x | ^2^ |
| HEN (Mortimer et al., 1989; Mortimer et al., 1989)[100, 101] | x | x | x |  | x |
| SDSS (Jaeger et al., 1990) [102] |  |  |  | x | x |
| SENS (Selten et al., 1993) [103] | x | x | x | x | ^1^ |
| MASS (Tremeau et al., 2008) [104] | x | x | x |  |  |
| BNSS (Kirkpatrick et al., 2011) [83] | x | x | x | x | x |
| CAINS (Kring et al., 2013) [79] | x | ^3^ | x | x | x |
| MAP-SR (Llerena et al., 2013) [105] |  | x |  | x | x |
| SNS (Dollfus et al., 2016) [106] | x | x | x | x | x |

BNSS : Brief Negative Symptoms Scale; CAINS: Clinical Assessment Interview for Negative Symptoms; EBS : Rating Scale for Emotional Blunting; HEN : High Royds Evaluation of Negativity Scale; MAP-SR : The Motivation and Pleasure Scale-Self Report; MASS : Motor Affective Social Scale; NSA-16 : Negative Symptoms Assessment 16 NSRS: Negative Symptoms Rating Scale; SANS : Scale for Assessment of Negative Symptoms; SDS : Schedule for the Deficit Syndrome; SDSS : subjective deficit syndrome scale; SEDS : Subjective Experience of Deficits in Schizophrenia; SENS : subjective experience of negative symptoms; SNS : Self-assessment of Negative Symptoms; SSRAWS : Short Scale for Rating Activity-Withdrawal in Schizophrenics;

^1^ no specific item for assessing anhedonia which is evaluated with asociality

^2^ no specific item for assessing anhedonia which is evaluated with reduced emotional range

^3^ no specific item for assessing asociality which is evaluated with other symptoms like avolition and anhedonia

**eTable 5. List of included reviews, their methods and process of analysis, the main results and comments including evidence ratings on negative symptoms in schizophrenia.**

| **Study** | **Type of study/level of evidence** | **Assessment Instruments** | **Literature or sample size and type** | **Results /Comments** |
| --- | --- | --- | --- | --- |
| Blanchard et al., 2011 [107] | Unsystematic Review study  Level III | Overview of first-generation scales on NS (SANS, PANSS, NSA) | Andreasen et al., 1989 [98]; Kay et al., 1987 [108]; Alphs et al., 1989 [99]; Forbes et al., 2010 [109] | Limitations of first-generation scales and presentation of the data-driven iterative process for the development of CAINS |
| Daniel et al., 2011 [110] | Research study,  Level III | PANSS negative, Marder negative factor, NSA-16 | Two international trials in North America, Western Europe, Eastern Europe, South/Central America, and Australia and South Africa | Despite cultural and linguistic differences among raters, standardizing measurement of negative symptoms in international clinical trials is possible using available NSA-16, PANSS negative subscale, and Marder negative subscale |
| Axelrod et al., 1993 [111] | National Multicenter Research study,  Level II | NSA-16 | Study 1: 223 schizophrenic inpatients  Study 2: 276 schizophrenic inpatients | Good internal consistency and five-factor model (CFA) (Communication, Emotion/Affect, Social Involvement, Motivation, and Retardation) found in both studies.  Convergent and discriminant validity not tested |
| Velligan et al., 2009 [112] | Research study/Level II | NSA-16 | 125 stable outpatients with SZ assessed at 6-month interval | Negative symptoms drove the changes in SOFAS rather than the reverse.  Moderate correlations between negative symptoms and functional outcomes |
| Alphs et al., 2011 [113] | Research study,  Level II | Comparison of NSA-4 and NSA-16 | 561 patients with predominant negative symptoms | NSA‐4: high correlation and high degree of overlap with NSA‐16.  Good convergent (PANSS negative, PANSS Marder factor) and divergent (CDSS, PANSS factors) validity, internal consistency, test-retest reliability for both scales |
| Kirkpatrick et al., 2011 [83] | Research study/ Level III | BNSS | 20 patients with SZ (DSM-IV) | Good interrater reliability and internal consistency for the total scales and the 6 subscales.  No benefit from excluding any items of BNSS (including Distress item)  Good test-retest reliability for the BNSS and the subscales.  Good concurrent (SANS, PANSS negative subscale) and discriminant (PANSS positive subscale, PANSS depression item, cognition) validities.  Distress item measures the absence of dysphoria.  PCA: 2-factors (anhedonia/avolition/asociality and emotional expressivity) |
| Strauss et al., 2012 [114] | Research study/level II | BNSS | 100 stabilized outpatients with SZ (n=88) or schizoaffective disorder (n=12) (DSM-IV)  (24 deficit Vs. 76 non deficit (SDS) | Good psychometric properties: good internal consistency, stability measure, discriminant validity (BPRS positive disorganized and total scores). Good convergent validity (BPRS negative, SANS, LOF).  No correlation between BNSS or Anhedonia subscale and BPRS depression.  BNSS Distress item negatively correlated with the sum BPRS Depression, Guilt, Anxiety, and Hostility items.  BNSS anhedonia subscale correlated with Chapman PA or SA.  BNSS correlated with the MCCB total t-score, the domain scores for Processing Speed, Attention/Vigilance, and Working Memory. |
| Mané et al., 2014 [115] | Research study/level III | BNSS (Spanish) | 20 SZ patients (DSM-IV) | Strong inter-rater, test-retest and internal consistency properties.  Good convergent (SANS, PANSS negative) and discriminant (PANSS positive) validity. |
| Mucci et al., 2015 [85] | Multicenter Research study/level II | BNSS (Italian) | 912 stabilized outpatients with SZ (DSM-IV) | Excellent inter-rater reliability.  Good convergent validity (PANSS negative). Weak correlation with PANSS positive (r=0.26) and CDSS (r=0.28).  BNSS anhedonia weakly correlated with CDSS (r=0.27).  PAF: 2 factors EE and MAP with distress item loading on MAP. |
| Bischof et al., 2016 [116] | Research study,  Level II | BNSS (German) | 65 patients with SZ, 10 with SAD (DSM-IV) | Good internal consistency, inter-rater reliability, convergent validity (SANS, PANSS negative, GAF, PSP, TEPS), discriminant validity (PANSS positive, depressed factor, excited factor, CDSS, MSAS)  Moderate correlation between BNSS distress item and PANSS depressed factor. |
| Polat Nazlı et al., 2016 [117] | Research study/ Level III | BNSS (Turkish) | 75 SZ patients | Good internal consistency.  BNSS correlated with PANSS Total Score, Positive Symptoms Subscale, Negative Symptoms Subscale, and General Psychopathology Subscale. CDSS and ESRS were not correlated with BNSS. The factor structure of the scale consisted on the same items as in the original version. |
| Strauss et al., 2016 [118] | Research study,  Level II | BNSS | 46 outpatients with BD, 50 with SZ (DSM-IV-TR), and 27 controls | Good Internal consistency and convergent validity (BPRS negative) in the 3 groups.  In controls, BNSS distress was at 0.  SZ and BD only differed on blunted affect and alogia items, not on anhedonia, avolition, or asociality.  WAIS-III not associated with BNSS total, AA, or EE scores in BD or SZ, except for 1 correlation between BNSS AA and Block Design in BD. In controls, greater severity of BNSS total and AA scores was associated with poorer Digit Symbol Coding performance. |
| de Medeiros et al., 2018 [87] | Multicenter Research study/level III | BNSS (Brazilian) | 111 outpatients with SZ (DSM-5) | Excellent internal consistency, inter-rater reliability. Good convergent (Marder negative PANSS) and discriminant validity (PANSS positive).  PAF: 2 factors, namely, MAP and EE for 68.63% of the total variance (with MAP: 55.8% of variance); BNSS Distress item poorly loads on MAP (0.34) |
| Kirkpatrick et al., 2018 [119] | Research study/level II | BNSS | A double-blind study with 2 active arms (low Vs. high doses) and a placebo arm in 245 patients with SZ (DSM-5) | Sensitivity to change of BNSS total score and both factor scores (AAA and EXP) |
| Ahmed et al., 2019 [88] | Follow-up Research study,  Level II | BNSS | 1678 patients (Italy, Spain, China, Switzerland, US) with SZ or SAD (DSM-IV) | Cross-Cultural validation of the five-factor structure of negative symptoms.  5-factor and hierarchical models provided excellent fit. The 5-factor model outperformed the hierarchical model. |
| Ang et al., 2019 [120] | Research study,  Level II | BNSS (English version) | 274 English speaking Asian outpatients with SZ (DSM-IV-TR) | Validation of BNSS in Asian population.  Good internal consistency (13 items, subscales, MAP and EXP with distress item removed), concurrent and validity (SANS, PANSS negative) and discriminant validity (SAS, CDSS, SANS attention, SARS, PANSS positive). BNSS Distress modestly correlated with, PANSS Depression and PANSS Excitement, EE and MAP but low correlated with other negative symptoms.  CFA: The five-factor model fit the data better than the two-factor model. A second-order model was superior to both models (with distress item removed).  Good predictive validity (More severe symptoms on BNSS Total, MAP, Avolition and Asociality associated with functioning (lower GAF)) |
| Gehr et al., 2019 [121] | Research study/  Level III | BNSS (Danish) | 49 Acutely and chronically patients with SZ or schizoaffective disorder | Good internal consistency and convergent validity.  Discriminant validity compromised by correlations with positive symptoms and Parkinsonism, may be due to a high level of secondary negative symptoms related to acute patients included |
| Mucci et al., 2019 [91] | European Multicenter Research study/level II | BNSS (10 countries) | 249 patients with SZ (DSM-IV) | Excellent internal consistency, convergent (PANSS negative) and discriminant validity (PANSS positive, CDSS).  CFA: 5-factor model and the hierarchical model provided the best fit, with a small advantage of the 5-factor fit.  BNSS-avolition explained 23.9% of psychosocial functioning (PSP) |
| Strauss et al., 2019 [60] | Research study,  Level II | BNSS | Re-analysis of 2 samples of patients with SZ and SAD (DSM-IV) (201 out-patients form US; 212 patients from Italy) | Network analysis to evaluate the latent structure indicated that the 13 BNSS items divided into 6 negative symptoms domains consisting of anhedonia, avolition, asociality, blunted affect, alogia, and lack of normal distress. |
| Treen et al., 2019 [122] | Multicenter European Research study,  Level II | BNSS | 80 patients with SZ or schizophreniform disorder (ICD-10) | Lack of statistical prediction of the CDSS and PANSS-P on the total BNSS.  Marginal influence of anhedonia and avolition on the CDSS |
| Wojciak et al., 2019 [123] | Research study,  Level III | BNSS (Polish) | 40 patients with paranoid SZ (ICD-10) | Good internal consistency, good convergent validity (PANSS negative) |
| Tatsumi et al., 2020 [124] | Narrative review/level III | BNSS | Ahmed et al., 2019 [88], Ang et al., 2019 [120], Strauss et al., 2012 [114], Strauss et al., 2016 [118], Strauss et al., 2019 [60] , Mané et al., 2014 [115], Mucci et al., 2015 [85], Bischof et al., 2016 [116], de Medeiros et al., 2019 [125], Gehr et al., 2019 [121], Wójciak et al., 2019 [123], Polat Nazli et al., 2016 [117] | Review of studies published since 2010. Eleven published cross-cultural validation studies with strong psychometric properties, similar to the original English version. Good internal consistency, inter-rater reliability, good convergent and discriminant validity, sensitive to drug effects, with 5-factor structure. |
| Forbes et al., 2010 [109] | Research study/  Level III | CAINS-beta (23 items) | 37 outpatients with SZ  or schizoaffective disorder (DSM-IV) | Results on interrater agreement, convergent and discriminant validity led to the subsequent version (CAINS-beta2) |
| Horan et al., 2011 [78] | Research study/  Level II | CAINS-beta2 (23 items) | 281 people with SZ (n=223) or schizoaffective disorder (n=58) | Exploratory factor analyses: 2 factors (asociality-avolition-anhedonia and affective blunting-alogia).  Good interrater agreement, convergent and discriminant validity.  Results guided item modification or deletion |
| Kring et al., 2013 [79] | Research study/level II | CAINS (13 items) | 162 Outpatients With SZ (N=139) or Schizoaffective Disorder (N=23) ((SANS, PANSS negative subscale) (DSM-IV) | Good interrater agreement, test-retest reliability, internal consistency.  No gender difference.  Racial or ethnic difference on the expression subscale.  PCA: 2 factors (expression (four items reflecting diminished outward expression and speech) and motivation/pleasure (nine items reflecting diminished motivation, pleasure, and social engagement).  Good convergent validities of CAINS subscales (SANS, TEPS, SAS, SCS, FACES, BAS, BIS).  Good divergent validities of CAINS subscales (BPRS depression, CDSS, SARS and cognition).  The CAINS motivation/pleasure scale was modestly related to positive symptoms and agitation.  Motivation/pleasure scale was related to social, family, independent living, and vocational functioning, while The expression scale was related to independent living and family functioning. |
| Engel et al., 2014 [80] | Research study,  Level II | CAINS (German) | 53 In- and outpatients with SZ or schizoaffective disorder (DSM-IV) | Validation of both subscales of CAINS  High internal consistency and inter-rater agreement.  Two-factor solution (EXP and motivation/pleasure)  Convergent validity (negative PANSS, consummatory pleasure TEPS).  Discriminant validity (positive PANSS, BDI, general PANSS)  CAINS motivation/pleasure subscale (but not CAINS EXP) negatively correlated with the global assessment of functioning (GAF). |
| Chan et al., 2015 [126] | Research study,  Level II | CAINS (Chinese in both Mandarin and Cantonese) | 68 patients with SZ (DSM-IV) | Validation of both subscales with PCA (2 factors (expression and motivation/pleasure), good internal consistency, convergent (SANS, negative PANSS) and discriminant (SAPS, positive PANSS) validity; the factor ‘motivation/pleasure’ correlates with the ‘emotional suppression’ subscore of EES.  Both factors correlate with social function (SOFAS) |
| Valiente-Gómez et al., 2015 [127] | Research study,  Level II | CAINS (Spanish) | [Outpatients](https://www.sciencedirect.com/topics/medicine-and-dentistry/outpatient) and inpatients (N = 100) with [DSM-IV](https://www.sciencedirect.com/topics/medicine-and-dentistry/dsm-iv) SZ | Good inter-rater and intra-rater reliability. Good convergent validity (CAINS overall and both subscales correlated with SANS and negative [PANSS](https://www.sciencedirect.com/topics/medicine-and-dentistry/positive-and-negative-syndrome-scale)); Significant correlations with positive symptoms and general psychopathology reduced and mostly became insignificant when overall severity of illness was controlled for. Significant correlations with depression disappeared when severity was controlled for. Correlation between CAINS-Exp subscale and Parkinsonism. Factor analysis: a two-dimensional structure |
| Jung et al., 2016 [128] | Research study/  Level II | CAINS (Korean) | Inpatients (n=49) and outpatients (n=70) with SZ | Validation of both subscales of CAINS Korean version  CFA: 2 subscales of 9 items related to “motivation/pleasure” and 4 items related to “expression”  Good inter-rater reliability, test-retest reliability  Convergent (negative PANSS, SANS) Discriminant validity (positive PANSS, BDI, CDSS, MSAS. |
| Blanchard et al., 2017 [81] | Multicenter (n=15) research study  Level II | CAINS | 501 patients with SZ, SAD, or schizophreniform disorder (DSM-IV-TR) | Validation of both subscales (MAP and EXP)  PCA: 2 factors (MAP and EXP)  For both subscales: high internal consistency, good convergent validity (NSA-4, PANSS negative); significant correlation with functioning (PSP; higher for MAP), good temporal stability; weak correlation with cognitive impairment  MAP correlated with quality of life (SQLS), modestly correlated with positive symptoms, depression, disorganization, and excitement (PANSS)  African-American participants had higher MAP scores than white participants |
| Jang et al., 2017 [129] | Research study/  Level II | CAINS (Korean) | 180 patients with schizophrenia | High internal consistency and inter-rater reliability; adequate convergent validity (BPRS negative, BAS), divergent validity (BPRS, CDSS, neurocognitive tasks)  EFA: 2 factors (motivation/pleasure and expression deficit dimensions) |
| Xie et al., 2018 [130] | Research study,  Level II | CAINS (Chinese) | 185 patients with SZ (DSM-IV)  43 nonpsychotic first-degree relatives  44 healthy controls  37 young people with social anhedonia  36 young healthy volunteers without any family history of psychosis | Validation of both subscales by CFA: 2 factors (motivation/pleasure and expression); Good convergent (SANS, negative PANSS) and discriminant validity (motivation/pleasure inversely correlated with consummatory pleasure subscales of ACIPS and TEPS).  No correlation of both subscales with EES.  Good interrater agreement and test-retest reliability.  Good discriminant validity in differentiating negative symptoms in people with SZ, nonpsychotic first-degree relatives and people with social anhedonia. |
| Llerena et al., 2013 [105] | Research study/level III | 18-items and revised 15-item MAP-SR | Patients with SZ (n= 33) or schizoaffective disorder (n= 4) | 15-item MAP-SR: good internal consistency and convergent validity (Motivation and Pleasure scale of the CAINS), discriminant validity, with little association with psychotic symptoms ordepression/anxiety. MAP-SR scores related to social anhedonia, social closeness, and clinician-rated social functioning, and moderately correlated with the Agitation/Mania subscale of the BPRS |
| Engel et al., 2016 [131] | Research study,  Level II | MAP-RS (German) | 50 in- or out-participants with acute or remitted SZ or schizoaffective disorder | High internal consistency; Convergent validity (experience sub-scale of the CAINS and PANSS negative); discriminant validity (PANSS positive and general) but correlated moderately with depression (BDI-II) and only trend correlation with global functioning (GAF) |
| Kim et al., 2016 [132] | Multicentric Research study/  Level II | MAP-RS (Korean) | 139 outpatients with SZ (DSM-IV) | Good internal consistency, convergent validity (MAP subscale of CAINS, anhedonia-avolition subscales of SANS), and divergent validity (positive symptoms, depression/anxiety, Agitation/mania subscales of BPRS and CDSS) |
| Engel et al., 2017 [133] | Research study,  Level III | MAP-RS (German) | 70 HC and 50 in- or outpatients with acute or remitted SZ (n=39) or schizoaffective disorder  (n=11). | 31.4% of the healthy individuals and 14% of the patients rated their negative symptoms as more severe, whereas 15.7% of the healthy individuals and 40% of the patients rated them as less  severe than the observers |
| Dollfus et al., 2016 [106] | Research Study,  Level II | SNS | 49 stabilized patients with SZ and SAD (DSM-5) | Good internal consistency, convergent validity (SANS), discriminant validity (insight, positive symptoms, parkinsonism).  Excellent intra-subject reliability.  PCA on 5 subscores : 2 factors (apathy and emotional).  Correlation between SNS and CDSS scores but not between ‘emotional component’ and CDSS |
| Dollfus et al., 2019 [134] | Research study,  Level II | SNS | Comparative study: 109 patients with SZ and SAD Vs. 99 healthy controls | Appropriate screening tool for distinguishing between SZ and HC with a threshold value of 7 in this population and regardless the level of depressive and negative symptoms |
| Rodriguez-Testal et al., 2019 [135] | Research study/level II | SNS (Spanish) | 4521 adolescents (53.6% female) from 11 to 18 years of age | Confirmatory Factor Analysis of the SNS. Confirmed an internal structure of five first-order factors. SNS was invariant across sex and age. Relationships were found strong  between the SNS with depressive symptomatology, moderate with ideas of reference and low with aberrant salience.  Interest of SNS in screening NS in academic orientation and in healthcare. |
| Dollfus et al., 2020 [136] | Narrative review including a research report on SNS,  Level III | Self-assessments of NS  SNS | 245 patients with SZ and SAD (DSM-5) | PCA on SNS confirmed an internal structure of five first-order factors supporting the 5 domains of NS.  Scales review on negative symptoms |
| Garcia-Portilla et al., 2015 [137] | Narrative review/Level III | BPRS, SANS, SENS, PANSS, BNSS, CAINS, MAP-SR, NSA | Kring et al., 2013 [79], Kirkpatrick et al., 2011 [83], Overall et al., 1967 [93], Andreasen et al., 1989 [98]; Selten et al., 1993 [103], Kay et al., 1987 [108], Llerena et al., 2013 [105], Alphs et al., 1989 [99] | The BPRS, SANS, the SENS and the PANSS belong to the first generation, while the BNSS, the CAINS and the MAP-SR belong to the second generation. The NSA can be considered a transitional instrument between the two |
| Strauss et al., 2016 [118] | Research study,  Level II | Comparison CAINS and BNSS | 65 outpatients diagnosed with SZ or schizoaffective disorder (DSM-IV) | Both scales have good psychometric properties: good internal consistency, convergent validity (SANS, BPRS negative, LOF whatever the subscales and DPB), and discriminant validity (BPRS positive and depression). Correlations between MCBB and BNSS EXP and MAP. Correlations between BNSS MAP and Chapman SA.  High correspondence between CAINS and BNSS blunted affect and alogia items.  But, moderate convergence for avolition and asociality items, and low convergence among anhedonia items.  So, important distinctions among the items related to motivation and pleasure. |
| Kumari et al., 2017 [138] | Narrative review/Level IV | PANSS, SANS, NSA-16, NSA-4, CAINS, BNSS | Obermeier et al., 2011 [139], Alphs et al., 1989 [99], Axelrod et al., 1993 [111], Strauss and Gold, 2016 [118], Kring et al., 2013 [79], Daniel et al., 2011 [110] | The older scales do not incorporate the latest research on negative symptoms. CAINS and BNSS are attractive for both their reliability and their concise accessible format.  On debate: to incorporate in a future scale a multidimensional model of SZ and addresses the psychosocial and cognitive components. |
| Galderisi et al., 2018 [9] | Review/Level III | Comparison of the scales on NS: PANSS, SANS, BNSS, CAINS | Kirkpatrick et al., 2011 [83], Kring et al., 2013 [79], Lincoln et al., 2016 [140]; Marder and Kirkpatrick, 2014 [141] | Report on limitations of PANSS and SANS; items comparison of CAINS and BNSS on the assessment of MAP and EXP |
| Strauss et al., 2018 [89] | Research study,  Level II | SANS, BNSS, CAINS | Re-analysis SANS (n = 268), 26 Brief Negative Symptom Scale (BNSS)30 (n = 192), and Clinical Assessment Inventory for Negative Symptoms (CAINS)17 (n = 400). | 4 CFA models tested (unidimensional, 2-dimensionnal, 5-factor model, hierarchical model with 2 second-order factors (EXP and MAP) and 5 first (factor models.  Across the 3 scales: Excellent fit for the 2 last models with the 5-factor models being the best. |
| Strauss et al., 2018 [60] | Descriptive study/Level III | BNSS, CAINS, SANS | Kimhy et al., 2006 [73], Nakaya and Ohmori 2008 [74], Horan et al., 2011 [78], Kring et al., 2013 [79], Peralta and Cuesta, 1995 [65], Alphs et al., 1989 [99], Overall et al.,1967 [93], Strauss and Gold, 2016 [118], Ahmed et al., 2019 [88] | A Review of Evidence Supporting the 5 Consensus Domains |
| Richter et al., 2019 [142] | Research study/ Level III | CAINS and  MAP-RS | 21 participants with SZ, 22 participants with MDE and 25 HC | The ratings of depressive symptoms (HAMD-17, BDI) and rater assessment of negative symptoms (CAINS) – specifically sub scale expressive deficits – managed to discriminate between subjects with SZ and those with MDE; MAP-SR did not. |
| Wojciak et al., 2019 [123] | Research study,  Level III | SNS (Polish), PANSS, BNSS | 40 patients with paranoid SZ (ICD-10) | High values of the Cronbach’s alpha  coefficient for the whole scale and for the subscales. The SNS and its subscales showed significant correlations with the total BNSS score and with the scores of the BNSS subscales. |
| Blanchard et al., 2020 [143] | Narrative review/ Level III | Overview of the first-wave measures on NS (SANS, PANSS, NSA), next generation measures (CAINS, BNSS), self-assessment (MAP-SR, SNS) and laboratory measures of NS | Andreasen et al., 1989 [98]; Kay et al., 1987 [108]; Alphs et al., 1989 [99]; Blanchard et al., 2011 [107]; Horan et al., 2011 [78]; Kring et al., 2013 [79]; Kirkpatrick et al., 2011 [83]; Strauss et al., 2012 [84]: Engel et al., 2017 [133], Engel et al., 2016 [131]; Kim et al., 2016 [132], Llerena et al., 2013 [105]; Dollfus et al., 2016 [106] | Presentation of 3 first generation scales with their limitations and recent scales including self-assessment with their interest and differences  Considerations on ecological momentary assessment and on a transdiagnostic and prodrome approach |

AAA : anhedonia, avolition, and asociality subscales; ACIPS: Anticipatory and Consummatory Interpersonal Pleasure Scale; BAS: Behavioral Activation Scale; BDI: Beck depression inventory; BDI-II :Beck Depression Inventory, revision; BIS: Behavioral Inhibition Scale ; BNSS: Brief Negative Symptoms Scale; BP: Bipolar disorder; BPRS: Brief Psychiatric Rating Scale; Chapman SA : Chapman Social Anhedonia Scale; CFA: confirmative factor analysis; CHR : Clinical High-Risk for psychosis; CDSS: Calgary Depression Scale for Schizophrenia; CFA: Confirmatory Factor Analysis; CAINS: Clinical Assessment Interview for Negative Symptoms; DPB: Defeatist Performance Beliefs Scale; EXP: expressivity (blunted affect and alogia subscales); EES : Emotional Expressivity Scale; FACES: The Facial Expression Coding System; GAF: Global Assessment of Functioning; HC: healthy controls; LOF: Level of Function Scale; MCCB : MATRICS Consensus Cognitive Battery; MAP: Motivation/pleasure; MDE : major depressive episode; MSAS: Modified Simpson Angus scale; NC: Normal control; PCA: Principal component analysis; NS: Negative symptoms; NSA: Negative Symptom Assessment; NSA-4 : 4-item Negative Symptom Assessment; PANSS: Positive And Negative Syndrome Scale; PANSS-P: Positive subscale of Positive And Negative Syndrome Scale; PAF: principal axis factoring; PINS: Prodromal Interview of Negative Symptoms ; PSP = Personal and Social Performance; SAD: schizoaffective disorders; SANS: Scale for assessment of Negative Symptoms; SAPS: Scale for the Assessment of Positive Symptoms; SARS: Simpson-Angus Rating Scale; SAS: Social Anhedonia Scale; SZ: Schizophrenia ; SCS: Social Closeness Scale; SIPS : Structured Interview for Prodromal Syndromes; SDS : Schedule for the Deficit Syndrome; SLOF : Specific Level of Functioning Scale; SNS: Self-evaluation of Negative Symptoms; SOFAS: Social and Occupational Functioning Assessment Scale; SQLS : Schizophrenia Quality of Life Scale ; TEPS: Temporal Experience of Pleasure Scale.

**eTable 6. List of included reviews, their methods and process of analysis, the main results and comments including evidence ratings on apathy in schizophrenia.**

| **Study** | **Type of study/level of evidence** | **Assessment Instruments** | **Literature or sample size and type** | **Results /Comments** |
| --- | --- | --- | --- | --- |
| **Apathy** | | | | |
| Faerden et al., 2008 [144] | Research study/level II | AES-C | 104 patients with FEP (DSM-IV). | Factor analysis: 3 factors: Apathy, Insight and Social Contacts.  Only the Apathy factor showed satisfactory psychometric properties and acceptable convergent (apathy related items of the PANSS). |
| Clarke et al., 2011 [145] | Review/ Level III | AES | Faerden [144] | Good internal consistency, test-retest reliability and interrater reliabilities in patients with SZ. |
| Fervaha et al., 2015 [146] | Research study/level III | 5 scores from 3 instruments (AES, SANS, QLS) | 62 patients with schizophrenia | The scores from these different instruments were highly inter-correlated, and largely independent of severity of other symptom domains (e.g., depression). The findings suggest that clinical ratings scales evaluating motivational deficits are tapping into a similar underlying construct. |
| Faerden et al., 2018 [147] | Research study/level II | AES-S | 84 patients with FEP | High internal consistency.  Modest external convergent validity (PANSS negative).  Good discriminant validity (PANSS positive and disorganization).  AES-S is in concordance with clinician ratings. |
| Hartmann- Riemer et al., 2018 [148] | Review/ level III | Different effort-based decision-making paradigms | 15 studies on physical efforts and 5 studies on cognitive efforts | The review of the literature reveals numerous inconsistencies in the methods applied and the findings reported. |
| Luther et al., 2018 [149] | Meta-analysis /level III | self-reported, clinician-rated, and performance-based measures | 45 studies of people with schizophrenia. | The overall mean effect size between self-reported and clinician-rated motivation measures (r = 0.27, k = 33) was significant, positive, and approaching medium in magnitude, and the overall effect size between performance-based and clinician-rated motivation measures (r = 0.21, k = 11) was positive, significant, and small in magnitude. The overall mean effect size between self-reported and performance-based motivation measures was negligible and non-significant. |

AES: Apathy Evaluation Scale; AES-C: Apathy Evaluation Scale Clinical version ; AES-S: Apathy Evaluation Scale Self-rated; FEP: First episode psychosis ; SANS: PANSS: Positive And Negative Syndrome Scale; QLS: Quality of Life Scale; Scale for assessment of Negative Symptoms

**eTable 7. Review of negative symptoms assessments in larger-scale intervention studies in the CHR population.**

| **Study** | **Type of Study** | **Sample size** | **Intervention** | **Assessment Instruments** |
| --- | --- | --- | --- | --- |
| McGorry et al., 2002 [150] | RCT | 59 | CBT + Risperidone | SANS total score |
| Morrison et al., 2004 [151] | RCT | 58 | CBT | PANSS negative total score^a^ |
| McGlashan, 2006 [152] | RCT | 60 | Olanzapine | PANSS negative total score +  SIPS negative, total score |
| Ruhrmann et al., 2007 [153] | RCT | 124 | Amisulpride + need-focused intervention | PANSS negative total score |
| Berger et al., 2008 [154] | Open label | 103 | Lithium | SANS total score |
| Kobayashi et al., 2009 [155] | Cohort study | 36 | Aripiprazole | SIPS negative, total score |
| Amminger et al., 2010 [156] | RCT | 81 | Omega-3 | PANSS negative total score^a^ |
| Addington, et al. 2011 [157] | RCT | 51 | CBT | SIPS negative, total score |
| Bechdolf, et al. 2012 [158] | RCT | 128 | Integrated psychological intervention | PANSS negative total score |
| Van der Gaag, et al. 2012 [159] | RCT | 201 | CBT | CAARMS negative, total score^a^ |
| McGorry, et al. 2013 [160] | RCT | 193 | CBT + Risperidone/  CBT + placebo | SANS total score + 5 subscale scores |
| Washida, et al. 2013 [161] | Cohort study | 61 | Second generation antipsychotics | PANSS negative total score |
| Miklowitz, et al. 2014 [162] | RCT | 129 | Family-focused therapy | SIPS negative, total score |
| Fusar-Poli, et al. 2015 [163] | Naturalistic | 258 | CBT | CAARMS negative, total score |
| Kantrowitz, et al. 2015 [164] | RCT | 35 | D-serine | SIPS negative, total score |
| McFarlane, et al. 2015 [165] | Risk-based allocation | 337 | Family-aided assertive community treatment | SIPS negative, total score |
| Piskulic, et al. 2015 [166] | RCT | 32 | Cognitive remediation | SIPS negative, total score |
| Choi, et al. 2016 [167] | RCT | 62 | Cognitive remediation | SIPS negative, total score |
| Ising, et al. 2016 [168] | RCT | 196 | CBT + TAU | CAARMS negative, total score |
| Loewy, et al. 2016 [169] | RCT | 83 | Cognitive remediation | SIPS negative, total score |
| McGorry, et al. 2017 [170] | RCT | 304 | Omega-3 + CBCM | SANS total score + 5 subscale scores |

CAARMS: Comprehensive Assessment of At-Risk Mental States; CBT: Cognitive Behavioral Therapy; CBCM: cognitive-behavioral case management; PANSS: Positive and Negative Symptom Scales; RCT: Randomized Controlled Trial; SANS: Scale for the Assessment of Negative Symptoms; SIPS: Structured Interview for Prodromal Symptoms; TAU: Treatment As Usual.

^a^ Only report negative symptom scores at baseline

**eTable 8. Level of evidence, methods and main results of included studies on the differential diagnosis between primary and secondary negative symptoms in the clinical practice.**

| **Study** | **Type of Study/level of evidence** | **Type of differential diagnosis/Assessment Instruments** | **Evidence base (used literature or sample size of studies)** | **Results** |
| --- | --- | --- | --- | --- |
| Kirschner et al., 2017 [171] | Narrative Review /Level III | Primary negative symptoms vs secondary negative symptoms due to positive symptoms | Chen et al., 2013[172]; Czobor and Volavka, 1996 [173];  Marder and Meibach, 1994 [174]; Miller et al., 1994 a, b [175, 176]; Pickar et al., 1992 [177]; Sanger et al., 1999 [178]; Tandon et al., 1993 a, b [179, 180]; Tollefson et al., 1997 [181]; Kelley et al., 1999 [67]; | Indirect evidence has been obtained from many pharmacological trials demonstrating a correlation between positive and negative symptoms: concurrent improvement with antipsychotic treatment and concurrent worsening after medication wash-out. |
| Galderisi et al., 2018 [9] | Systematic Review with clear search criteria but no detailed summary of evidence used for this topic/Level II | Primary negative symptoms vs secondary negative symptoms due to positive symptoms | Kelley et al., 1999 [67], Kirschner et al., 2017 [171], Carpenter et al., 1985 [182] | Psychotic exacerbation can mimic the avolition–apathy domain. In routine clinical practice, it is helpful to investigate the retrospective course of negative symptoms to verify whether they worsened during acute psychotic decompensation phases and improved during periods of clinical stability. The presence of a stable level of impairment suggests primary negative symptoms. Extrapyramidal symptoms can be excluded based on review of therapy changes and course of negative symptoms. |
| Carpenter et al., 1985 [182] | Expert Opinion/Level IV | Primary negative symptoms vs secondary negative symptoms due to positive symptoms | N/A | The literature has demonstrated that negative as well as positive symptoms improve in response to neuroleptic drugs. It may be this type of psychosis-linked secondary negative symptom that accounts for the observation of improvement. |
| Moller, 2007 [183] | Expert Opinion/Level IV | Primary negative symptoms vs secondary negative symptoms due to positive symptoms | N/A | Negative symptoms can be classified as primary or secondary, mainly on the basis of chronicity versus episodic appearance temporally related to causative factors and potentially reversible with correction of those factors. |
| Pickar et al., 1992 [177] | Controlled Trial limited to one group/Level II | Primary negative symptoms vs secondary negative symptoms due to positive symptoms | 21 SCZ intolerant or resistant to APs; placebo-controlled, double-blind comparison of typical neuroleptic (fluphenazine) and clozapine treatment | Concomitant improvement of positive, negative and total BPRS scores using clozapine, in comparison with placebo and fluphenazine. |
| Tandon et al., 1993 [179] | Descriptive study/Level III | Primary negative symptoms vs secondary negative symptoms due to positive symptoms | 40 SCZ before and about 8 weeks after a trial of clozapine. | There was a significant correlation between change in positive symptoms and change in negative symptoms; as with conventional neuroleptics, negative symptoms improved concomitantly with positive symptoms during clozapine treatment. |
| Marder and Meibach, 1994 [174] | Uncontrolled blinded study/Level II | Primary negative symptoms vs secondary negative symptoms due to positive symptoms | 388 SCZ in a double-blind comparison of four different doses of risperidone (2, 6, 10, and 16 mg/day), placebo, and 20 mg/day of haloperidol | The optimal daily dose of risperidone was 6 mg (as effective as 16 mg, with incidence of extrapyramidal symptoms similar to placebo). At this dose both positive and negative symptoms improved. |
| Miller, D.D. et al., 1994 [175] | Descriptive study / Level III | Primary negative symptoms vs secondary negative symptoms due to positive symptoms/  SAPS; SANS; HRSD; Simpson-Angus Extrapyramidal | 59 SCZ underwent a three-week drug wash out. | Negative symptoms worsened significantly during the three-week drug wash-out. The changes in negative symptoms during the drug-free period were correlated with the changes in psychosis and disorganization, but not with changes in depression or extrapyramidal side effects. It was not possible to substantiate if the worsening in negative symptoms was a direct result of the worsening of positive symptoms or if they were changing simultaneously, but independent of each other |
| Flaum and Andreasen, 1995 [184] | Descriptive study/Level III | Primary negative symptoms vs secondary negative symptoms due to positive symptoms | 462 subjects with nonorganic psychotic disorders evaluated with SANS. In addition to the usual SANS ratings, raters were asked to indicate their judgment as to whether the symptom was primary, secondary, or unknown (inadequate information). | Without highly specialized training on specific research instruments or the availability of extensive longitudinal information on possible factors causing secondary negative symptoms in each patient, the distinction between primary and secondary negative symptoms can be made with modest inter- and intra-rater reliability. |
| Czobor and Volavka, 1996 [185] | Descriptive study/Level III | Primary negative symptoms vs secondary negative symptoms due to positive symptoms | 178 SCZ treated with haloperidol or placebo  (data derived from two clinical placebo-controlled,  double-blind trials, with the primary aim to examine relationships between haloperidol blood levels and clinical effects) | Results indicate that positive and negative symptom-change trajectories over time were closely associated. |
| Tollefson et al., 1997 [186] | Uncontrolled blinded trial/ Level II | Primary negative symptoms vs secondary negative symptoms due to positive symptoms | 1996 SCZ in a double-blind trial randomly assigned to treatment with olanzapine (N=1,336) or haloperidol (N=660) over 6 weeks. | Patients in the olanzapine group had a significantly greater mean improvement in the extracted BPRS total score.  The comparative changes in Positive and Negative Syndrome Scale total scores confirmed this advantage, which included both positive and negative symptom scores. |
| Sanger et al., 1999 [178] | Descriptive study/Level III | Primary negative symptoms vs secondary negative symptoms due to positive symptoms | 83 FEP (from the study of Tollefson et al., 1997) in a double blind, 6-week acute treatment study (randomly assigned to haloperidol or olanzapine) | Compared to haloperidol, olanzapine showed a statistically significantly greater reduction in the Brief Psychiatric Rating Scale (BPRS) total and negative scores and in the Positive and Negative Syndrome Scale total and positive scores. |
| Kelley et al., 1999 [67] | Descriptive study/Level III | Primary negative symptoms vs secondary negative symptoms due to positive symptoms | 93 SCZ with haloperidol treatment underwent a maximum six-week drug wash-out. | The study found two SANS-derived negative symptom factors (affective flattening and diminished motivation), which had a stable structure when the patients were both on and off medication. Regression analyses, performed during the drug wash-out, demonstrated that changes in motivation were predicted by changes in anxiety/depression and psychosis, while changes in affective flattening were predicted by changes in extrapyramidal side effects. |
| Chen et al., 2013 [172] | Descriptive study/Level III | Primary negative symptoms vs secondary negative symptoms due to positive symptoms | 399 SCZ in an open-label trial treated randomly with olanzapine, risperidone, or first-generation antipsychotics | Changes in negative and positive symptoms were positively related. |
| Kirschner et al., 2017  [171] | Narrative Review/Level III | Primary negative symptoms vs secondary negative symptoms due to parkinsonism | Peralta el al., 2000 [187]; Kelley et al., 1999 [67]; Miller et al., 1994 a[175] | Temporal relationship between negative symptoms and the start of dose increase of antipsychotic drug treatment is important in differential diagnosis. |
| Galderisi et al., 2018 [9] | Systematic Review with clear search criteria but no detailed summary of evidence used for this topic/Level III | Primary negative symptoms vs secondary negative symptoms due to parkinsonism | Peralta el al., 2014 [77] (occurrence of parkinsonism in patients with primary and persistent negative symptoms, independent of their medication status) | The retrospective or prospective assessment of the course of blunted affect and alogia with respect to changes in antipsychotic treatment (either dose or drug changes) is needed to discriminate secondary negative symptoms. |
| Carpenter et al., 1985 [182] | Expert Opinion/Level IV | Primary negative symptoms vs secondary negative symptoms due to parkinsonism |  | Although not well established on the basis of existing data, there is a potentially fundamental relationship between neuroleptic drug action and negative symptoms. |
| Moller, 2007 [183] | Expert Opinion/Level IV | Primary negative symptoms vs secondary negative symptoms due to parkinsonism | N/A | Negative symptoms can be classified as primary or secondary, mainly on the basis of chronicity versus episodic appearance temporally related to causative factors and potentially reversible with correction of those factors. |
| Miller et al., 1994 [175] | Descriptive study/Level III | Primary negative symptoms vs secondary negative symptoms due to parkinsonism /  SAPS; SANS; HRSD; Simpson-Angus Scale | 59 SCZ underwent a three-week drug wash-out. | Negative symptoms worsened significantly during the three-week drug wash-out. The changes in negative symptoms during the drug-free period were correlated with the changes in psychosis and disorganization, but not with changes in depression or extrapyramidal side effects. It was not possible to substantiate if the worsening in negative symptoms was a direct result of the worsening of positive symptoms or if they were changing simultaneously, but independent of each other |
| Johnson et al., 1994 [188] | Case study/Level IV | Primary negative symptoms vs secondary negative symptoms due to parkinsonism | 28 SCZ | While dose was positively related to overall negative symptom scores as well as specific negative symptoms, no relation was found with positive symptom scores. The discussion focused on the possibility that negative symptoms might represent medication-induced side effects and the need for further research. |
| Allan et al., 1998 [189] | Descriptive study/Level III | Primary negative symptoms vs secondary negative symptoms due to parkinsonism /  PANSS; Simpson-Angus Scale | SCZ treated with haloperidol or olanzapine | A positive correlation between EPS and PANSS negative score was detected in the haloperidol group only. |
| Kelley et al., 1999 [67] | Descriptive study/Level III | Primary negative symptoms vs secondary negative symptoms due to parkinsonism | 93 SCZ with haloperidol treatment underwent a maximum six-week drug wash-out. | The study found two SANS-derived negative symptom factors (affective flattening and diminished motivation), which had a stable structure when the patients were both on and off medication. Regression analyses, performed during the drug wash-out, demonstrated that changes in motivation were predicted by changes in anxiety/depression and psychosis, while changes in affective flattening were predicted by changes in extrapyramidal side effects. |
| Peralta el al., 2000 [187] | Descriptive study / Level III | Primary negative symptoms vs secondary negative symptoms due to parkinsonism | 47 SCZ never treated. After baseline assessment, 8 patients were treated with haloperidol, 28 with risperidone, and 11 with olanzapine. | Negative symptoms were correlated with ratings of extrapyramidal symptoms. |
| Kirschner et al., 2017 [171] | Narrative review Level III | Primary negative symptoms vs secondary negative symptoms due to depression | Lako et al., 2012 [190];  Mulholland C. et al., 2000 [191], ; Kirkpatrick, 2014 [192]; | 1. The Calgary Depression Scale for Schizophrenia (CDSS) has been shown to most accurately differentiate depressive symptoms from other symptoms of schizophrenia;  2. Guilt, hopelessness and helplessness are more characteristic of depression. |
| Galderisi et al., 2018 [9] | Systematic Review with clear search criteria but no detailed summary of evidence used for this topic/Level III | Primary negative symptoms vs secondary negative symptoms due to depression | Mulholland C. and Cooper, 2000 [191]; Buckley et al., 2009 [193] | In clinical practice, the presence of the subjective component of depressed mood and other psychological features of depression, such as hopelessness, guilt, and suicidal ideation, favor the diagnosis of depression and should be clinically assessed, whereas the presence of blunted affect is more characteristic of negative symptoms. |
| Carpenter et al., 1985 [182] | Expert Opinion/Level IV | Primary negative symptoms vs secondary negative symptoms due to depression | N/A | Episodes of depression are a common occurrence in schizophrenic patients. Negative symptoms secondary to depression respond to treatment of the depression per se. Although general efficacy is not robust, some patients respond to traditional antidepressant drugs. |
| Moller, 2007 [183] | Expert Opinion/Level IV | Primary negative symptoms vs secondary negative symptoms due to depression | N/A | In some patients, the distinction from affective symptoms is relatively easy, as several of the characteristic symptoms of depression (depressed mood, feelings of worthlessness, thoughts of death and suicide) do not correspond to negative symptoms. However, other depressive symptoms (anhedonia, apathy, avolition, psychomotor retardation) do resemble negative symptoms. Therefore, it may be difficult to distinguish between (1) schizophrenia with negative symptoms; (2) depressive-type schizoaffective disorder; and (3) schizophrenia with comorbid depressive symptoms that do not fulfill the criteria for a major depressive episode. |
| Mulholland and Cooper, 2000 [191] | Narrative review/Level III | Primary negative symptoms vs secondary negative symptoms due to depression | N/A | Prominent subjectively low mood, suggesting depression, and prominent blunting of affect, suggesting negative symptoms, are the two features which are most helpful in differentiating the two syndromes. |
| Buckley et al., 2009 [193] | Narrative review/Level III | Primary negative symptoms vs secondary negative symptoms due to depression | N/A | Depression is an important co-occurring syndrome in schizophrenia, appearing with substantial anhedonia, an absence of initiative, social isolation, as well as motor retardation |
| Krynicki et al., 2018 [194] | Systematic review/Level I | Primary negative symptoms vs secondary negative symptoms due to depression | 2210 articles were identified from EMBASE, PsychInfo and MEDLINE, and further two articles were hand-searched from references. Twenty-seven met inclusion criteria and were included in the review. | Primary evidence suggests symptoms of low mood, suicidal ideation and pessimism have more specificity for depression whereas alogia and blunted affect may have more specificity as negative symptoms. Anhedonia, anergia and avolition may be common to both. Within anhedonia, it is possible to distinguish between consummatory and anticipatory components: while the first component seems to be more specific to depression, the second seems to be more specific to schizophrenia. |
| Lako, I.M. et al., 2012 [190] | Meta-analysis/Level I | Primary negative symptoms vs secondary negative symptoms due to depression / BPRS-D; PANSS-D; HAMD; MADRS; CDSS | 48 articles | Results would recommend the use of the CDSS for the measurement of depressive symptoms in research and in daily clinical practice in patients with schizophrenia. A valid self-report instrument is to be developed for the use in clinical practice. |
| Kulhara and Chadda, 1987 [195] | Descriptive study/Level III | Primary negative symptoms vs secondary negative symptoms due to depression / SANS | 59 SCZ; 59 MDD | MDD: significantly higher scores on ratings of subjective complaints, total score, global ratings, and various items of all subscales. SCZ: significantly higher scores on global rating of alogia, poor eye contact, inappropriate affect, and blocking. |
| Addington et al., 1994 [196] | Descriptive study with extensive analyses (EFA; CFA) for CDSS validity and specificity /Level II | Primary negative symptoms vs secondary negative symptoms due to depression / Scale validity for CDSS / PANSS; Simpson Angus Scale | 150 SCZ | CDSS achieves a useful degree of separation between measures of depression, negative and extrapyramidal symptoms in SCZ |
| Lancon et al., 1999 [197] | Descriptive study/Level III | Primary negative symptoms vs secondary negative symptoms due to depression / CDSS; HDRS; MADRS; ERD; PANSS. | 95 SCZ | The results confirmed the validity of the CDSS in the evaluation of depression in schizophrenia. |
| Kollias et al., 2008 [198] | Descriptive study/Level III | Primary negative symptoms vs secondary negative symptoms due to depression / rPAS; rSAS; CDSS | 62 SCZ | Depression in schizophrenia and anhedonia may overlap, and therefore, it could be difficult to clinically differentiate them, especially in acute schizophrenia patients. Since ‘depression’ in schizophrenia is a heterogeneous concept it is of great importance to use a highly specific psychometric tool, such as the CDSS, to measure it. |
| Liu et al., 2009  [199] | Descriptive study/Level III | Primary negative symptoms vs secondary negative symptoms due to depression; Validity of CDSS / MADRS; HAM-D; PANSS-D | 101 SCZ | CDSS provides optimal assessment of depression in SCZ. |
| Schennach et al., 2012 [200] | Descriptive study with adequate analysis (EFA) to assess differential validity of CDSS in SCZ subjects/ Level II | Primary negative symptoms vs secondary negative symptoms due to depression; Validity of CDSS / PANSS; CDSS; HAM-D | 278 SCZ | Three factors for CDSS and HAM-D. The first two factors of the CDSS revealed correlations with positive, negative and general psychopathology. In contrast, multiple significant correlations were found for the HAMD-17 factors and the PANSS subscores. Compared to the HAMD-17, the CDSS is a more specific instrument to measure depressive symptoms in schizophrenia and schizophrenia spectrum disorder, especially in acutely ill patients. |
| Lako et al., 2014 [201] | Descriptive study /Level III | Primary negative symptoms vs secondary negative symptoms due to depression; Validity of QIDS-SR16 / PANSS; CDSS; QIDS-SR16 | 621 SCZ | The QIDS-SR16 may reliably assess depressive symptoms in patients with psychotic disorders, but its concurrent validity with the CDSS was rather poor in this population. It would be recommended developing a new self-report questionnaire for the assessment of depressive symptoms in patients with psychotic disorders. |
| Schennach et al., 2015  [202] | Naturalistic study/Level III | Primary negative symptoms vs secondary negative symptoms due to depression; Validity of CDSS | 278 SCZ | Depressive symptoms measured with the CDSS are a discrete symptom domain with only partial overlap with positive or negative symptoms (depressed mood, observed depression and hopelessness are the most prevalent depressive symptoms) |
| Grover et al., 2017 [203] | Descriptive study with adequate analysis (EFA) to assess differential validity of CDSS in SCZ subjects/ Level II | Primary negative symptoms vs secondary negative symptoms due to depression; Validity of CDSS / HDRS; CDSS; PANSS | 267 SCZ | CDSS items separate out into 2 factors, which are stable across different stages of illness, whereas HDRS factor structure appears to be less stable across different stages of illness. Correlation analysis suggested that rating on HDRS may be affected by positive and negative symptoms of schizophrenia, whereas CDSS do not correlate with positive and negative symptoms of schizophrenia. |
| Richter, J. et al., 2019 [204] | Descriptive study /Level III | Primary negative symptoms vs secondary negative symptoms due to depression; Validity of self-report measure of depression and negative symptoms (BDI, MAP-SR) / self-ratings (BDI, MAP-SR) and observer assessments (HAMD-17, CAINS) | 21 SCZ, 22 MDD, and 25 HC subjects | To differentiate negative symptoms and depression clinicians might look for self-reported low mood and observer-rated reduction in speech as well as in gestures and facial expression. Reduced expression and moderate levels of depression point towards a negative syndrome, whereas mostly unimpaired expression and high scores of self-reported depressive symptoms are more likely to indicate a depressive syndrome. |

N/A: Not Applicable; APs: antypsychotics; SCZ: subjects with schizophrenia; SAPS: Scale for the Assessment of Positive Symptoms; SANS: Scale for the Assessment of Negative Symptoms; HRSD: the Hamilton Rating Scale for Depression; BPRS: Brief Psychiatric Rating Scale; FEP: First-episode subjects; PANSS: Positive and Negative Syndrome Scale; HAMD-17: Hamilton Rating Scale for Depression; EPS: Extrapyramidal side effects; CDSS: The Calgary Depression Scale for Schizophrenia; MDD: subjects with Major depressive disorder; HC; Healthy controls; BDI: Beck Depression Inventory; CAINS: Clinical Assessment Interview for Negative Symptoms; MAP-SR: The Motivation and Pleasure Scale-Self Report; MADRS: Montgomery-Asberg Depression Rating Scale; ERD: Widlocher Psychomotor Retardation Scale; rPAS: Revised Physical Anhedonia Scale; rSAS: Revised Social Anhedonia Scale; QIDS-SR16: Quick Inventory of Depressive Symptoms.

**References**

1. Kirkpatrick B, Buchanan RW, Ross DE, Carpenter WT, Jr. A separate disease within the syndrome of schizophrenia. Arch Gen Psychiatry. 2001;58(2):165-71.

2. Buchanan RW. Persistent negative symptoms in schizophrenia: an overview. Schizophr Bull. 2007;33(4):1013-22.

3. Kirkpatrick B, Galderisi S. Deficit schizophrenia: an update. World Psychiatry. 2008;7(3):143-7.

4. Galderisi S, Maj M. Deficit schizophrenia: an overview of clinical, biological and treatment aspects. Eur Psychiatry. 2009;24(8):493-500.

5. Kirkpatrick B. Progress in the study of negative symptoms. Schizophr Bull. 2014;40 Suppl 2:S101-6.

6. Kirkpatrick B, Mucci A, Galderisi S. Primary, Enduring Negative Symptoms: An Update on Research. Schizophr Bull. 2017;43(4):730-6.

7. Bucci P, Galderisi S. Categorizing and assessing negative symptoms. Curr Opin Psychiatry. 2017;30(3):201-8.

8. Mucci A, Merlotti E, Ucok A, Aleman A, Galderisi S. Primary and persistent negative symptoms: Concepts, assessments and neurobiological bases. Schizophr Res. 2017;186:19-28.

9. Galderisi S, Mucci A, Buchanan RW, Arango C. Negative symptoms of schizophrenia: new developments and unanswered research questions. Lancet Psychiatry. 2018;5(8):664-77.

10. Marder SR, Alphs L, Anghelescu IG, Arango C, Barnes TR, Caers I, et al. Issues and perspectives in designing clinical trials for negative symptoms in schizophrenia. Schizophr Res. 2013;150(2-3):328-33.

11. Kay SR, Sevy S. Pyramidical Model of Schizophrenia. Schizophr Bull. 1990;16(3):537-45.

12. Bell MD, Lysaker PH, Beam-Goulet JL, Milstein RM, Lindenmayer JP. Five-Component Model of Schizophrenia: Assessing the Factorial Invariance of the Positive and Negative Syndrome Scale. Psychiatry Res. 1993;52:295-303.

13. Lindstrom E, von Knorring L. Principal component analysis of the Swedish version of the Positive and Negative Syndrome Scale for schizophrenia. Nord J Psychiatry. 1993;43:257-63.

14. Kawasaki Y, Maeda Y, Sakai N, Higashima M, Urata K, Yamaguchi N, et al. Evaluation and interpretation of symptom structures in patients with schizophrenia. Acta Psychiatr Scand. 1994;89(6):399-404.

15. Lindenmayer JP, Bernstein-Hyman R, Grochowski S. Five-factor model of schizophrenia. Initial validation. J Nerv Ment Dis. 1994;182(11):631-8.

16. Lindenmayer JP, Bernstein-Hyman R, Grochowski S. A new five factor model of schizophrenia. Psychiatr Q. 1994;65(4):299-322.

17. White L, Harvey PD, Opler L, Lindenmayer JP. Empirical assessment of the factorial structure of clinical symptoms in schizophrenia. A multisite, multimodel evaluation of the factorial structure of the Positive and Negative Syndrome Scale. The PANSS Study Group. Psychopathology. 1997;30(5):263-74.

18. Dollfus S, Everitt B. Symptom structure in schizophrenia: two-, three- or four-factor models? Psychopathology. 1998;31(3):120-30.

19. Lancon C, Aghababian V, Llorca PM, Auquier P. Factorial structure of the Positive and Negative Syndrome Scale (PANSS): a forced five-dimensional factor analysis. Acta Psychiatr Scand. 1998;98(5):369-76.

20. Nakaya M, Suwa H, Ohmori K. Latent structures underlying schizophrenic symptoms: a five-dimensional model. Schizophr Res. 1999;39(1):39-50.

21. Lykouras L, Oulis P, Psarros K, Daskalopoulou E, Botsis A, Christodoulou GN, et al. Five-factor model of schizophrenic psychopathology: how valid is it? Eur Arch Psychiatry Clin Neurosci. 2000;250(2):93-100.

22. Mass R, Schoemig T, Hitschfeld K, Wall E, Haasen C. Psychopathological syndromes of schizophrenia: evaluation of the dimensional structure of the positive and negative syndrome scale. Schizophr Bull. 2000;26(1):167-77.

23. Lancon C, Auquier P, Nayt G, Reine G. Stability of the five-factor structure of the Positive and Negative Syndrome Scale (PANSS). Schizophr Res. 2000;42(3):231-9.

24. Wolthaus JE, Dingemans PM, Schene AH, Linszen DH, Knegtering H, Holthausen EA, et al. Component structure of the positive and negative syndrome scale (PANSS) in patients with recent-onset schizophrenia and spectrum disorders. Psychopharmacology (Berl). 2000;150(4):399-403.

25. Emsley R, Rabinowitz J, Torreman M, Group R-I-EPGW. The factor structure for the Positive and Negative Syndrome Scale (PANSS) in recent-onset psychosis. Schizophr Res. 2003;61(1):47-57.

26. Fitzgerald PB, de Castella AR, Brewer K, Filia K, Collins J, Davey P, et al. A confirmatory factor analytic evaluation of the pentagonal PANSS model. Schizophr Res. 2003;61(1):97-104.

27. Lee KH, Harris AW, Loughland CM, Williams LM. The five symptom dimensions and depression in schizophrenia. Psychopathology. 2003;36(5):226-33.

28. Fresan A, De la Fuente-Sandoval C, Loyzaga C, Garcia-Anaya M, Meyenberg N, Nicolini H, et al. A forced five-dimensional factor analysis and concurrent validity of the Positive and Negative Syndrome Scale in Mexican schizophrenic patients. Schizophr Res. 2005;72(2-3):123-9.

29. van der Gaag M, Hoffman T, Remijsen M, Hijman R, de Haan L, van Meijel B, et al. The five-factor model of the Positive and Negative Syndrome Scale II: a ten-fold cross-validation of a revised model. Schizophr Res. 2006;85(1-3):280-7.

30. Wallwork RS, Fortgang R, Hashimoto R, Weinberger DR, Dickinson D. Searching for a consensus five-factor model of the Positive and Negative Syndrome Scale for schizophrenia. Schizophr Res. 2012;137(1-3):246-50.

31. Fong TC, Ho RT, Wan AH, Siu PJ, Au-Yeung FS. Psychometric validation of the consensus five-factor model of the Positive and Negative Syndrome Scale. Compr Psychiatry. 2015;62:204-8.

32. Munoz-Negro JE, Ibanez-Casas I, de Portugal E, Ochoa S, Dolz M, Haro JM, et al. A dimensional comparison between delusional disorder, schizophrenia and schizoaffective disorder. Schizophr Res. 2015;169(1-3):248-54.

33. Grover S, Dua D, Chakrabarti S, Avasthi A. Factor analysis of symptom dimensions (psychotic, affective and obsessive compulsive symptoms) in schizophrenia. Asian J Psychiatr. 2018;38:72-7.

34. Shafer A, Dazzi F. Meta-analysis of the positive and Negative Syndrome Scale (PANSS) factor structure. J Psychiatr Res. 2019;115:113-20.

35. Gur RE, Mozley PD, Resnick SM, Levick S, Erwin R, Saykin AJ, et al. Relations among clinical scales in schizophrenia. Am J Psychiatry. 1991;148(4):472-8.

36. Arndt S, Alliger RJ, Andreasen NC. The distinction of positive and negative symptoms. The failure of a two-dimensional model. Br J Psychiatry. 1991;158:317-22.

37. Goldman RS, Tandon R, Liberzon I, Goodson J, Greden JF. Stability of positive and negative symptom constructs during neuroleptic treatment in schizophrenia. Psychopathology. 1991;24(4):247-52.

38. Peralta V, de Leon J, Cuesta MJ. Are there more than two syndromes in schizophrenia? A critique of the positive-negative dichotomy. Br J Psychiatry. 1992;161:335-43.

39. Brown KW, White T. Syndromes of chronic schizophrenia and some clinical correlates. Br J Psychiatry. 1992;161:317-22.

40. Minas IH, Stuart GW, Klimidis S, Jackson HJ, Singh BS, Copolov DL. Positive and negative symptoms in the psychoses: multidimensional scaling of SAPS and SANS items. Schizophr Res. 1992;8(2):143-56.

41. Malla AK, Norman RM, Williamson P, Cortese L, Diaz F. Three syndrome concept of schizophrenia. A factor analytic study. Schizophr Res. 1993;10(2):143-50.

42. Palacios-Araus L, Herran A, Sandoya M, Gonzalez de la Huebra E, Vazquez-Barquero JL, Diez-Manrique JF. Analysis of positive and negative symptoms in schizophrenia. A study from a population of long-term outpatients. Acta Psychiatr Scand. 1995;92(3):178-82.

43. McAdams LA, Harris MJ, Heaton SC, Bailey A, Fell R, Jeste DV. Validity of specific subscales of the positive and negative symptom scales in older schizophrenia outpatients. Schizophr Res. 1997;27(2-3):219-26.

44. Grube BS, Bilder RM, Goldman RS. Meta-analysis of symptom factors in schizophrenia. Schizophr Res. 1998;31(2-3):113-20.

45. Basso MR, Nasrallah HA, Olson SC, Bornstein RA. Neuropsychological correlates of negative, disorganized and psychotic symptoms in schizophrenia. Schizophr Res. 1998;31(2-3):99-111.

46. Peralta V, Cuesta MJ. Dimensional structure of psychotic symptoms: an item-level analysis of SAPS and SANS symptoms in psychotic disorders. Schizophr Res. 1999;38:13-26.

47. Cardno AG, Jones LA, Murphy KC, Sanders RD, P. A, Owen MO, et al. Dimensions of Psychosis in Affected Sibling Pairs. Schizophr Bull. 1999;25(4):841-50.

48. Emsley RA, Niehaus DJH, Mbanga NI, Oosthuizen PP, Stein DJ, Maritz JS, et al. The factor structure for positive and negative symptoms in South African Xhosa patients with schizophrenia. Schizophr Res. 2001;47(2-3):149-57.

49. Peralta V, Cuesta MJ. How many and which are the psychopathological dimensions in schizophrenia? Issues influencing their ascertainment. Schizophr Res. 2001;49(3):269-85.

50. Kulhara P, Avasthi A. Influence of depressive symptoms and premorbid adjustment on factor structure of phenomenology of schizophrenia: a study from India. Eur Psychiat. 2003;18(5):226-32.

51. Andreasen NC, Arndt S, Miller D, Flaum M, Nopoulos P. Correlational Studies of the Scale for the Assessment of Negative Symptoms and the Scale for the Assessment of Positive Symptoms - an Overview and Update. Psychopathology. 1995;28(1):7-17.

52. Niehaus DJH, Koen L, Laurent C, Muller J, Deleuze JF, Mallet J, et al. Positive and negative symptoms in affected sib pairs with schizophrenia: Implications for genetic studies in an African Xhosa sample. Schizophr Res. 2005;79(2-3):239-49.

53. Mueser KT, Curren PJ, McHugo GJ. Factor structure of the brief psychiatric rating scale in schizophrenia. Psychol Assessment. 1997;9(3):196-204.

54. Long JD, Brekke JS. Longitudinal factor structure of the Brief Psychiatric Rating Scale in schizophrenia. Psychol Assessment. 1999;11(4):498-506.

55. Shafer A. Meta-analysis of the Brief Psychiatric Rating Scale factor structure. Psychol Assessment. 2005;17(3):324-35.

56. Colasanti A, Paletta S, Moliterno D, Mazzocchi A, Mauri MC, Altamura AC. Symptom dimensions as predictors of clinical outcome, duration of hospitalization, and aggressive behaviours in acutely hospitalized patients with psychotic exacerbation. Clin Pract Epidemiol Ment Health. 2010;6:72-8.

57. Picardi A, Viroli C, Tarsitani L, Miglio R, de Girolamo G, Dell'Acqua G, et al. Heterogeneity and symptom structure of schizophrenia. Psychiatry Res. 2012;198(3):386-94.

58. Blanchard JJ, Cohen AS. The structure of negative symptoms within schizophrenia: implications for assessment. Schizophr Bull. 2006;32(2):238-45.

59. Marder SR, Galderisi S. The current conceptualization of negative symptoms in schizophrenia. World Psychiatry. 2017;16(1):14-24.

60. Strauss GP, Ahmed AO, Young JW, Kirkpatrick B. Reconsidering the Latent Structure of Negative Symptoms in Schizophrenia: A Review of Evidence Supporting the 5 Consensus Domains. Schizophr Bull. 2019;45(4):725-9.

61. Gibbons RD, Lewine RR, Davis JM, Schooler NR, Cole JO. An empirical test of a Kraepelinian vs. a Bleulerian view of negative symptoms. Schizophr Bull. 1985;11(3):390-6.

62. Axelrod BN, Goldman RS, Woodard JL, Alphs LD. Factor Structure of the Negative Symptom Assessment. Psychiatry Res. 1994;52:173-9.

63. Keefe RS, Harvey PD, Lenzenweger MF, Davidson M, Apter SH, Schmeidler J, et al. Empirical assessment of the factorial structure of clinical symptoms in schizophrenia: negative symptoms. Psychiatry Res. 1992;44(2):153-65.

64. Mueser KT, Sayers SL, Schooler NR, Mance RM, Haas GL. A multisite investigation of the reliability of the Scale for the Assessment of Negative Symptoms. Am J Psychiatry. 1994;151(10):1453-62.

65. Peralta V, Cuesta MJ. Negative symptoms in schizophrenia: a confirmatory factor analysis of competing models. Am J Psychiatry. 1995;152(10):1450-7.

66. Sayers SL. Factor structure and construct validity of the Scale for the Assessment of Negative Symptoms. . Psychol Assess 1996;8:269–89.

67. Kelley ME, van Kammen DP, Allen DN. Empirical validation of primary negative symptoms: independence from effects of medication and psychosis. Am J Psychiatry. 1999;156(3):406-11.

68. Strauss GP, Horan WP, Kirkpatrick B, Fischer BA, Keller WR, Miski P, et al. Deconstructing negative symptoms of schizophrenia: avolition-apathy and diminished expression clusters predict clinical presentation and functional outcome. J Psychiatr Res. 2013;47(6):783-90.

69. Ergul C, Ucok A. Negative symptom subgroups have different effects on the clinical course of schizophrenia after the first episode: a 24-month follow up study. Eur Psychiatry. 2015;30(1):14-9.

70. Liemburg E, Castelein S, Stewart R, van der Gaag M, Aleman A, Knegtering H, et al. Two subdomains of negative symptoms in psychotic disorders: established and confirmed in two large cohorts. J Psychiatr Res. 2013;47(6):718-25.

71. Stiekema AP, Liemburg EJ, van der Meer L, Castelein S, Stewart R, van Weeghel J, et al. Confirmatory Factor Analysis and Differential Relationships of the Two Subdomains of Negative Symptoms in Chronically Ill Psychotic Patients. PLoS One. 2016;11(2):e0149785.

72. Jang SK, Choi HI, Park S, Jaekal E, Lee GY, Cho YI, et al. A Two-Factor Model Better Explains Heterogeneity in Negative Symptoms: Evidence from the Positive and Negative Syndrome Scale. Front Psychol. 2016;7:707.

73. Kimhy D, Yale S, Goetz RR, McFarr LM, Malaspina D. The factorial structure of the schedule for the deficit syndrome in schizophrenia. Schizophr Bull. 2006;32(2):274-8.

74. Nakaya M, Ohmori K. A two-factor structure for the Schedule for the Deficit Syndrome in schizophrenia. Psychiatry Res. 2008;158(2):256-9.

75. Kirkpatrick B, Buchanan RW, McKenney PD, Alphs LD, Carpenter WT, Jr. The Schedule for the Deficit syndrome: an instrument for research in schizophrenia. Psychiatry Res. 1989;30(2):119-23.

76. Galderisi S, Bucci P, Mucci A, Kirkpatrick B, Pini S, Rossi A, et al. Categorical and dimensional approaches to negative symptoms of schizophrenia: focus on long-term stability and functional outcome. Schizophr Res. 2013;147(1):157-62.

77. Peralta V, Moreno-Izco L, Sanchez-Torres A, Garcia de Jalon E, Campos MS, Cuesta MJ. Characterization of the deficit syndrome in drug-naive schizophrenia patients: the role of spontaneous movement disorders and neurological soft signs. Schizophr Bull. 2014;40(1):214-24.

78. Horan WP, Kring AM, Gur RE, Reise SP, Blanchard JJ. Development and psychometric validation of the Clinical Assessment Interview for Negative Symptoms (CAINS). Schizophr Res. 2011;132(2-3):140-5.

79. Kring AM, Gur RE, Blanchard JJ, Horan WP, Reise SP. The Clinical Assessment Interview for Negative Symptoms (CAINS): final development and validation. Am J Psychiatry. 2013;170(2):165-72.

80. Engel M, Fritzsche A, Lincoln TM. Validation of the German version of the Clinical Assessment Interview for Negative Symptoms (CAINS). Psychiatry Res. 2014;220(1-2):659-63.

81. Blanchard JJ, Bradshaw KR, Garcia CP, Nasrallah HA, Harvey PD, Casey D, et al. Examining the reliability and validity of the Clinical Assessment Interview for Negative Symptoms within the Management of Schizophrenia in Clinical Practice (MOSAIC) multisite national study. Schizophr Res. 2017;185:137-43.

82. Rekhi G, Ang MS, Yuen CKY, Ng WY, Lee J. Assessing negative symptoms in schizophrenia: Validity of the clinical assessment interview for negative symptoms in Singapore. Schizophr Res. 2019;206:177-82.

83. Kirkpatrick B, Strauss GP, Nguyen L, Fischer BA, Daniel DG, Cienfuegos A, et al. The brief negative symptom scale: psychometric properties. Schizophr Bull. 2011;37(2):300-5.

84. Strauss GP, Hong LE, Gold JM, Buchanan RW, McMahon RP, Keller WR, et al. Factor structure of the Brief Negative Symptom Scale. Schizophr Res. 2012;142(1-3):96-8.

85. Mucci A, Galderisi S, Merlotti E, Rossi A, Rocca P, Bucci P, et al. The Brief Negative Symptom Scale (BNSS): Independent validation in a large sample of Italian patients with schizophrenia. Eur Psychiatry. 2015;30(5):641-7.

86. Garcia-Portilla MP, Garcia-Alvarez L, Mane A, Garcia-Rizo C, Sugranyes G, Berge D, et al. The negative syndrome of schizophrenia: three -underlying components are better than two. Schizophr Res. 2015;166(1-3):115-8.

87. de Medeiros HLV, Vasconcelos SC, Elkis H, Martins DR, de Alexandria Leite RM, de Albuquerque ACL, et al. The Brief Negative Symptom Scale: Validation in a multicenter Brazilian study. Compr Psychiatry. 2018;85:42-7.

88. Ahmed AO, Kirkpatrick B, Galderisi S, Mucci A, Rossi A, Bertolino A, et al. Cross-cultural Validation of the 5-Factor Structure of Negative Symptoms in Schizophrenia. Schizophr Bull. 2019;45(2):305-14.

89. Strauss GP, Nunez A, Ahmed AO, Barchard KA, Granholm E, Kirkpatrick B, et al. The Latent Structure of Negative Symptoms in Schizophrenia. JAMA Psychiatry. 2018;75(12):1271-9.

90. Strauss GP, Esfahlani FZ, Galderisi S, Mucci A, Rossi A, Bucci P, et al. Network Analysis Reveals the Latent Structure of Negative Symptoms in Schizophrenia. Schizophr Bull. 2018.

91. Mucci A, Vignapiano A, Bitter I, Austin SF, Delouche C, Dollfus S, et al. A large European, multicenter, multinational validation study of the Brief Negative Symptom Scale. Eur Neuropsychopharmacol. 2019.

92. Venables PH. A short scale for rating activity-withdrawal in schizophrenics. J Ment Sci. 1957;103(430):197-9.

93. Overall JE, Hollister LE, Pichot P. Major psychiatric disorders. A four-dimensional model. Arch Gen Psychiatry. 1967;16(2):146-51.

94. Abrams R, Taylor MA. A rating scale for emotional blunting. Am J Psychiatry. 1978;135(2):226-9.

95. Iager AC, Kirch DG, Wyatt RJ. A Negative Symptom Rating Scale. Psychiatry Res. 1985;16(1):27-36.

96. Kay SR, Opler LA, Lindenmayer JP. The Positive and Negative Syndrome Scale (PANSS): rationale and standardisation. Br J Psychiatry Suppl. 1989(7):59-67.

97. Liddle PF, Barnes TR. The subjective experience of deficits in schizophrenia. Compr Psychiatry. 1988;29(2):157-64.

98. Andreasen NC. The Scale for the Assessment of Negative Symptoms (SANS): conceptual and theoretical foundations. Br J Psychiatry Suppl. 1989(7):49-58.

99. Alphs LD, Summerfelt A, Lann H, Muller RJ. The negative symptom assessment: a new instrument to assess negative symptoms of schizophrenia. Psychopharmacol Bull. 1989;25(2):159-63.

100. Mortimer AM, McKenna PJ, Lund CE, Mannuzza S. Rating of negative symptoms using the High Royds Evaluation of Negativity (HEN) scale. Br J Psychiatry Suppl. 1989(7):89-92.

101. Mortimer AM, McKenna PJ, Lund CE, Mannuzza S. High Royds Evaluation of Negativity (HEN III). The British Journal of Psychiatry. 1989;155(Suppl 7):92-.

102. Jaeger J, Bitter I, Czobor P, Volavka J. The measurement of subjective experience in schizophrenia: the Subjective Deficit Syndrome Scale. Compr Psychiatry. 1990;31(3):216-26.

103. Selten JP, Sijben NE, van den Bosch RJ, Omloo-Visser J, Warmerdam H. The subjective experience of negative symptoms: a self-rating scale. Compr Psychiatry. 1993;34(3):192-7.

104. Tremeau F, Goggin M, Antonius D, Czobor P, Hill V, Citrome L. A new rating scale for negative symptoms: the Motor-Affective-Social Scale. Psychiatry Res. 2008;160(3):346-55.

105. Llerena K, Park SG, McCarthy JM, Couture SM, Bennett ME, Blanchard JJ. The Motivation and Pleasure Scale-Self-Report (MAP-SR): reliability and validity of a self-report measure of negative symptoms. Compr Psychiatry. 2013;54(5):568-74.

106. Dollfus S, Mach C, Morello R. Self-Evaluation of Negative Symptoms: A Novel Tool to Assess Negative Symptoms. Schizophr Bull. 2016;42(3):571-8.

107. Blanchard JJ, Kring AM, Horan WP, Gur R. Toward the next generation of negative symptom assessments: the collaboration to advance negative symptom assessment in schizophrenia. Schizophr Bull. 2011;37(2):291-9.

108. Kay SR, Fiszbein A, Opler LA. The positive and negative syndrome scale (PANSS) for schizophrenia. Schizophr Bull. 1987;13(2):261-76.

109. Forbes C, Blanchard JJ, Bennett M, Horan WP, Kring A, Gur R. Initial development and preliminary validation of a new negative symptom measure: the Clinical Assessment Interview for Negative Symptoms (CAINS). Schizophr Res. 2010;124(1-3):36-42.

110. Daniel DG, Alphs L, Cazorla P, Bartko JJ, Panagides J. Training for assessment of negative symptoms of schizophrenia across languages and cultures: comparison of the NSA-16 with the PANSS Negative Subscale and Negative Symptom factor. Clin Schizophr Relat Psychoses. 2011;5(2):87-94.

111. Axelrod BN, Goldman RS, Alphs LD. Validation of the 16-item Negative Symptom Assessment. J Psychiatr Res. 1993;27(3):253-8.

112. Velligan DI, Alphs L, Lancaster S, Morlock R, Mintz J. Association between changes on the Negative Symptom Assessment scale (NSA-16) and measures of functional outcome in schizophrenia. Psychiatry Res. 2009;169(2):97-100.

113. Alphs L, Morlock R, Coon C, Cazorla P, Szegedi A, Panagides J. Validation of a 4-item Negative Symptom Assessment (NSA-4): a short, practical clinical tool for the assessment of negative symptoms in schizophrenia. Int J Methods Psychiatr Res. 2011;20(2):e31-7.

114. Strauss GP, Keller WR, Buchanan RW, Gold JM, Fischer BA, McMahon RP, et al. Next-generation negative symptom assessment for clinical trials: validation of the Brief Negative Symptom Scale. Schizophr Res. 2012;142(1-3):88-92.

115. Mane A, Garcia-Rizo C, Garcia-Portilla MP, Berge D, Sugranyes G, Garcia-Alvarez L, et al. Spanish adaptation and validation of the Brief Negative Symptoms Scale. Compr Psychiatry. 2014;55(7):1726-9.

116. Bischof M, Obermann C, Hartmann MN, Hager OM, Kirschner M, Kluge A, et al. The brief negative symptom scale: validation of the German translation and convergent validity with self-rated anhedonia and observer-rated apathy. BMC Psychiatry. 2016;16(1):415.

117. Polat Nazli I, Ergul C, Aydemir O, Chandhoke S, Ucok A, Gonul AS. Validation of Turkish version of brief negative symptom scale. Int J Psychiatry Clin Pract. 2016;20(4):265-71.

118. Strauss GP, Gold JM. A Psychometric Comparison of the Clinical Assessment Interview for Negative Symptoms and the Brief Negative Symptom Scale. Schizophr Bull. 2016;42(6):1384-94.

119. Kirkpatrick B, Saoud JB, Strauss GP, Ahmed AO, Tatsumi K, Opler M, et al. The brief negative symptom scale (BNSS): Sensitivity to treatment effects. Schizophr Res. 2017.

120. Ang MS, Rekhi G, Lee J. Validation of the Brief Negative Symptom Scale and its association with functioning. Schizophr Res. 2019;208:97-104.

121. Gehr J, Glenthoj B, Odegaard Nielsen M. Validation of the Danish version of the brief negative symptom scale. Nord J Psychiatry. 2019;73(7):425-32.

122. Treen D, Savulich G, Mezquida G, Garcia-Portilla MP, Toll A, Garcia-Rizo C, et al. Influence of secondary sources in the Brief Negative Symptom Scale. Schizophr Res. 2019;204:452-4.

123. Wojciak P, Gorna K, Domowicz K, Jaracz K, Golebiewska K, Michalak M, et al. Polish version of the Brief Negative Symptom Scale (BNSS). Psychiatr Pol. 2019;53(3):541-9.

124. Tatsumi K, Kirkpatrick B, Strauss GP, Opler M. The brief negative symptom scale in translation: A review of psychometric properties and beyond. Eur Neuropsychopharmacol. 2020.

125. de Medeiros HL, Silva AM, Rodig RM, Souza SL, Sougey EB, Vasconcelos SC, et al. Cross-cultural adaptation, reliability, and content validity of the Brief Negative Symptom Scale (BNSS) for use in Brazil. Arch Clin. 2019;46(5):132-6.

126. Chan RC, Shi C, Lui SS, Ho KK, Hung KS, Lam JW, et al. Validation of the Chinese version of the Clinical Assessment Interview for Negative Symptoms (CAINS): a preliminary report. Front Psychol. 2015;6:7.

127. Valiente-Gomez A, Mezquida G, Romaguera A, Vilardebo I, Andres H, Granados B, et al. Validation of the Spanish version of the Clinical Assessment for Negative Symptoms (CAINS). Schizophr Res. 2015;166(1-3):104-9.

128. Jung SI, Woo J, Kim YT, Kwak SG. Validation of the Korean-Version of the Clinical Assessment Interview for Negative Symptoms of Schizophrenia (CAINS). J Korean Med Sci. 2016;31(7):1114-20.

129. Jang SK, Park SC, Choi KH, Yi JS, Park JK, Lee JS, et al. Validation of the Korean Version of the Clinical Assessment Interview for Negative Symptoms. Psychiatry Investig. 2017;14(4):413-9.

130. Xie DJ, Shi HS, Lui SSY, Shi C, Li Y, Ho KKY, et al. Cross Cultural Validation and Extension of the Clinical Assessment Interview for Negative Symptoms (CAINS) in the Chinese Context: Evidence from a Spectrum Perspective. Schizophr Bull. 2018;44(suppl_2):S547-S55.

131. Engel M, Lincoln TM. Motivation and Pleasure Scale-Self-Report (MAP-SR): Validation of the German version of a self-report measure for screening negative symptoms in schizophrenia. Compr Psychiatry. 2016;65:110-5.

132. Kim JS, Jang SK, Park SC, Yi JS, Park JK, Lee JS, et al. Measuring negative symptoms in patients with schizophrenia: reliability and validity of the Korean version of the Motivation and Pleasure Scale-Self-Report. Neuropsychiatr Dis Treat. 2016;12:1167-72.

133. Engel M, Lincoln TM. Concordance of self- and observer-rated motivation and pleasure in patients with negative symptoms and healthy controls. Psychiatry Res. 2017;247:1-5.

134. Dollfus S, Delouche C, Hervochon C, Mach C, Bourgeois V, Rotharmel M, et al. Specificity and sensitivity of the Self-assessment of Negative Symptoms (SNS) in patients with schizophrenia. Schizophr Res. 2019;211:51-5.

135. Rodriguez-Testal JF, Perona-Garcelan S, Dollfus S, Valdes-Diaz M, Garcia-Martinez J, Ruiz-Veguilla M, et al. Spanish validation of the self-evaluation of negative symptoms scale SNS in an adolescent population. BMC Psychiatry. 2019;19(1):327.

136. Dollfus S, Vandevelde A. The patients' view: Self-evaluation of negative symptoms. In: Bitter I, editor. Managing negative symptoms in schizophrenia. New-York: Oxford university Press; 2020.

137. Garcia-Portilla MP, Garcia-Alvarez L, Saiz PA, Al-Halabi S, Bobes-Bascaran MT, Bascaran MT, et al. Psychometric evaluation of the negative syndrome of schizophrenia. Eur Arch Psychiatry Clin Neurosci. 2015;265(7):559-66.

138. Kumari S, Malik M, Florival C, Manalai P, Sonje S. An Assessment of Five (PANSS, SAPS, SANS, NSA-16, CGI-SCH) commonly used Symptoms Rating Scales in Schizophrenia and Comparison to Newer Scales (CAINS, BNSS). J Addict Res Ther. 2017;8(3).

139. Obermeier M, Schennach-Wolff R, Meyer S, Moller HJ, Riedel M, Krause D, et al. Is the PANSS used correctly? a systematic review. BMC Psychiatry. 2011;11:113.

140. Lincoln TM, Dollfus S, Lyne J. Current developments and challenges in the assessment of negative symptoms. Schizophr Res. 2017;186:8-18.

141. Marder SR, Kirkpatrick B. Defining and measuring negative symptoms of schizophrenia in clinical trials. Eur Neuropsychopharmacol. 2014;24(5):737-43.

142. Richter J, Hesse K, Eberle M-C, Eckstein KN, Zimmermann L, Schreiber L, et al. Self-assessment of negative symptoms – Critical appraisal of the motivation and pleasure – Self-report's (MAP-SR) validity and reliability. Comprehensive Psychiatry. 2019;88:22-8.

143. Blanchard JJ, Shan LA, Andrea A, Savage C, Kring AM, Weittenhiller L. Negative symptoms and their assessment in schizophrenia and related disorders. In: Badcock JC PG, editor. A clinical introduction to psychosis : foundations for clinical psychologists and neuropsychologists2020. p. 153-75.

144. Faerden A, Nesvag R, Barrett EA, Agartz I, Finset A, Friis S, et al. Assessing apathy: the use of the Apathy Evaluation Scale in first episode psychosis. Eur Psychiatry. 2008;23(1):33-9.

145. Clarke DE, Ko JY, Kuhl EA, van Reekum R, Salvador R, Marin RS. Are the available apathy measures reliable and valid? A review of the psychometric evidence. J Psychosom Res. 2011;70(1):73-97.

146. Fervaha G, Foussias G, Takeuchi H, Agid O, Remington G. Measuring motivation in people with schizophrenia. Schizophr Res. 2015;169(1-3):423-6.

147. Faerden A, Lyngstad SH, Simonsen C, Ringen PA, Papsuev O, Dieset I, et al. Reliability and validity of the self-report version of the apathy evaluation scale in first-episode Psychosis: Concordance with the clinical version at baseline and 12 months follow-up. Psychiatry Res. 2018;267:140-7.

148. Hartmann-Riemer M, Kirschner M, Kaiser S. Effort-based decision-making paradigms as objective measures of apathy in schizophrenia? Current Opinion in Behavioral Sciences. 2018;22:70-5.

149. Luther L, Firmin RL, Lysaker PH, Minor KS, Salyers MP. A meta-analytic review of self-reported, clinician-rated, and performance-based motivation measures in schizophrenia: Are we measuring the same "stuff"? Clin Psychol Rev. 2018;61:24-37.

150. McGorry PD, Yung AR, Phillips LJ, Yuen HP, Francey S, Cosgrave EM, et al. Randomized controlled trial of interventions designed to reduce the risk of progression to first-episode psychosis in a clinical sample with subthreshold symptoms. Arch Gen Psychiatry. 2002;59(10):921-8.

151. Morrison AP, French P, Walford L, Lewis SW, Kilcommons A, Green J, et al. Cognitive therapy for the prevention of psychosis in people at ultra-high risk: randomised controlled trial. Br J Psychiatry. 2004;185:291-7.

152. McGlashan TH, Zipursky RB, Perkins D, Addington J, Miller T, Woods SW, et al. Randomized, double-blind trial of olanzapine versus placebo in patients prodromally symptomatic for psychosis. Am J Psychiatry. 2006;163(5):790-9.

153. Ruhrmann S, Bechdolf A, Kuhn KU, Wagner M, Schultze-Lutter F, Janssen B, et al. Acute effects of treatment for prodromal symptoms for people putatively in a late initial prodromal state of psychosis. Br J Psychiatry Suppl. 2007;51:s88-95.

154. Berger GE, Wood SJ, Ross M, Hamer CA, Wellard RM, Pell G, et al. Neuroprotective effects of low-dose lithium in individuals at ultra-high risk for psychosis. A longitudinal MRI/MRS study. Current pharmaceutical design. 2012;18(4):570-5.

155. Kobayashi H, Morita K, Takeshi K, Koshikawa H, Yamazawa R, Kashima H, et al. Effects of aripiprazole on insight and subjective experience in individuals with an at-risk mental state. J Clin Psychopharmacol. 2009;29(5):421-5.

156. Amminger GP, Schafer MR, Papageorgiou K, Klier CM, Cotton SM, Harrigan SM, et al. Long-chain omega-3 fatty acids for indicated prevention of psychotic disorders: a randomized, placebo-controlled trial. Arch Gen Psychiatry. 2010;67(2):146-54.

157. Addington J, Epstein I, Liu L, French P, Boydell KM, Zipursky RB. A randomized controlled trial of cognitive behavioral therapy for individuals at clinical high risk of psychosis. Schizophr Res. 2011;125(1):54-61.

158. Bechdolf A, Wagner M, Ruhrmann S, Harrigan S, Putzfeld V, Pukrop R, et al. Preventing progression to first-episode psychosis in early initial prodromal states. Br J Psychiatry. 2012;200(1):22-9.

159. van der Gaag M, Nieman DH, Rietdijk J, Dragt S, Ising HK, Klaassen RM, et al. Cognitive behavioral therapy for subjects at ultrahigh risk for developing psychosis: a randomized controlled clinical trial. Schizophr Bull. 2012;38(6):1180-8.

160. McGorry PD, Nelson B, Phillips LJ, Yuen HP, Francey SM, Thampi A, et al. Randomized controlled trial of interventions for young people at ultra-high risk of psychosis: twelve-month outcome. J Clin Psychiatry. 2013;74(4):349-56.

161. Washida K, Takeda T, Habara T, Sato S, Oka T, Tanaka M, et al. Efficacy of second-generation antipsychotics in patients at ultra-high risk and those with first-episode or multi-episode schizophrenia. Neuropsychiatr Dis Treat. 2013;9:861-8.

162. Miklowitz DJ, O'Brien MP, Schlosser DA, Addington J, Candan KA, Marshall C, et al. Family-focused treatment for adolescents and young adults at high risk for psychosis: results of a randomized trial. Journal of the American Academy of Child and Adolescent Psychiatry. 2014;53(8):848-58.

163. Fusar-Poli P, Frascarelli M, Valmaggia L, Byrne M, Stahl D, Rocchetti M, et al. Antidepressant, antipsychotic and psychological interventions in subjects at high clinical risk for psychosis: OASIS 6-year naturalistic study. Psychol Med. 2015;45(6):1327-39.

164. Kantrowitz JT, Woods SW, Petkova E, Cornblatt B, Corcoran CM, Chen H, et al. D-serine for the treatment of negative symptoms in individuals at clinical high risk of schizophrenia: a pilot, double-blind, placebo-controlled, randomised parallel group mechanistic proof-of-concept trial. Lancet Psychiatry. 2015;2(5):403-12.

165. McFarlane WR, Levin B, Travis L, Lucas FL, Lynch S, Verdi M, et al. Clinical and functional outcomes after 2 years in the early detection and intervention for the prevention of psychosis multisite effectiveness trial. Schizophr Bull. 2015;41(1):30-43.

166. Piskulic D, Barbato M, Liu L, Addington J. Pilot study of cognitive remediation therapy on cognition in young people at clinical high risk of psychosis. Psychiatry Res. 2015;225(1-2):93-8.

167. Choi J, Corcoran CM, Fiszdon JM, Stevens M, Javitt DC, Deasy M, et al. Pupillometer-based neurofeedback cognitive training to improve processing speed and social functioning in individuals at clinical high risk for psychosis. Psychiatr Rehabil J. 2017;40(1):33-42.

168. Ising HK, Kraan TC, Rietdijk J, Dragt S, Klaassen RM, Boonstra N, et al. Four-Year Follow-up of Cognitive Behavioral Therapy in Persons at Ultra-High Risk for Developing Psychosis: The Dutch Early Detection Intervention Evaluation (EDIE-NL) Trial. Schizophr Bull. 2016;42(5):1243-52.

169. Loewy R, Fisher M, Schlosser DA, Biagianti B, Stuart B, Mathalon DH, et al. Intensive Auditory Cognitive Training Improves Verbal Memory in Adolescents and Young Adults at Clinical High Risk for Psychosis. Schizophr Bull. 2016;42 Suppl 1:S118-26.

170. McGorry PD, Nelson B, Markulev C, Yuen HP, Schafer MR, Mossaheb N, et al. Effect of omega-3 Polyunsaturated Fatty Acids in Young People at Ultrahigh Risk for Psychotic Disorders: The NEURAPRO Randomized Clinical Trial. JAMA Psychiatry. 2017;74(1):19-27.

171. Kirschner M, Aleman A, Kaiser S. Secondary negative symptoms - A review of mechanisms, assessment and treatment. Schizophr Res. 2017;186:29-38.

172. Chen L, Johnston JA, Kinon BJ, Stauffer V, Succop P, Marques TR, et al. The longitudinal interplay between negative and positive symptom trajectories in patients under antipsychotic treatment: a post hoc analysis of data from a randomized, 1-year pragmatic trial. BMC Psychiatry. 2013;13:320.

173. Czobor P, Volavka J. Dimensions of the Brief Psychiatric Rating Scale: an examination of stability during haloperidol treatment. Compr Psychiatry. 1996;37(3):205-15.

174. Marder SR, Meibach RC. Risperidone in the treatment of schizophrenia. Am J Psychiatry. 1994;151(6):825-35.

175. Miller DD, Flaum M, Arndt S, Fleming F, Andreasen NC. Effect of antipsychotic withdrawal on negative symptoms in schizophrenia. Neuropsychopharmacology. 1994;11(1):11-20.

176. Miller DD, Perry PJ, Cadoret RJ, Andreasen NC. Clozapine's effect on negative symptoms in treatment-refractory schizophrenics. Compr Psychiatry. 1994;35(1):8-15.

177. Pickar D, Owen RR, Litman RE, Konicki E, Gutierrez R, Rapaport MH. Clinical and biologic response to clozapine in patients with schizophrenia. Crossover comparison with fluphenazine. Arch Gen Psychiatry. 1992;49(5):345-53.

178. Sanger TM, Lieberman JA, Tohen M, Grundy S, Beasley C, Jr., Tollefson GD. Olanzapine versus haloperidol treatment in first-episode psychosis. Am J Psychiatry. 1999;156(1):79-87.

179. Tandon R, Goldman R, DeQuardo JR, Goldman M, Perez M, Jibson M. POSITIVE AND NEGATIVE SYMPTOMS COVARY DURING CLOZAPINE TREATMENT IN SCHIZOPHRENIA. J Psychiat Res. 1993;27:341-47.

180. Tandon R, Ribeiro SC, DeQuardo JR, Goldman RS, Goodson J, Greden JF. Covariance of positive and negative symptoms during neuroleptic treatment in schizophrenia: a replication. Biol Psychiatry. 1993;34(7):495-7.

181. Tollefson GD, Sanger TM. Negative symptoms: a path analytic approach to a double-blind, placebo- and haloperidol-controlled clinical trial with olanzapine. Am J Psychiatry. 1997;154(4):466-74.

182. Carpenter WT, Jr., Heinrichs DW, Alphs LD. Treatment of negative symptoms. Schizophr Bull. 1985;11(3):440-52.

183. Moller HJ. Clinical evaluation of negative symptoms in schizophrenia. Eur Psychiatry. 2007;22(6):380-6.

184. Flaum M, Andreasen N. The reliability of distinguishing primary versus secondary negative symptoms. Compr Psychiatry. 1995;36(6):421-7.

185. Czobor P, Volavka J. Positive and negative symptoms: is their change related? Schizophr Bull. 1996;22(4):577-90.

186. Tollefson GD, Beasley CM, Jr., Tran PV, Street JS, Krueger JA, Tamura RN, et al. Olanzapine versus haloperidol in the treatment of schizophrenia and schizoaffective and schizophreniform disorders: results of an international collaborative trial. Am J Psychiatry. 1997;154(4):457-65.

187. Peralta V, Cuesta MJ, Martinez-Larrea A, Serrano JF. Differentiating primary from secondary negative symptoms in schizophrenia: a study of neuroleptic-naive patients before and after treatment. Am J Psychiatry. 2000;157(9):1461-6.

188. Johnson PB, Ramirez PM, Opler LA, Malgady R. Relationship between neuroleptic dose and positive and negative symptoms. Psychol Rep. 1994;74(2):481-2.

189. Allan ER, Sison CE, Alpert M, Connolly B, Crichton J. The relationship between negative symptoms of schizophrenia and extrapyramidal side effects with haloperidol and olanzapine. Psychopharmacol Bull. 1998;34(1):71-4.

190. Lako IM, Bruggeman R, Knegtering H, Wiersma D, Schoevers RA, Slooff CJ, et al. A systematic review of instruments to measure depressive symptoms in patients with schizophrenia. J Affect Disord. 2012;140(1):38-47.

191. Mulholland C, Cooper S. The symptom of depression in schizophrenia and its management. . Advances Psychiat Treat 2000;6(169-177).

192. Kirkpatrick B. Recognizing primary vs secondary negative symptoms and apathy vs expression domains. J Clin Psychiatry. 2014;75(4):e09.

193. Buckley PF, Miller BJ, Lehrer DS, Castle DJ. Psychiatric comorbidities and schizophrenia. Schizophr Bull. 2009;35(2):383-402.

194. Krynicki CR, Upthegrove R, Deakin JFW, Barnes TRE. The relationship between negative symptoms and depression in schizophrenia: a systematic review. Acta Psychiatrica Scandinavica. 2018;137:380-90.

195. Kulhara P, Chadda R. A study of negative symptoms in schizophrenia and depression. Compr Psychiatry. 1987;28(3):229-35.

196. Addington D, Addington J, Maticka-Tyndale E. Specificity of the Calgary Depression Scale for schizophrenics. Schizophr Res. 1994;11(3):239-44.

197. Lancon C, Auquier P, Reine G, Toumi M, Addington D. Evaluation of depression in schizophrenia: psychometric properties of a French version of the Calgary Depression Scale. Psychiatry Res. 1999;89(2):123-32.

198. Kollias CT, Kontaxakis VP, Havaki-Kontaxaki BJ, Stamouli S, Margariti M, Petridou E. Association of physical and social anhedonia with depression in the acute phase of schizophrenia. Psychopathology. 2008;41(6):365-70.

199. Liu H, Zhang H, Xiao W, Liu Q, Fu P, Chen J, et al. Scales for evaluating depressive symptoms in Chinese patients with schizophrenia. J Nerv Ment Dis. 2009;197(2):140-2.

200. Schennach R, Obermeier M, Seemuller F, Jager M, Schmauss M, Laux G, et al. Evaluating depressive symptoms in schizophrenia: a psychometric comparison of the Calgary Depression Scale for Schizophrenia and the Hamilton Depression Rating Scale. Psychopathology. 2012;45(5):276-85.

201. Lako IM, Wigman JT, Klaassen RM, Slooff CJ, Taxis K, Bartels-Velthuis AA, et al. Psychometric properties of the self-report version of the Quick Inventory of Depressive Symptoms (QIDS-SR(1)(6)) questionnaire in patients with schizophrenia. BMC Psychiatry. 2014;14:247.

202. Schennach R, Riedel M, Obermeier M, Seemuller F, Jager M, Schmauss M, et al. What are depressive symptoms in acutely ill patients with schizophrenia spectrum disorder? Eur Psychiatry. 2015;30(1):43-50.

203. Grover S, Sahoo S, Dua D, Chakrabarti S, Avasthi A. Scales for assessment of depression in schizophrenia: Factor analysis of calgary depression rating scale and hamilton depression rating scale. Psychiatry Res. 2017;252:333-9.

204. Richter J, Holz L, Hesse K, Wildgruber D, Klingberg S. Measurement of negative and depressive symptoms: Discriminatory relevance of affect and expression. Eur Psychiatry. 2019;55:23-8.
